# Equivalent running leg lengths require prosthetic legs to be longer than biological legs during standing

**DOI:** 10.1038/s41598-023-34346-x

**Published:** 2023-05-11

**Authors:** Janet H. Zhang-Lea, Joshua R. Tacca, Owen N. Beck, Paolo Taboga, Alena M. Grabowski

**Affiliations:** 1 Department of Integrative Physiology, University of Colorado Boulder, Boulder, CO USA; 2 Department of Human Physiology, Gonzaga University, Spokane, WA USA; 3 Paul M. Rady Department of Mechanical Engineering, University of Colorado Boulder, Boulder, CO USA; 4 Department of Kinesiology and Health Education, University of Texas at Austin, Austin, TX USA; 5 Department of Kinesiology, Sacramento State University, Sacramento, CA USA; 6 Department of Veterans Affairs, Eastern Colorado Healthcare System, Denver, CO USA

**Keywords:** Health policy, Musculoskeletal system

## Abstract

We aimed to determine a method for prescribing a standing prosthetic leg length (ProsL) that results in an equivalent running biological leg length (BioL) for athletes with unilateral (UTTA) and bilateral transtibial amputations (BTTA). We measured standing leg length of ten non-amputee (NA) athletes, ten athletes with UTTA, and five athletes with BTTA. All athletes performed treadmill running trials from 3 m/s to their maximum speed. We calculated standing and running BioL and ProsL lengths and assessed the running-to-standing leg length ratio (L_ratio_) at three instances during ground contact: touchdown, mid-stance, and take-off. Athletes with UTTA had 2.4 cm longer standing ProsL than BioL length (p = 0.030), but their ProsL length were up to 3.3 cm shorter at touchdown and 4.1 cm shorter at mid-stance than BioL, at 3–11.5 m/s. At touchdown, mid-stance, and take-off, athletes with BTTA had 0.01–0.05 lower L_ratio_ at 3 m/s (p < 0.001) and 0.03–0.07 lower L_ratio_ at 10 m/s (p < 0.001) in their ProsL compared to the BioL of NA athletes. During running, ProsL were consistently shorter than BioL. To achieve equivalent running leg lengths at touchdown and take-off, athletes with UTTA should set their running-specific prosthesis height so that their standing ProsL length is 2.8–4.5% longer than their BioL length, and athletes with BTTA should set their running-specific prosthesis height so that their standing ProsL lengths are at least 2.1–3.9% longer than their presumed BioL length. Setting ProsL length to match presumed biological dimensions during standing results in shorter legs during running.

## Introduction

Athletes with transtibial amputations run using running-specific prostheses (RSPs), which are compliant devices comprised of carbon fiber that attach to a socket, which encompasses the residual limb. During the stance phase of running, RSPs mimic the spring-like behavior of biological legs^1^, yet unlike biological feet and ankles, RSPs cannot actively plantar- or dorsi-flex during running. Thus, prosthetic leg geometry cannot be modulated like biological legs. For example, when running at 9 m/s, athletes with biological legs plantarflex their ankles ~ 10° at touch-down, and ~ 30° at take-off, effectively increasing leg length during running compared to standing^2,3^. However, prosthetic legs of athletes with transtibial amputations cannot be actively lengthened during running.

Prosthetists generally prescribe RSP height for athletes with unilateral transtibial amputation (UTTA) based on their hip heights during running^4^. Typically, the unloaded prosthetic leg (ProsL) length of an athlete with UTTA is prescribed to be 3–8% taller than their biological leg (BioL) length while standing^1,5–8^. Though athletes with UTTA are not restricted in their ProsL length to compete in sanctioned athletics competitions, athletes with *bilateral* transtibial amputations (BTTA) must follow strict height regulations. Under the International Paralympic Committee (IPC) rules, which have been adopted by World Athletics^9,10^, athletes with BTTA cannot use ProsLs that exceed their maximum allowable standing height (MASH). Each athlete’s MASH equals their estimated barefoot standing height using select body segment dimensions^9,11,12^. The intent of the MASH rule is to prevent athletes from setting their RSPs so that ProsL lengths are taller than presumed *standing* BioL lengths. Aside from the issues of accurately predicting an BTTA athlete’s leg length and height, MASH does not consider shoe sole thickness, or how ProsL and BioL lengths change during *running*.

Identical standing ProsL and BioL lengths may yield different running leg lengths, thus we sought to determine the standing ProsL length that elicits an equivalent BioL length during running. Accordingly, we compared absolute leg lengths (distance from the hip joint center to the distal end of the foot or RSP) during standing and the stance phase of running, and the ratio between running and standing leg length (L_ratio_, Eq. 1) across a range of speeds for non-amputee (NA) athletes and athletes with UTTA and BTTA.

1 $${L}_{ratio}=\frac{Running\,leg\,length}{Standing\,leg\,length}$$

We hypothesized that, (1) because athletes with UTTA set their standing ProsL length longer than their BioL length, their ProsL length and BioL length would not differ during the stance phase of running, and (2) because the geometry of a ProsL cannot be adjusted like that of a BioL, the ProsL L_ratio_ of athletes with BTTA would be less than the BioL L_ratio_ of NA athletes during the stance phase of running. If our hypotheses are supported, setting standing ProsL length *taller* than their presumed standing BioL length would match the intended BioL length during the stance phase of running.

## Results

### Athletes with unilateral transtibial amputation (UTTA)

**Figure 1:**
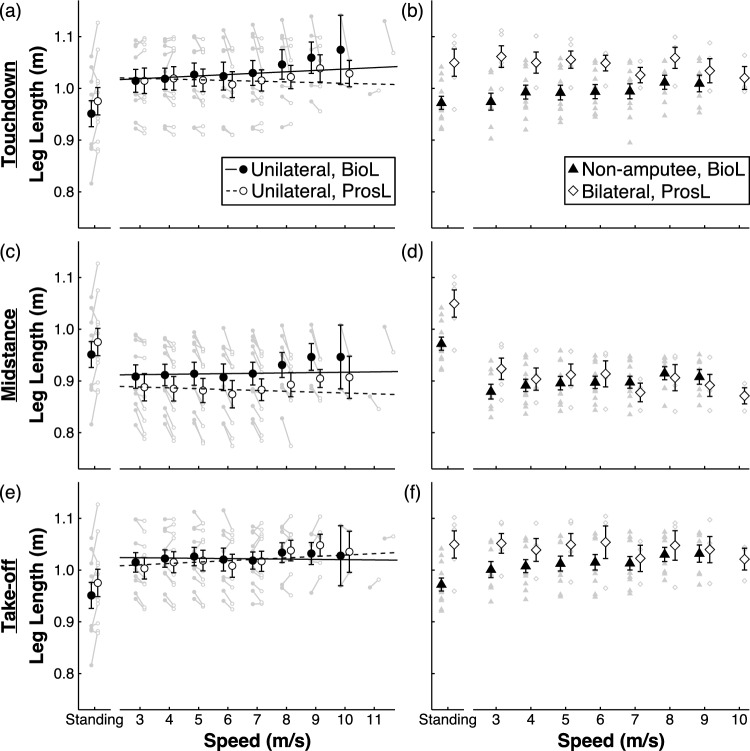
Standing and running biological leg (BioL) and prosthetic leg (ProsL) length versus running speed at touchdown (**a,b**), mid-stance (**c,d**), and take-off (**e,f**) for athletes with unilateral transtibial amputation (left column, **a,c,e**) and non-amputee athletes and athletes with bilateral transtibial amputations (right column, **b,d,f**). BioLs are solid and ProsLs are open symbols. In the left column, solid (BioL) and dashed lines (ProsL) show results of the linear mixed models. Circles indicate the mean and error bars are standard error of the mean. Symbols are offset at each speed for clarity. Each grey symbol and line represent an individual athlete’s BioL (filled) and ProsL (open) length ratio.

Athletes with UTTA had 2.4 ± 3.5 cm (average ± SD) longer standing ProsL than BioL lengths (p = 0.030, t = -2.2, Cohen’s d = 0.68; Fig. 1). The speed range of athletes with UTTA spanned 3 to 11.5 m/s. When controlling for speed and interactions, running ProsL length was 1.8–2.3 cm shorter than BioL length at mid-stance and take-off (p < 0.001), but not at touchdown (p = 0.067). We found a significant interaction effect whereby ProsL versus BioL length differences were greater at faster speed at touchdown (p < 0.001) and mid-stance (p = 0.0014), and less at faster speed at take-off (p = 0.0012). At touchdown, running ProsL and BioL lengths were similar at 3 m/s, but average ProsL length was 3.3 cm shorter than BioL length at 11.5 m/s (Fig. 1a; Table 1). At mid-stance, average ProsL length was 2.4 cm shorter at 3 m/s and 4.1 cm shorter at 11.5 m/s than BioL (Fig. 1c; Table 1). At take-off, ProsL length was 1.4 cm shorter than BioL length at 3 m/s, but 1.1 cm longer than BioL length at 11.5 m/s (Fig. 1e; Table 1).

**Table 1:** Linear mixed model parameters for fixed effects of leg type (prosthetic, assigned as 1, versus biological, assigned as 0), speed (m/s), and their interaction at touchdown, mid-stance, and take-off on leg length and leg length ratio (L_ratio_) for athletes with unilateral transtibial amputation. Coefficient estimates (B), coefficient standard errors (*SE*), t values (*t*), p values (*p*), and conditional and marginal R^2^ values for each linear mixed model are listed.

Touchdown leg length (m)	Estimate (B)	SE	t	p
Intercept	1.011	0.021	47.53	** < 0.001**
Speed [m/s]	0.003	0.001	3.50	** < 0.001**
Leg Type [Prosthetic vs. Biological]	0.013	0.007	1.85	0.067
Speed [m/s]*Leg Type [Prosthetic vs. Biological]	−0.004	0.001	−3.83	** < 0.001**
Conditional R^2^: 0.960; Marginal R^2^: 0.013				

Mid-stance leg length (m)	Estimate (B)	SE	t	p
Intercept	0.910	0.021	42.98	** < 0.001**
Speed [m/s]	0.001	0.001	1.29	0.20
Leg Type [Prosthetic vs. Biological]	−0.018	0.005	−3.66	** < 0.001**
Speed [m/s]*Leg Type [Prosthetic vs. Biological]	−0.002	0.001	−3.26	**0.001**
Conditional R^2^: 0.983; Marginal R^2^: 0.054				

Take-off leg length (m)	Estimate (B)	SE	t	p
Intercept	1.026	0.018	58.34	** < 0.001**
Speed [m/s]	−0.001	0.001	−0.75	0.45
Leg Type [Prosthetic vs. Biological]	−0.023	0.006	−3.68	** < 0.001**
Speed [m/s]*Leg Type [Prosthetic vs. Biological]	0.003	0.001	3.32	**0.001**
Conditional R^2^: 0.950; Marginal R^2^: 0.007				

Touchdown leg length (m)	Estimate (B)	SE	t	p
** L**_**ratio**_	**Estimate (B)**	**SE**	**t**	**p**
Intercept	1.065	0.013	79.64	** < 0.001**
Leg Type [Prosthetic vs. Biological]	−0.015	0.013	−1.16	0.25
Conditional R^2^: 0.679; Marginal R^2^: 0.210				

Mid-stance	Estimate (B)	SE	t	p
Intercept	0.963	0.010	101.57	** < 0.001**
Leg Type [Prosthetic vs. Biological]	−0.057	0.003	−17.67	** < 0.001**
Conditional R^2^: 0.825; Marginal R^2^: 0.405				

Take-off L_ratio_	Estimate (B)	SE	t	p
Intercept	1.078	0.011	95.52	** < 0.001**
Leg Type [Prosthetic vs. Biological]	−0.03	0.004	−7.87	** < 0.001**
Conditional R^2^: 0.725; Marginal R^2^: 0.126				

Significant values are in [bold].

**Figure 2:**
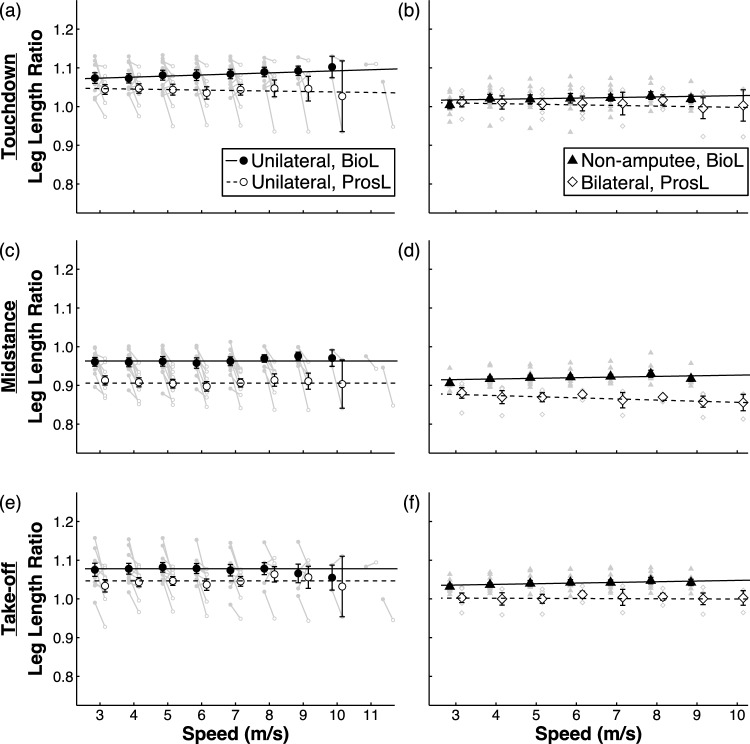
Running biological leg (BioL) and prosthetic leg (ProsL) length ratio versus running speed at touchdown (**a,b**), mid-stance (**c,d**), and take-off (**e,f**) for athletes with unilateral transtibial amputation (left column, **a,c,e**) and non-amputee athletes and athletes with bilateral transtibial amputations (right column, **b,d,f**). Solid (BioL) and dashed lines (ProsL) show results of the linear mixed models. BioLs are solid and ProsLs are open symbols. In the left column, circles indicate the mean and error bars are standard error of the mean. Symbols are offset at each speed for clarity. Each grey symbol and line represent an individual athlete’s BioL (filled) and ProsL (open) length ratio.

When controlling for leg type and interactions, we found no speed effect of L_ratio_ for leg type at touchdown (p = 0.061), mid-stance (p = 0.51), or take-off (p = 0.72, Fig. 2). Across speed, L_ratio_ was 0.057 less at mid-stance, and 0.032 less at take-off for a ProsL compared to BioL (p < 0.001, Fig. 2c,e, Table 1), but we found no difference of L_ratio_ at touchdown (p = 0.25). There was an interaction between leg type and speed on L_ratio_ at touchdown (p = 0.048) where ProsL L_ratio_ was 0.027 less at 3 m/s and 0.063 less at 11.5 m/s than BioL L_ratio_ (Fig. 2a; Table 1).

### Athletes with bilateral transtibial amputation (BTTA) and non-amputee (NA) athletes

The speed range of athletes with BTTA and NA athletes spanned 3 to 10 m/s. When controlling for speed and interactions, L_ratio_ was 0.025 less at mid-stance (p = 0.043), and 0.028 less at take-off (p = 0.026) in the ProsL of athletes with BTTA compared to the BioL of NA athletes, but not different at touchdown (p = 0.94) across speeds (Fig. 2). When controlling for leg type, speed affected L_ratio_ at mid-stance (p < 0.001), but not at touchdown (p = 0.072) and take-off (p = 0.36). We found an interaction between leg type and speed on L_ratio_ at touchdown (p = 0.010), mid-stance (p < 0.001), and take-off (p < 0.001). At touchdown, ProsL had a L_ratio_ 0.01 less at 3 m/s, and 0.03 less at 10 m/s than BioL (Fig. 2b, Table 2). At mid-stance, ProsL had a L_ratio_ 0.04 less at 3 m/s, and 0.07 less at 10 m/s than BioL (Fig. 2d, Table 2). At take-off, ProsL had a L_ratio_ 0.03 less at 3 m/s, and 0.05 less at 10 m/s than BioL (Fig. 2f, Table 2).

**Table 2:** Linear mixed model parameters for fixed effects of athlete group (bilateral, assigned as 1 vs. non-amputee, assigned as 0), speed (m/s), and speed interaction on running leg length ratio (L_ratio_) at touchdown, mid-stance, and take-off for athletes with bilateral transtibial amputations. Coefficient estimates (B), coefficient standard errors (*SE*), t values (*t*), p values (*p*), and conditional and marginal R^2^ values for each linear mixed model are listed.

Touchdown L_ratio_	*Estimate (B)*	*SE*	*t*	*p*
Intercept	1.014	0.015	70.01	** < 0.001**
Speed [m/s]	−0.002	0.001	−1.83	0.071
Athlete Group [Bilateral vs. Non-amputee]	0.002	0.018	−0.08	0.93
Speed [m/s]*Athlete Group [Bilateral vs. Non-amputee]	−0.003	0.001	2.63	**0.010**
Conditional R^2^: 0.850; Marginal R^2^: 0.082				

Mid-stance L_ratio_	*Estimate (B)*	*SE*	*t*	*p*
Intercept	0.885	0.009	94.51	** < 0.001**
Speed [m/s]	−0.003	0.000	−7.83	** < 0.001**
Athlete Group [Bilateral vs. Non-amputee]	−0.025	0.011	2.17	**0.043**
Speed [m/s]*Athlete Group [Bilateral vs. Non-amputee]	−0.005	0.001	8.91	** < 0.001**
Conditional R^2^: 0.973; Marginal R^2^: 0.616				

Take-off L_ratio_	*Estimate (B)*	*SE*	*t*	*p*
Intercept	1.003	0.009	106.29	** < 0.001**
Speed [m/s]	−0.000	0.000	−0.92	0.36
Athlete Group [Bilateral vs. Non-amputee]	−0.028	0.012	2.42	**0.026**
Speed [m/s]*Athlete Group [Bilateral vs. Non-amputee]	−0.002	0.001	4.15	** < 0.001**
Conditional R^2^: 0.966; Marginal R^2^: 0.461				

Significant values are in [bold].

### Prosthetic leg length change with RSP height adjustment

Every 1 cm increase in standing ProsL length of athletes with UTTA increased running leg length by 0.72 cm at touchdown, 0.75 cm at mid-stance, and 0.61 cm at take-off (Appendix 2, p < 0.001). For athletes with BTTA, every 1 cm increase in standing ProsL length increased running leg length by 0.88 cm at touchdown, 0.63 cm at mid-stance, and 0.71 cm at take-off (Appendix 2, p < 0.001).

## Discussion

We reject our first hypothesis because athletes with UTTA had shorter running ProsL than BioL lengths at mid-stance and take-off, even though standing ProsL lengths were 2.4 cm (2.6%) longer than BioL lengths. Based on our results (Appendix 1), to achieve equivalent running leg lengths across speeds at touchdown and take-off, athletes with UTTA should set their RSP height so that standing ProsL length is 2.8–4.5% longer than BioL length. Our findings support our second hypothesis because athletes with BTTA had ProsL L_ratio_ 3.0–3.4% less than BioL L_ratio_ in NA athletes at mid-stance and take-off. These results indicate that MASH regulations for athletes with BTTA do not result in equivalent running ProsL lengths compared to BioL lengths. Thus, to achieve equivalent running leg lengths at touchdown and take-off across speeds, athletes with BTTA should set their RSP height so that their standing ProsL lengths are 2.1–3.9% longer than their presumed standing BioL lengths.

Compared to running BioL lengths, ProsL lengths are shorter at mid-stance and take-off in athletes with UTTA. At mid-stance, a shorter ProsL than BioL could be due to differences between BioL and ProsL stiffness. A ProsL is 3.6–12% less stiff than a BioL in athletes with UTTA running at 5–6 m/s^1,13,14^, which yields greater ProsL compression per unit force than a BioL. A shorter ProsL length at take-off likely reflects the inability to actively plantarflex during running. ProsL L_ratio_ was less than BioL L_ratio_ in athletes with UTTA, and ProsL L_ratio_ was less in athletes with BTTA than BioL L_ratio_ in NA athletes, and the difference in L_ratio_ between legs increased for touchdown and takeoff at faster speed. The interaction between leg type and speed could be due to changes in foot-strike patterns with faster speeds. NA runners typically choose a rearfoot-strike pattern at slower speeds and transition to forefoot/midfoot-landing at faster speeds^15^. A forefoot- or midfoot- versus rearfoot-strike pattern has been associated with ~ 10° greater ankle joint plantarflexion at touchdown^15^, which would increase BioL length at touchdown.

Comparable running leg lengths between a ProsL and BioL can be achieved by increasing RSP stiffness or height. ProsL stiffness affects leg length at midstance, but not at touchdown or take-off. However, running with stiffer RSPs may increase injury risk and/or impair performance by increasing metabolic energy expenditure of athletes with BTTA^16^. Increasing RSP height could allow comparable running ProsL and BioL lengths. Our results show that for every 1 cm increase in RSP height in athletes with BTTA there was a 0.63–0.88 cm increase in running ProsL length during touchdown, mid-stance, and take-off. Increasing RSP height likely affects prosthetic energy storage and return since it results in decreased RSP stiffness and greater leg length displacement and center of mass displacement for a given ground reaction force during stance phase^17^. Additionally, a taller RSP has a greater moment of inertia, which would decrease step frequency at a given speed compared to a shorter RSP. Thus, there are potential trade-offs for increasing RSP height on performance.

There are no athletics regulations that limit the BioL lengths of NA athletes or the ProsL length of athletes with UTTA. However, athletes with BTTA must adjust their RSP height so that they do not exceed their presumed MASH to compete in sanctioned athletics events^9,10^. MASH estimates an athlete’s barefoot standing height based on body segment dimensions and population studies of NA^11^. On top of the uncertainty and potential bias in estimating an athlete’s standing height, running leg length is not universally proportional to standing leg length. The MASH rule forces athletes with BTTA to *run* with shorter leg lengths than presumed leg length based on their biological dimensions. Moreover, in sanctioned athletics events, NA athletes race wearing shoes with midsole heights of ≤ 2 cm^22^. Thus, to allow athletes with BTTA to compete with equivalent running leg lengths as non-amputee athletes, they should be allowed to increase their RSP height by 2.1–3.9% of their presumed standing BioL lengths based on running speed, and then add another 2.0 cm to account for footwear midsole height. For example, the current MASH rule estimates^9,23^ that the fastest ever 400 m athlete with BTTA would have BioL lengths of 0.944 m if they had not been amputated. Using average running speed from this athlete’s fastest 400 m performance (9.01 m/s) and adding 2.0 cm to account for footwear midsole height, our results suggest this athlete’s standing ProsL lengths should be 0.998 m to achieve equivalent running leg lengths as a NA athlete with standing BioL lengths of 0.944 m. In other words, this athlete would need to increase their standing ProsL lengths 5.4 cm beyond their MASH regulated ProsL lengths to attain similar running leg lengths as proportional NA athletes.

This study has potential limitations. First, we calculated leg length as the distance between the hip joint center and a marker on the distal end of the shoe or RSP. We used these markers rather than center of pressure data, which are noisy at touchdown and take-off. The use of metatarsal markers to measure leg length does not incorporate toe length. Toe plantarflexion may increase BioL length by up to ~ 7.0 cm at take-off^24^ and thus the difference between running BioL and ProsL lengths may be even greater. We used one RSP] model and future studies that assess other RSP configurations would inform RSP height prescription to achieve equivalent running ProsL and BioL lengths across speeds.

## Conclusions

We compared prosthetic and biological leg lengths during standing and during the stance phase of running. Overall, a ProsL is relatively shorter than a BioL during running for athletes with UTTA and BTTA. To achieve equivalent running leg lengths at touchdown and take-off, athletes with UTTA should set their RSP height so that standing ProsL length is at least 3.4% longer than BioL length and include footwear sole height (≤ 2.0 cm) across speeds. To achieve equivalent running leg lengths at touchdown and take-off, athletes with BTTA should set their RSP height so that standing ProsL lengths are at least 2.1% and 3.9% longer than desired BioL lengths and include footwear sole height across speeds. We encourage policymakers to consider task-specific biomechanics when setting athletics regulations. Until then, current regulations mandate that athletes with bilateral leg amputations run at shorter than presumed heights.

## Methods

### Participants

Eleven NA athletes, ten athletes with UTTA, and five athletes with BTTA participated (Table 3). Athletes with UTTA and BTTA had ≥ 1-year of experience using an RSP. Participants were all over 18 years old and reported that they were free of cardiovascular disease and musculoskeletal injuries beyond an amputation. The study protocol involving NA athletes was approved by the Intermountain Healthcare Urban Central Region Institutional Review Board, and the study protocol involving athletes with UTTA and BTTA was approved by the Colorado Multiple Institutional Review Board and USAMRMC Office of Research Protection Human Research Protection Office. Written informed consents were obtained from all participants.

**Table 3:** Participant characteristics (Mean ± SD) for non-amputee (NA) athletes, and athletes with unilateral (UTTA) and bilateral transtibial amputations (BTTA). Participant mass includes the RSP(s). We calculated standing prosthetic leg length when the leg was loaded with 0.5 body weight^17^.

Athlete cohort	Sex (F/M)	Age (yr)	Mass (kg)	Standing height (m)	Standing leg length (m)	
					Biological	Prosthetic
NA	3/8	20.6 ± 7.3	72.5 ± 10.8	1.77 ± 0.06	0.93 ± 0.04	N/A
UTTA	3/7	28.4 ± 5.5	76.0 ± 12.4	1.77 ± 0.12	0.93 ± 0.07	0.98 ± 0.07
BTTA	0/5	24.8 ± 4.8	71.5 ± 2.73	1.88 ± 0.04	N/A	1.03 ± 0.04

### Prosthesis setup

Each athlete with UTTA or BTTA was aligned with 1E90 Sprinter RSPs (Ottobock, Duderstadt, Germany) at the manufacturer recommended stiffness category by a certified prosthetist^4^. For athletes with UTTA, the prosthetist first used a tape measure to measure the BioL leg length as the distance from the greater trochanter to the floor during standing, and then set the recommended RSP height so that unloaded ProsL length, measured from the greater trochanter to the distal end of the unloaded RSP, was 2–8 cm longer than standing BioL length based on the athlete’s and prosthetist’s preference^6,17^. For athletes with BTTA, the prosthetist set the recommended RSP height so that standing height followed the 2014 IPC competition guideline maximum height (IPC_max_) for each athlete^25^. We were unable to match IPC_max_ for one athlete due to his relatively short residual limb lengths and the build height of the RSPs, so we set RSP height 3 cm shorter than IPC_max_ for this athlete and considered this height to be recommended.

### Experimental protocol

NA athletes completed one experimental session, and athletes with UTTA and BTTA completed a series of experimental sessions. During each experimental session, participants started a succession of constant speed trials on a force-measuring treadmill (Treadmetrix, Park City, UT) at 3 m/s. If the trial was successful, we increased speed by 1 m/s in each subsequent trial until the athlete approached their maximum speed, at which point we employed smaller speed increments. A trial was deemed successful if the participant maintained forward position on the treadmill for at least 8 consecutive strides^1,6,20^. If unsuccessful, participants could try again or deem their most recent successful speed as their maximum. Athletes were given ad libitum rest between trials.

Athletes with UTTA and BTTA ran using the 1E90 Sprinter RSP with three stiffness categories (manufacturer’s recommended category and ± 1) at the recommended RSP height and we identified the optimal category as the one that elicited the fastest maximum speed. Subsequently, athletes with UTTA and BTTA ran using the optimal stiffness category with RSP heights of ± 2 cm compared to their recommended RSP height. For the athlete who could not reach the IPC_max_, we only analyzed the RSP height of -2 cm compared to recommended RSP height. We randomized the trial order for RSP stiffness categories at recommended height, determined the optimal category, and then randomly inserted RSP height trials into the trial order. We analysed trials from 3 m/s to maximum speed when athletes used the optimal RSP stiffness category at recommended and ± 2 cm heights.

### Data collection and analysis

**Figure 3:**
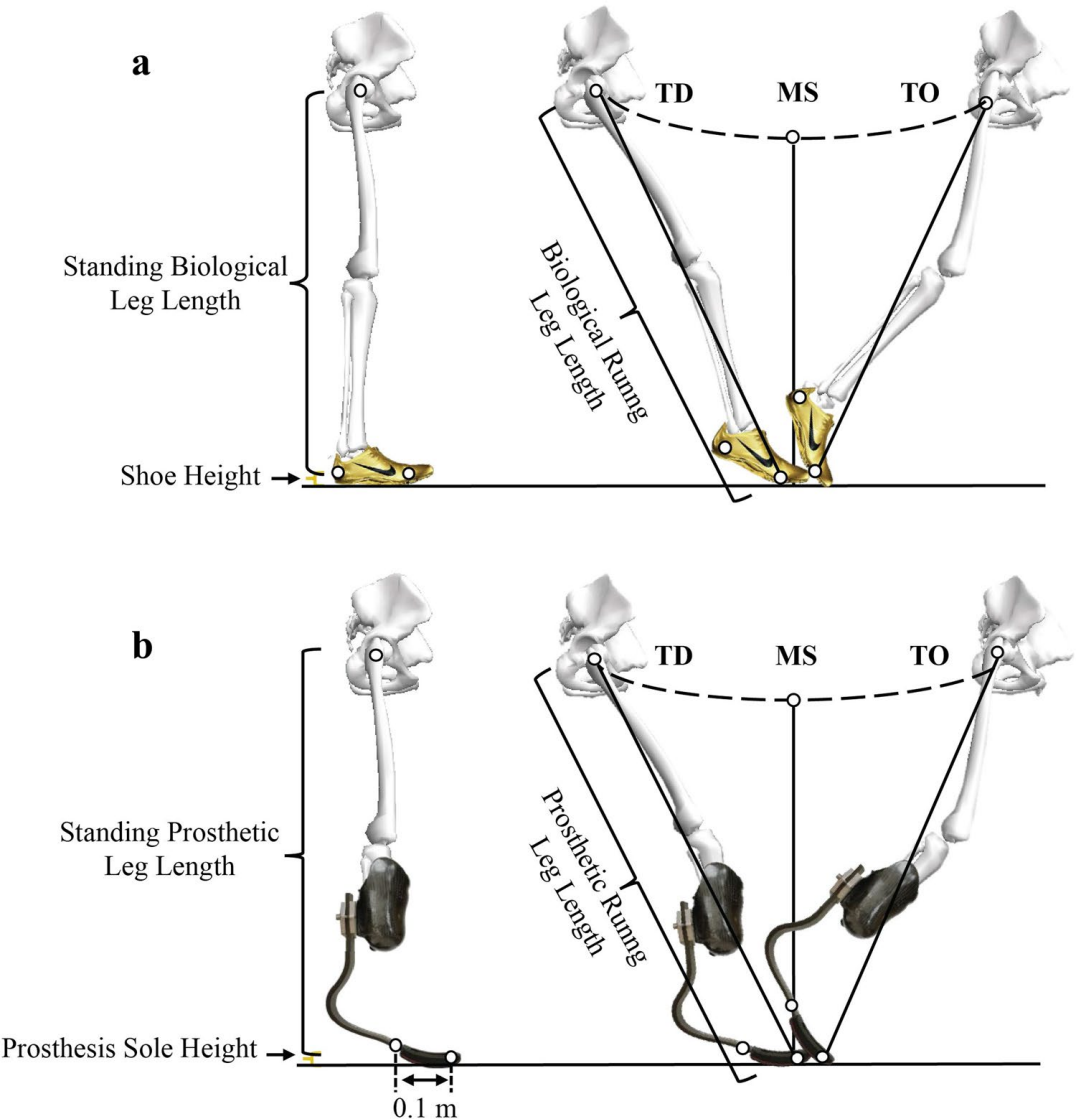
Illustration of leg lengths during standing and the stance phase of running for (**a**) biological legs and (**b**) prosthetic legs at touchdown (TD), mid-stance (MS), and take-off (TO). Leg length is the distance from the hip joint center to a marker on the shoe or prosthesis and does not include shoe sole height (**a**) or prosthesis sole height (**b**).

Prior to each experimental session, we placed reflective markers on the pelvis and a marker on the heel and first metatarsal head on the shoe for a BioL and on the distal end and 0.1 m posterior to the distal end on the RSP for a ProsL (Fig. 3). We simultaneously collected 3D marker trajectories (200 Hz) and vertical ground reaction forces (GRFs) (1000 Hz; Vicon, Oxford, UK) for standing and running trials. We labelled the markers from the standing trial and used these to auto-label the marker trajectories for the running trials (Vicon Nexus version 2.10). After auto-labelling, we verified the marker trajectories, and then exported these data to Visual 3D (C-Motion, Germantown, MD, USA). We used a CODA rigid segment model to estimate hip joint centers for both legs based on the pelvis markers^26^. We then used a custom written program in MATLAB (R2019b, MathWorks, Natick, MA) to calculate all leg length variables. We calculated BioL length as the vertical distance from the hip joint center to the average height of the markers on the shoe (Fig. 3a). We calculated unloaded ProsL length as the distance from the hip joint center to a marker placed on the distal end of the RSP (Fig. 3b). We subtracted the distance that the respective RSP compressed under half the participant’s body weight^17^ from the unloaded ProsL length to obtain standing ProsL length.

We filtered kinematic and kinetic data using a 4th-order low-pass Butterworth filter with a 30 Hz cutoff. We used a 50 N vertical GRF threshold to identify ground contact during running. We defined mid-stance as the timepoint halfway between touchdown and take-off. We analyzed at least 8 consecutive steps from each leg at each speed for each athlete.

We calculated running leg length using two landmarks, the hip joint center and most distal marker on the BioL shoe (Fig. 3a) or on the ProsL RSP (Fig. 3b). Leg length at touchdown and take-off were calculated as the distance between landmarks, and leg length at mid-stance was calculated as the vertical distance between landmarks. To compare relative leg length changes across athletes, we also calculated leg length ratio (L_ratio_, Appendix 1) as the quotient of running leg length at touchdown, mid-stance, and take-off to standing leg length.

### Statistical analyses

We compared standing ProsL and BioL lengths for athletes with UTTA using a paired t-test. We constructed linear mixed models^27^ to test for the effects of leg type (categorical; ProsL or BioL), speed (numerical; speed in m/s), and the interaction between leg type and speed on running leg length (m) and leg length ratio (L_ratio_) at touchdown, mid-stance, and take-off for athletes with UTTA. We constructed linear mixed models^27^ to test for the effects of leg type (categorical; BioL or ProsL), speed (numerical; speed in m/s), and the interaction between leg type and speed on L_ratio_ at touchdown, mid-stance, and toe-off for NA athletes and athletes with BTTA. First, we included all independent variables as factors (leg type and speed) and their interactions to determine factors that had a significant main effect, and then simplified the model using a step-down model-building approach until only statistically significant factors and interactions were present^28^. We then obtained equations to determine the standing ProsL length that elicited an equivalent running BioL length (Appendix 1). We also constructed linear mixed models to test for the effects of standing ProsL length, speed, and the interaction between standing ProsL length and speed on running ProsL length at touchdown, mid-stance, and take-off for athletes with UTTA and BTTA to determine if ± 2 cm changes in RSP height directly translated to changes in running ProsL length. We used a significance level of p < 0.05. All statistical tests were done in RStudio (Boston, MA, USA)^29^.

### Ethical approval

The study protocol involving NA athletes was approved by the Intermountain Healthcare Urban Central Region Institutional Review Board, and the study protocol involving athletes with unilateral and bilateral amputations was approved by the Colorado Multiple Institutional Review Board (COMIRB #13–2315) and USAMRMC Office of Research Protection Human Research Protection Office. Written informed consents were obtained from all participants. All authors confirmed that all experiments were performed in accordance with relevant guidelines and regulations.

## Supplementary Information

Supplementary Information 1.

Supplementary Information 2.

Supplementary Information 3.

## Data Availability

The dataset generated and analyzed during the current study are uploaded in supplementary materials. We provided a spreadsheet with running leg length (long format).

## References

[1] McGowan CP, Grabowski AM, McDermott WJ (2012). Leg stiffness of sprinters using running-specific prostheses. J. R. Soc. Interface.

[2] Alt T, Heinrich K, Funken J (2015). Lower extremity kinematics of athletics curve sprinting. J. Sports Sci..

[3] Kuitunen S, Komi PV, KyöLäinen H (2002). Knee and ankle joint stiffness in sprint running. Med. Sci. Sports Exerc..

[4] Ottobock Fitting Guide for TT Sports Prosthesis. 2014.

[5] Lechler K, Lilja M (2008). Lower extremity leg amputation: an advantage in running?. Sports Technol..

[6] Grabowski AM, McGowan CP, McDermott WJ (2010). Running-specific prostheses limit ground-force during sprinting. Biol. Lett..

[7] Baum BS, Hobara H, Kim YH (2016). Amputee locomotion: Ground reaction forces during submaximal running with running-specific prostheses. J. Appl. Biomech..

[8] Strike SC, Arcone D, Orendurff M (2018). Running at submaximal speeds, the role of the intact and prosthetic limbs for trans-tibial amputees. Gait Posture.

[9] World Para Athletics Classification Rules and Regulations - Appendix 1, art. 3.1.4.3. 2017.https://www.paralympic.org/

[10] International Paralympic Committee. IPC Athletics Classification Rules and Regulations. 2013.

[11] Canda A (2009). Stature estimation from body segment lengths in young adults—Application to people with physical disabilities. J. Physiol. Anthropol..

[12] Connick MJ, Beckman E, Ibusuki T (2016). Evaluation of methods for calculating maximum allowable standing height in amputees competing in Paralympic athletics. Scand. J. Med. Sci. Sports.

[13] Sano Y, Makimoto A, Hashizume S (2017). Leg stiffness during sprinting in transfemoral amputees with running-specific prosthesis. Gait Posture.

[14] Hobara H, Sakata H, Hashizume S (2019). Leg stiffness in unilateral transfemoral amputees across a range of running speeds. J. Biomech..

[15] Breine B, Malcolm P, Galle S (2019). Running speed-induced changes in foot contact pattern influence impact loading rate. Eur. J. Sport Sci..

[16] Beck ON, Taboga P, Grabowski AM (2017). Reduced prosthetic stiffness lowers the metabolic cost of running for athletes with bilateral transtibial amputations. J. Appl. Physiol..

[17] Beck ON, Taboga P, Grabowski AM (2016). Characterizing the Mechanical Properties of Running-Specific Prostheses. PLoS ONE.

[18] Beck ON, Taboga P, Grabowski AM (2017). How do prosthetic stiffness, height and running speed affect the biomechanics of athletes with bilateral transtibial amputations?. J. R. Soc. Interface.

[19] Kulmala J-P, Avela J, Pasanen K (2013). Forefoot strikers exhibit lower running-induced knee loading than rearfoot strikers. Med. Sci. Sports Exerc..

[20] Beck ON, Grabowski AM (2018). Athletes with versus without leg amputations: Different biomechanics, similar running economy. Exercise Sport Sci. Rev..

[21] Taboga P, Drees EK, Beck ON (2020). Prosthetic model, but not stiffness or height, affects maximum running velocity in athletes with unilateral transtibial amputations. Sci. Rep..

[22] World Athletics. Rules and Regulations of World Athletics (2021).

[23] Beck ON, Taboga P, Grabowski AM (2022). Sprinting with prosthetic versus biological legs: insight from experimental data. Royal Society Open Science.

[24] Rolian C, Lieberman DE, Hamill J (2009). Walking, running and the evolution of short toes in humans. J. Exp. Biol..

[25] International Paralympic Committee. IPC Athletics Classification Rules and Regulations. 2014.

[26] Bell AL, Brand RA, Pedersen DR (1989). Prediction of hip joint centre location from external landmarks. Hum. Mov. Sci..

[27] Bates D, Kliegl R, Vasishth S, *et al.* Parsimonious Mixed Models. arXiv:150604967 [stat] Published Online First: June 2015.http://arxiv.org/abs/1506.04967. Accessed 18 Jan 2022.

[28] Kuznetsova A, Brockhoff PB, Christensen RHB (2017). lmerTest package: Tests in linear mixed effects models. J. Stat. Softw..

[29] Bates D, Mächler M, Bolker B, *et al.* Fitting Linear Mixed-Effects Models using lme4. arXiv:14065823 [stat] Published Online First: 23 June 2014.http://arxiv.org/abs/1406.5823 (accessed 12 Mar 2022).

