# Author Correction: Equivalent running leg lengths require prosthetic legs to be longer than biological legs during standing

**DOI:** 10.1038/s41598-023-36392-x

**Published:** 2023-06-06

**Authors:** Janet H. Zhang-Lea, Joshua R. Tacca, Owen N. Beck, Paolo Taboga, Alena M. Grabowski

**Affiliations:** 1 Department of Integrative Physiology, University of Colorado Boulder, Boulder, CO USA; 2 Department of Human Physiology, Gonzaga University, Spokane, WA USA; 3 Paul M. Rady Department of Mechanical Engineering, University of Colorado Boulder, Boulder, CO USA; 4 Department of Kinesiology and Health Education, University of Texas at Austin, Austin, TX USA; 5 Department of Kinesiology, Sacramento State University, Sacramento, CA USA; 6 Department of Veterans Affairs, Eastern Colorado Healthcare System, Denver, CO USA

Correction to: *Scientific Reports* 10.1038/s41598-023-34346-x, published online 11 May 2023

The original version of this Article contained an error in Affiliation 2, which was incorrectly given as ‘Department of Human Physiology, Gonzaga University, Spokane, USA’. The correct affiliation is listed below:

Department of Human Physiology, Gonzaga University, Spokane, WA, USA

The original Article has been corrected.

