# Supplementary material for: Equivalent running leg lengths require prosthetic legs to be longer than biological legs during standing
Source: Sci Rep. 2023 May 11;13:7679. doi: 10.1038/s41598-023-34346-x (PMC10175537; doi:10.1038/s41598-023-34346-x)
Supplement: Supplementary file 1 — Supplementary Information 1. [file 41598_2023_34346_MOESM1_ESM.docx]

**Appendix**-**1**

**Methods (cont.)**

We calculated leg length ratio (L_ratio_) as the quotient of running and standing leg length (Eqn. 1).

$$L_{ratio}=\frac{Running Leg Length}{Standing Leg Length} \left( 1 \right).$$

We analyzed three discrete time points (touchdown, mid-stance, and take-off) during the stance phase of running and constructed linear mixed models to predict L_ratio_ as a function of leg type, speed, and the interaction for athletes with unilateral transtibial amputation (Eqn. 2).

$$\frac{Running Leg Length}{Standing Leg Length}=(A\times Leg Type)+(B\times Speed)+(C\times Leg Type\times Speed)+D \left( 2 \right).$$

Leg type was defined as a categorical variable that equals 1 for a prosthetic leg (ProsL) and 0 for a biological leg (BioL). Therefore, we applied leg type to Eqn. 2 for a ProsL (Eqn. 3) and a BioL (Eqn. 4).

$$\frac{Running ProsL Length}{Standing ProsL Length}=A+(\left( B+C \right)\times Speed)+D \left( 3 \right),$$

$$\frac{Running BioL Length}{Standing BioL Length}=(B\times Speed)+D \left( 4 \right).$$

To determine the standing ProsL length needed for equivalent running leg lengths between a BioL and ProsL, we set running ProsL length equal to running BioL length (Eqn. 5).

$\left[ A+(\left( B+C \right)\times Speed)+D \right]\times Standing ProsL Length=[(B\times Speed)+D]\times Standing BioL Length$ (5).

We solved Eqn. 5 for standing ProsL length. Then, we calculated standing ProsL length as a function of standing BioL length for equivalent running leg lengths (Eqn. 6).

$$Standing ProsL Length=\frac{[(B\times Speed)+D] \times Standing BioL Length}{\left[ A+(\left( B+C \right)\times Speed)+D \right]} \text{(}\text{6}\text{)} .$$

The presumed the standing ProsL length (Eqn. 6) needed to achieve equivalent running leg length depended on running speed. To provide an overall equation that allows athletes to have equivalent running leg lengths throughout stance phase, we solved Eqn. 6 by imposing a standing BioL length of 1 m and calculated the respective standing ProsL length at speeds ranging from 3 to 11.5 m/s. We obtained two estimated values for standing ProsL length at each speed from 3 to 11.5 m/s, one for touchdown and one for take-off. Since leg length at mid-stance is affected by RSP stiffness, we did not use mid-stance to estimate standing ProsL length. We report the range of average standing ProsL lengths needed for equivalent running leg lengths at touchdown and take-off by calculating the average of touchdown and take-off standing ProsL length at each speed (3-11.5 m/s).

**Appendix**-**2**

**Results (cont.) Change in RSP height by ± 2 cm**

Athletes with UTTA

We changed the RSP height of the ProsL by ±2 cm for athletes with unilateral transtibial amputation (UTTA) and found that every 1 cm increase in standing ProsL length increased running ProsL length by 0.72 cm at touchdown (p<0.001; Supplementary Table 1; Supplementary Figure 1a), 0.75 cm at midstance (p<0.001; Supplementary Table 1, Supplementary Figure 1c), and 0.73 cm at take-off (p< 0.001; Supplementary Table 1; Supplementary Figure 1e). We found an interaction between speed and standing ProsL length on running ProsL length at touchdown (p<0.001; Supplementary Table 1; Supplementary Figure 1), where increasing RSP height by 1 cm would increase running ProsL length by 0.72 cm at 3 m/s and by 0.49 cm at 11.5 m/s.

Athletes with BTTA

We changed the RSP height of the ProsL by ±2 cm for athletes with bilateral transtibial amputations (BTTA) and found that every 1 cm increase in standing ProsL length increased running ProsL length by 0.88 cm at touchdown (p<0.001; Supplementary Table 2; Supplementary Figure 1b), 0.63 cm at midstance (p<0.001; Supplementary Table 2, Supplementary Figure 1d), and 0.71 cm at take-off (p<0.001; Supplementary Table 2; Supplementary Figure 1f). We found an interaction between speed and standing ProsL length on running ProsL length at touchdown and take-off, where increasing RSP height by 1 cm increased running ProsL length by 0.88 cm at touchdown and 0.71 cm at take-off at 3 m/s and by 0.51 cm at touchdown and 0.88 cm at take-off at 10 m/s (Supplementary Table 2, Supplementary Figure 1).

**Supplementary Table 1.** Linear mixed model parameters for fixed effects of standing prosthetic leg length (m), speed (m/s), and speed interaction on running prosthetic leg length (m) at touchdown, midstance, and take-off for athletes with unilateral transtibial amputation. Coefficient estimates, coefficient standard errors (*SE*), t values (*t*), and p values (*p*) are listed. Subject was set as a random effect.

| **Touchdown Leg Length (m)** | ***Estimate (B)*** | ***SE*** | ***t*** | ***p*** |
| --- | --- | --- | --- | --- |
| Intercept | 0.313 | 0.062 | 5.03 | **<0.001** |
| Speed [m/s] | 0.019 | 0.005 | 4.00 | **<0.001** |
| Standing Leg Length [m] | 0.721 | 0.063 | 11.49 | **<0.001** |
| Speed [m/s]*Standing Leg Length [m] | -0.020 | 0.005 | -4.24 | **<0.001** |
| Conditional R^2^: 0.954; Marginal R^2^: 0.704 | | | | |
| **Midstance Leg Length (m)** | ***Estimate (B)*** | ***SE*** | ***t*** | ***p*** |
| Intercept | 0.147 | 0.051 | 2.89 | **0.005** |
| Standing Leg Length [m] | 0.751 | 0.051 | 14.69 | **<0.001** |
| Conditional R^2^: 0.973; Marginal R^2^: 0.802 | | | | |
| **Toe-off Leg Length (m)** | ***Estimate (B)*** | ***SE*** | ***t*** | ***p*** |
| Intercept | 0.411 | 0.053 | 7.70 | **<0.001** |
| Speed [m/s] | 0.002 | 0.001 | 3.93 | **<0.001** |
| Standing Leg Length [m] | 0.607 | 0.054 | 11.21 | **<0.001** |
| Conditional R^2^: 0.950; Marginal R^2^: 0.777 | | | | |

**Supplementary Table 2.** Linear mixed model parameters for fixed effects of standing leg length (m), speed (m/s), and speed interaction on running leg length (m) at touchdown, midstance, and take-off for athletes with bilateral transtibial amputations. Coefficient estimates, coefficient standard errors (*SE*), t values (*t*), and p values (*p*) are listed. Subject was set as a random effect.

| **Touchdown Leg Length (m)** | ***Estimate (B)*** | ***SE*** | ***t*** | ***p*** |
| --- | --- | --- | --- | --- |
| Intercept | 0.139 | 0.105 | 1.33 | 0.19 |
| Speed [m/s] | 0.037 | 0.011 | 3.31 | **0.001** |
| Standing Leg Length [m] | 0.882 | 0.100 | 8.85 | **<0.001** |
| Speed [m/s]*Standing Leg Length [m] | -0.037 | 0.011 | -3.54 | **<0.001** |
| Conditional R^2^: 0.931; Marginal R^2^: 0.611 | | | | |
| **Midstance Leg Length (m)** | ***Estimate (B)*** | ***SE*** | ***t*** | ***p*** |
| Intercept | 0.250 | 0.071 | 3.53 | **<0.001** |
| Standing Leg Length [m] | 0.630 | 0.067 | 9.38 | **<0.001** |
| Conditional R^2^: 0.936; Marginal R^2^: 0.650 | | | | |
| **Toe-off Leg Length (m)** | ***Estimate (B)*** | ***SE*** | ***t*** | ***p*** |
| Intercept | 0.306 | 0.062 | 4.91 | **<0.001** |
| Speed [m/s] | -0.018 | 0.006 | -2.78 | **0.007** |
| Standing Leg Length [m] | 0.714 | 0.059 | 12.15 | **<0.001** |
| Speed [m/s]*Standing Leg Length [m] | 0.017 | 0.006 | 2.73 | **0.008** |
| Conditional R^2^: 0.981; Marginal R^2^: 0.777 | | | | |

**Supplementary Figure 1.** Running versus standing prosthetic leg length (m) at touchdown (a,b), mid-stance (c,d), and take-off (e,f) for athletes with unilateral transtibial amputation (left column) and bilateral transtibial amputations (right column). Different colors refer to different athletes. The colored lines are linear lines of best fit between the running and standing prosthetic leg length for each athlete. The black line is the line of identity where running prosthetic leg length equals standing prosthetic leg length.
