# Supplementary material for: Equivalent running leg lengths require prosthetic legs to be longer than biological legs during standing
Source: Sci Rep. 2023 May 11;13:7679. doi: 10.1038/s41598-023-34346-x (PMC10175537; doi:10.1038/s41598-023-34346-x)
Supplement: Supplementary file 2 — Supplementary Information 2. [file 41598_2023_34346_MOESM2_ESM.zip › Supplemental Materials/Plots.pdf]

Plots for 'Natural prosthetic leg lengths during standing yield unnaturally short legs during running'

Janet H. Zhang-Lea, Joshua R. Tacca, Owen N. Beck, Paolo Taboga, Alena M. Grabowski

04/03/2022

Load and Organize RSP Height Data

```
# Load average data
rsp_height_data <- read.csv("Data.csv")

#Set subject ID as factor and create a column so that sub 1 from UL group is not same as sub 1 in NA gr
rsp_height_data$SubID <- as.factor(rsp_height_data$SubID)
rsp_height_data$SubAmp <- paste(rsp_height_data$SubID, rsp_height_data$AmpType)

#Bilateral subject 4 did not complete a trial at the recommended height, use the shortest height (-3cm)
rsp_height_data$Height_adj <- ifelse(rsp_height_data$SubAmp == '4 BL', rsp_height_data$Height + 3, rsp_

#Add +- 2cm to standing for prosthetic legs for non-recommended height trials (standing leg length repo
rsp_height_data$Standing_adj <- ifelse(rsp_height_data$Leg.Type == 'PL', rsp_height_data$standing_leg_l

#calculate leg length ratio (*100 to set it in terms of percentage)
rsp_height_data$ratio_td <- rsp_height_data$length_td/rsp_height_data$Standing_adj
rsp_height_data$ratio_ms <- rsp_height_data$length_ms/rsp_height_data$Standing_adj
rsp_height_data$ratio_to <- rsp_height_data$length_to/rsp_height_data$Standing_adj

#bilateral data
bilateral_data <- rsp_height_data[rsp_height_data$AmpType == "BL",]
bilateral_data.ht0 <- bilateral_data[bilateral_data$Height_adj == 0,]

#unilateral data
unilateral_data <- rsp_height_data[rsp_height_data$AmpType == "UL",]
unilateral_data.ht0 <- unilateral_data[unilateral_data$Height_adj == 0,]
unilateral_data.pl <- unilateral_data[unilateral_data$Leg.Type == "PL",]
unilateral_data.bioleg <- unilateral_data[unilateral_data$Leg.Type == "BioLeg",]

#non-amputee data
nonamputee_data <- rsp_height_data[rsp_height_data$AmpType == "NonAmp",]

#all data with just recommended height
rsp_height_data.ht0 <- rbind(bilateral_data.ht0, unilateral_data.ht0, nonamputee_data)

#combine non-amputee and bilateral data
bilatnonamp <- rbind(bilateral_data, nonamputee_data)
bilatnonamp.ht0 <- rbind(bilateral_data.ht0, nonamputee_data)

#data for standing leg length (NEED TO ADD STANDING COLUMN MANUALLY IN CSV FILE)
```

```
standing_data = rsp_height_data[rsp_height_data$Standing == 1,]
standing_data_ul = standing_data[standing_data$AmpType == "UL",]
standing_data_bl = standing_data[standing_data$AmpType == "BL",]
standing_data_nonamp = standing_data[standing_data$AmpType == "NonAmp",]
standing_data_bilatnonamp = rbind(standing_data_bl, standing_data_nonamp)
```

Prepare RSP Height Data

```
#Prepare data for the graphs

#Set widths for x-axis offsets in the graph between groups
width1 <- 0.2
width2 <- 0.15

#add a column called speed_shift to manually dodge AL from UL with a set width
unilateral_data.ht0$Speed_shift <- unilateral_data.ht0$Speed + width2*if_else(unilateral_data.ht0$Leg.Ty
bilateral_data.ht0$Speed_shift <- bilateral_data.ht0$Speed + width2*if_else(bilateral_data.ht0$Leg.Type
nonamputee_data$Speed_shift <- nonamputee_data$Speed + width2*if_else(nonamputee_data$Leg.Type == "PL",

#add standing_shift column
unilateral_data.ht0$Stand_shift <- 2 + width1*if_else(unilateral_data.ht0$Leg.Type == "PL", 1, -1)
bilateral_data.ht0$Stand_shift <- 2 + width1*if_else(bilateral_data.ht0$Leg.Type == "PL", 1, -1)
nonamputee_data$Stand_shift <- 2 + width1*if_else(nonamputee_data$Leg.Type == "PL", 1, -1)

#paste Speed and Subject into new column
unilateral_data.ht0$SpeedSub <- paste(unilateral_data.ht0$SubID,unilateral_data.ht0$Speed)
standing_data$SpeedSub <- paste(standing_data$SubID,standing_data$Speed)
bilateral_data.ht0$SpeedSub <- paste(bilateral_data.ht0$SubID,bilateral_data.ht0$Speed)
nonamputee_data$SpeedSub <- paste(nonamputee_data$SubID,nonamputee_data$Speed)

#Add a leg type and speed column
unilateral_data.ht0$LegSpeed <- paste(unilateral_data.ht0$Leg.Type,unilateral_data.ht0$Speed)
standing_data$LegSpeed <- paste(standing_data$Leg.Type,standing_data$Speed)
bilateral_data.ht0$LegSpeed <- paste(bilateral_data.ht0$Leg.Type,bilateral_data.ht0$Speed)
nonamputee_data$LegSpeed <- paste(nonamputee_data$Leg.Type,nonamputee_data$Speed)

#Leg speed amp type column
unilateral_data.ht0$LegSpeedAmp <- paste(unilateral_data.ht0$Leg.Type,unilateral_data.ht0$Speed,unilate
bilateral_data.ht0$LegSpeedAmp <- paste(bilateral_data.ht0$Leg.Type,bilateral_data.ht0$Speed,bilateral_
nonamputee_data$LegSpeedAmp <- paste(nonamputee_data$Leg.Type,nonamputee_data$Speed,nonamputee_data$Amp
standing_data$LegSpeedAmp <-  paste(standing_data$Leg.Type,standing_data$Speed,standing_data$AmpType)

#Speed sub amp type column
unilateral_data.ht0$SubSpeedAmp <- paste(unilateral_data.ht0$SubID,unilateral_data.ht0$Speed,unilateral
bilateral_data.ht0$SubSpeedAmp <- paste(bilateral_data.ht0$SubID,bilateral_data.ht0$Speed,bilateral_dat
nonamputee_data$SubSpeedAmp <- paste(nonamputee_data$SubID,nonamputee_data$Speed,nonamputee_data$AmpTyp
standing_data$SubSpeedAmp <- paste(standing_data$SubID,standing_data$Speed,standing_data$AmpType)

#combine into one data frame
rsp_height_data.ht0_all <- rbind(bilateral_data.ht0, unilateral_data.ht0, nonamputee_data)
rsp_height_data.ht0_blna <- rbind(bilateral_data.ht0, nonamputee_data)

#Split standing data by amputation type
standing_data.ul <- standing_data[standing_data$AmpType == "UL",]
```

```
standing_data.bl <- standing_data[standing_data$AmpType == "BL",]
standing_data.na <- standing_data[standing_data$AmpType == "NonAmp",]

#add stand shift column
standing_data.ul$Stand_shift <- 2 + width1*if_else(standing_data.ul$Leg.Type == "PL", 1, -1)
standing_data.bl$Stand_shift <- 2 + width1*if_else(standing_data.bl$Leg.Type == "PL", 2, -2)
standing_data.na$Stand_shift <- 2 + width1*if_else(standing_data.na$Leg.Type == "PL", 2, -2)

#Standing data for just bilaterals and non-amputees
standing_data.blna <- rbind(standing_data.bl, standing_data.na)

#only wholes number speeds & 11.5 m/s
unilateral_data.ht0_whole <- unilateral_data.ht0[!(unilateral_data.ht0$Speed %% 1) | unilateral_data.ht(

#only wholes number speeds
rsp_height_data.ht0_blna_whole <- rsp_height_data.ht0_blna[!(rsp_height_data.ht0_blna$Speed %% 1),]
```

Touchdown Plot (ADD LME)

```
#Standing Leg Length Graph
ex_st3 <- ggplot(data = standing_data.ul, aes(x=Stand_shift, y=standing_leg_length, fill = Leg.Type, sha
  scale_fill_manual(values = c("black", "white"))+
  scale_shape_manual(values = c(19, 21))+
  scale_color_manual(values = c("gray60", "gray60","gray60", "gray60","gray60", "gray60","gray60", "gra
  geom_line(aes(group = SpeedSub))+
  geom_point(size = 1, color = "gray60")+
  stat_summary(geom="errorbar", fun.data = mean_se, aes(group = Leg.Type)) +
  stat_summary(geom="point", fun = mean, size = 2.5, aes(group = Leg.Type))+
  scale_x_continuous(limits = c(1,3), breaks = 2, labels = "Standing", expand = c(0,0))+
  scale_y_continuous(limits = c(0.75,1.15))+
  labs(y = "Leg Length (m)")+
  theme_classic()+
  theme(legend.position = "none", plot.title = element_text(hjust = 0.5))

ex_st3
```

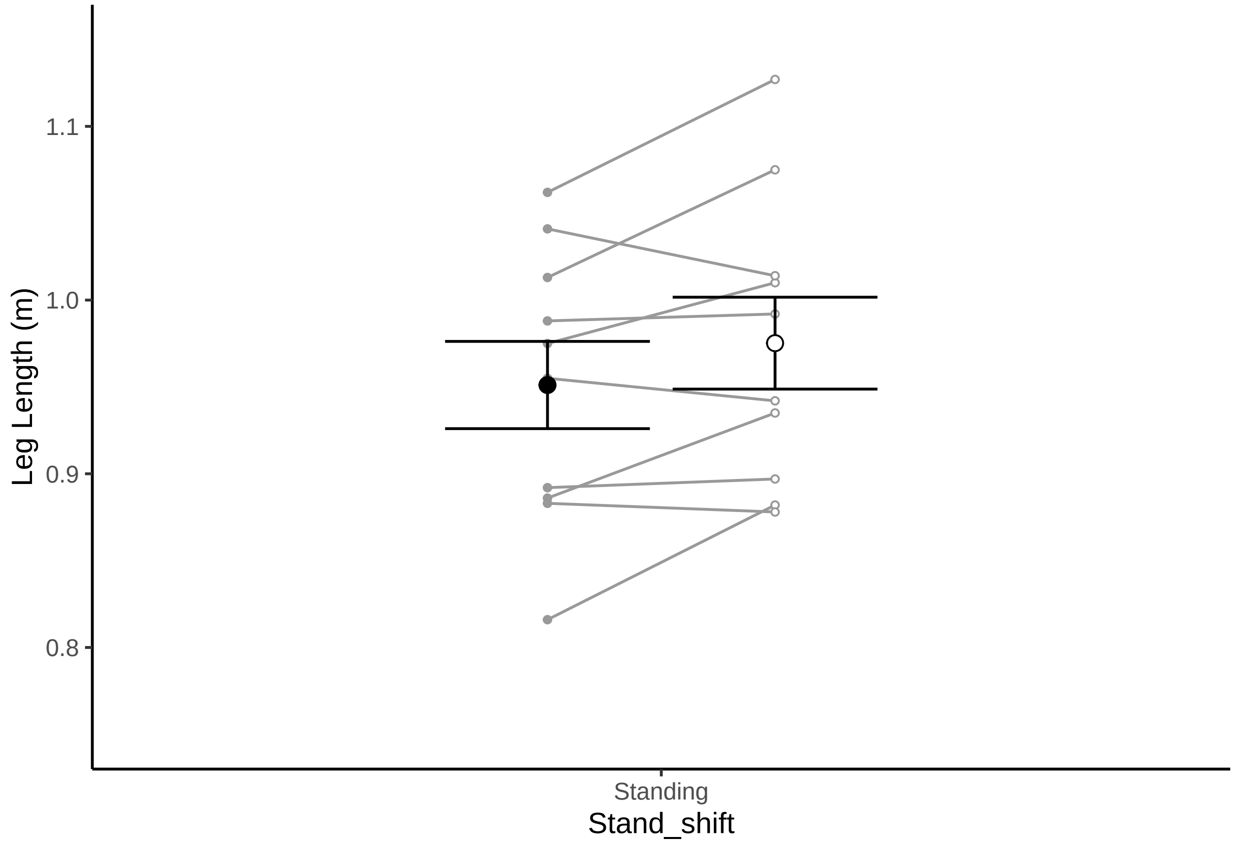


```
#LME from Stats.Rmd
LegLengthTD <- lmer(length_td ~ Speed  + Leg.Type + Leg.Type*Speed + (1|SubID), data=unilateral_data.ht

#Fixed effects from the model
LegLengthTD_effects <- as.data.frame(fixef(LegLengthTD))
LegLengthTD_int <- LegLengthTD_effects[1,1]
LegLengthTD_speed <- LegLengthTD_effects[2,1]
LegLengthTD_type_pl <- LegLengthTD_effects[3,1]
LegLengthTD_speed_type_pl <- LegLengthTD_effects[4,1]

#Touchdown Graph
ex_td4 <- ggplot(data = unilateral_data.ht0_whole, aes(x=Speed_shift, y=length_td, fill = Leg.Type, sha
  scale_fill_manual(values = c("black", "white"))+
  scale_shape_manual(values = c(19, 21))+
  scale_color_manual(values = c("gray60", "gray60","gray60", "gray60","gray60", "gray60","gray60", "gra
  geom_abline(slope = LegLengthTD_speed, intercept = LegLengthTD_int)+ #biological leg
  geom_abline(slope = LegLengthTD_speed + LegLengthTD_speed_type_pl, intercept = LegLengthTD_int + LegL
  geom_line(aes(group = SpeedSub, col = SubID))+
  geom_point(size = 1, color = "gray60")+
  stat_summary(geom="errorbar", fun.data = mean_se, aes(group = LegSpeed)) +
  stat_summary(geom="point", fun = mean, size = 2.5, aes(group = LegSpeed))+
  scale_x_continuous(breaks = c(3,4,5,6,7,8,9,10,11,12))+
  scale_y_continuous(limits = c(0.75,1.15))+
  labs(x = "Speed (m/s)", y = "Leg Length (m)")+
  theme_classic()+
  theme(legend.position = "none", plot.title = element_text(hjust = 0.5))
```

```
ex_td4
```

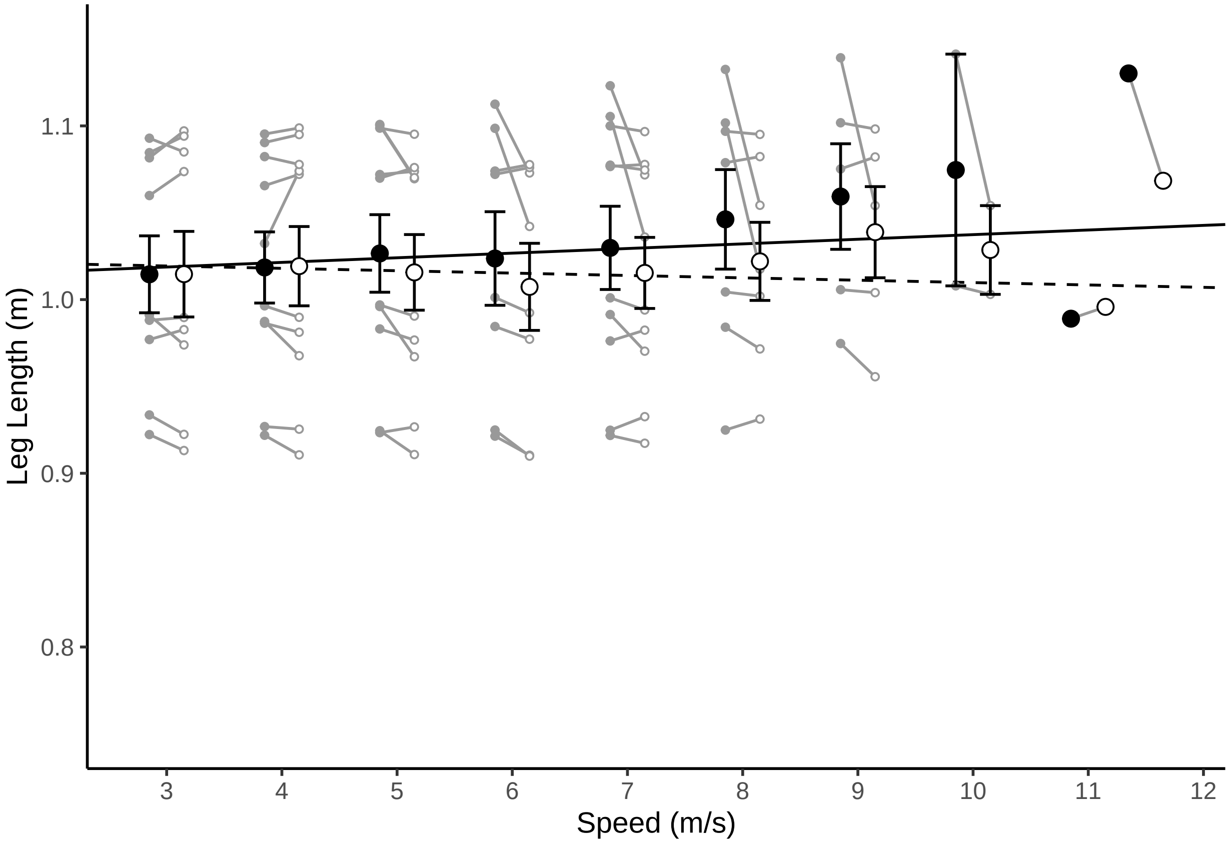


```
#Combine standing and touchdown graphs
ex_st_td4 <- plot_grid(ex_st3+theme(axis.title.x=element_blank()), ex_td4+theme(axis.line.y = element_bl
ex_st_td4
```

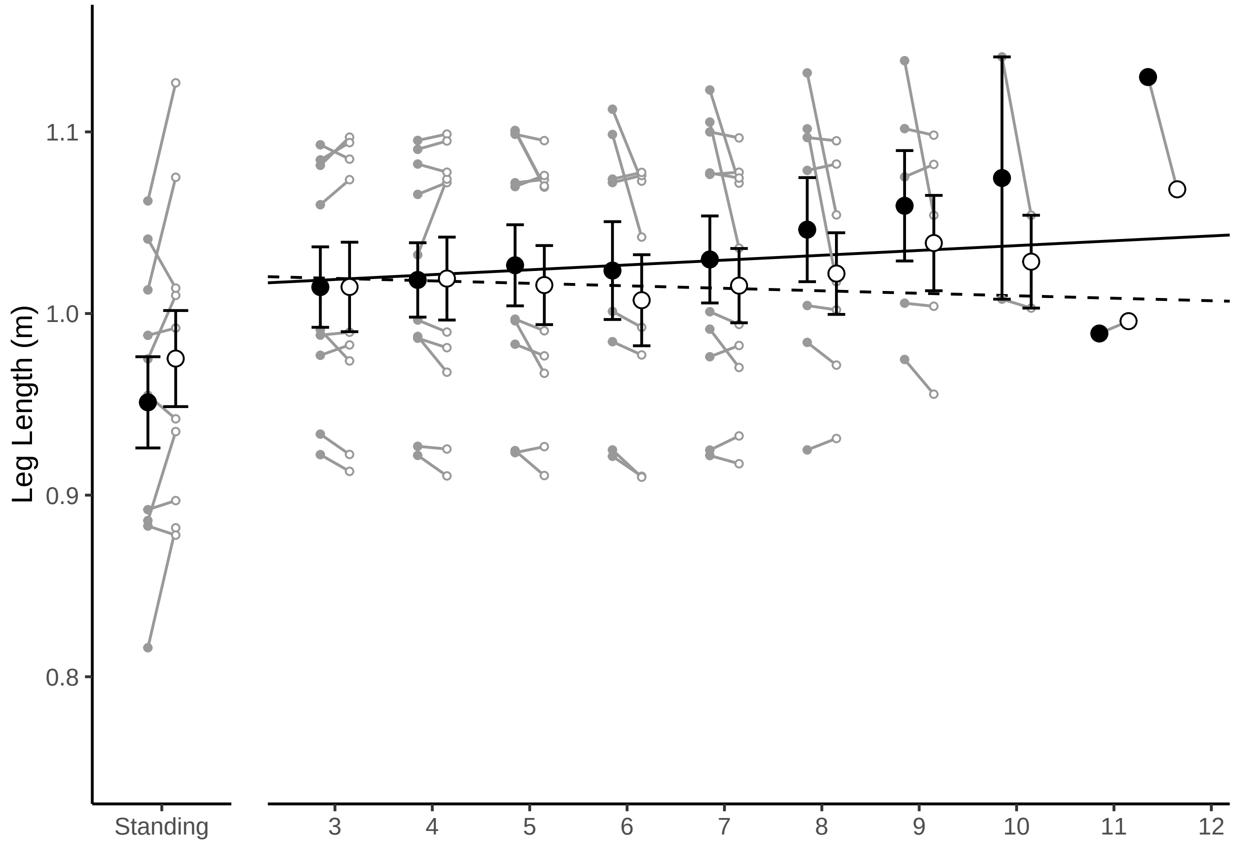

Midstance Plot (ADD LME)

```
#LME from Stats.Rmd
LegLengthMS<- lmer(length_ms ~ Speed  + Leg.Type + Leg.Type*Speed + (1|SubID), data=unilateral_data.ht0

#Fixed effects from the model
LegLengthMS_effects <- as.data.frame(fixef(LegLengthMS))
LegLengthMS_int <- LegLengthMS_effects[1,1]
LegLengthMS_speed <- LegLengthMS_effects[2,1]
LegLengthMS_type_pl <- LegLengthMS_effects[3,1]
LegLengthMS_speed_type_pl <- LegLengthMS_effects[4,1]

#Midstance Graph
ex_ms4 <- ggplot(data = unilateral_data.ht0_whole, aes(x=Speed_shift, y=length_ms, fill = Leg.Type, sha
  scale_fill_manual(values = c("black", "white"))+
  scale_shape_manual(values = c(19, 21))+
  scale_color_manual(values = c("gray60", "gray60","gray60", "gray60","gray60", "gray60","gray60", "gra
  geom_abline(slope = LegLengthMS_speed, intercept = LegLengthMS_int)+ #biological leg
  geom_abline(slope = LegLengthMS_speed + LegLengthMS_speed_type_pl, intercept = LegLengthMS_int + LegL
  geom_line(aes(group = SpeedSub, col = SubID))+
  geom_point(size = 1, color = "gray60")+
  stat_summary(geom="errorbar", fun.data = mean_se, aes(group = LegSpeed)) +
  stat_summary(geom="point", fun = mean, size = 2.5, aes(group = LegSpeed))+
  scale_x_continuous(breaks = c(3,4,5,6,7,8,9,10,11,12))+
  scale_y_continuous(limits = c(0.75,1.15))+
  labs(x = "Speed (m/s)", y = "Leg Length (m)")+
  theme_classic()+
  theme(legend.position = "none", plot.title = element_text(hjust = 0.5))
```

```
ex_ms4
```

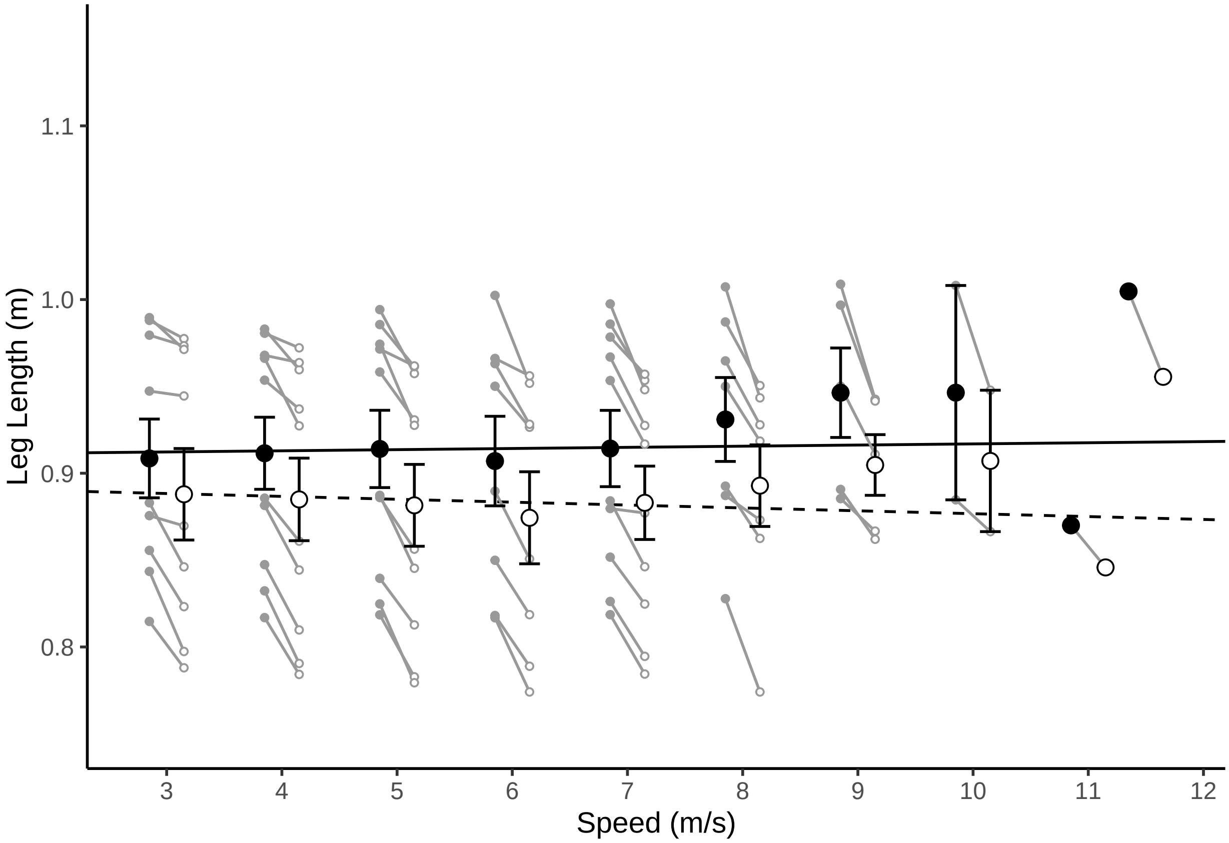


```
#Combine standing and midstance graphs
ex_st_ms4 <- plot_grid(ex_st3+theme(axis.title.x=element_blank()), ex_ms4+theme(axis.line.y = element_bl
ex_st_ms4
```

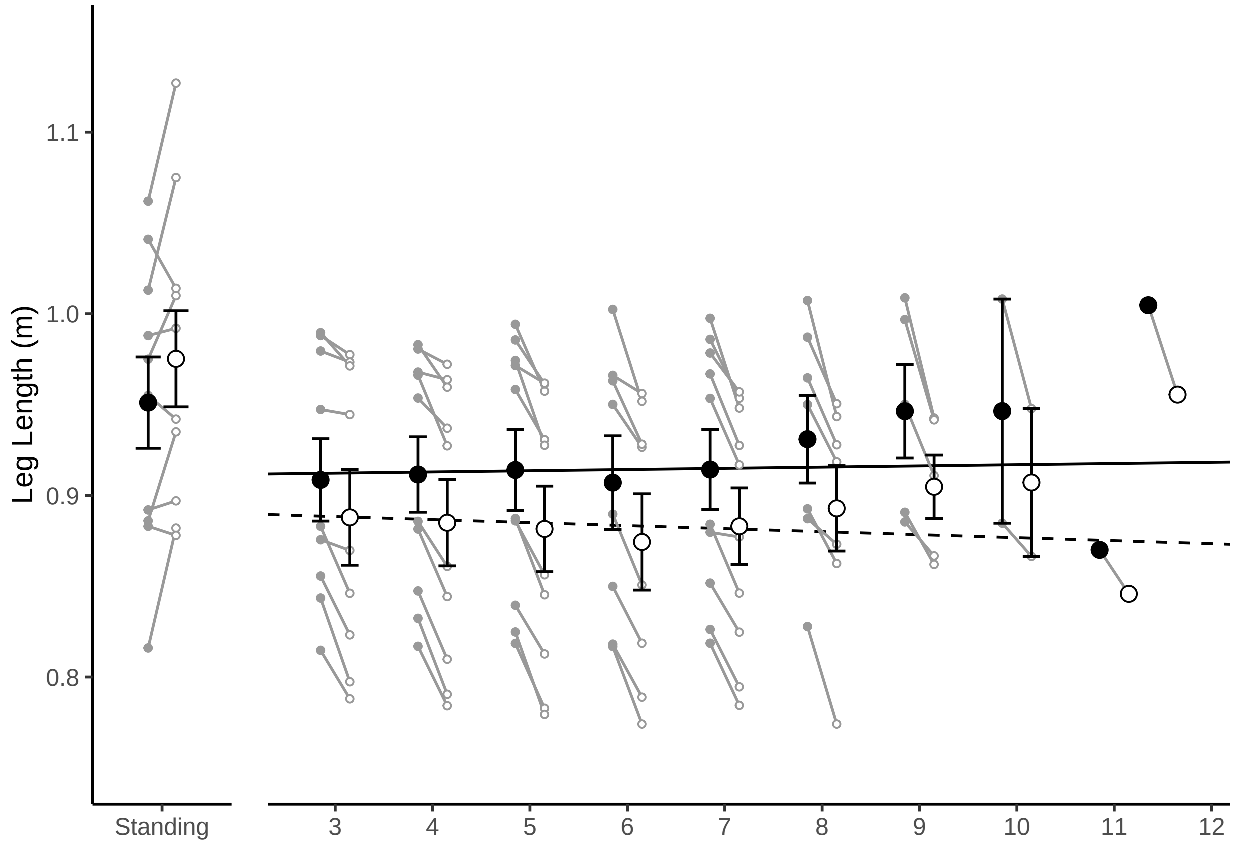

Take-off Plot (ADD LME)

```
#LME from RSP Height Stats.Rmd
LegLengthTO <- lmer(length_to ~ Speed  + Leg.Type + Leg.Type*Speed + (1|SubID), data=unilateral_data.ht

#Fixed effects from the model
LegLengthTO_effects <- as.data.frame(fixef(LegLengthTO))
LegLengthTO_int <- LegLengthTO_effects[1,1]
LegLengthTO_speed <- LegLengthTO_effects[2,1]
LegLengthTO_type_pl <- LegLengthTO_effects[3,1]
LegLengthTO_speed_type_pl <- LegLengthTO_effects[4,1]

#Take-off Graph
ex_to4 <- ggplot(data = unilateral_data.ht0_whole, aes(x=Speed_shift, y=length_to, fill = Leg.Type, sha
  scale_fill_manual(values = c("black", "white"))+
  scale_shape_manual(values = c(19, 21))+
  scale_color_manual(values = c("gray60", "gray60","gray60", "gray60","gray60", "gray60","gray60", "gra
  geom_abline(slope = LegLengthTO_speed, intercept = LegLengthTO_int)+ #biological leg
  geom_abline(slope = LegLengthTO_speed + LegLengthTO_speed_type_pl, intercept = LegLengthTO_int + LegL
  geom_line(aes(group = SpeedSub, col = SubID))+
  geom_point(size = 1, color = "gray60")+
  stat_summary(geom="errorbar", fun.data = mean_se, aes(group = LegSpeed)) +
  stat_summary(geom="point", fun = mean, size = 2.5, aes(group = LegSpeed))+
  scale_x_continuous(breaks = c(3,4,5,6,7,8,9,10,11,12))+
  scale_y_continuous(limits = c(0.75,1.15))+
  labs(x = "Speed (m/s)", y = "Leg Length (m)")+
  theme_classic()+
  theme(legend.position = "none", plot.title = element_text(hjust = 0.5))
```

```
ex_to4
```

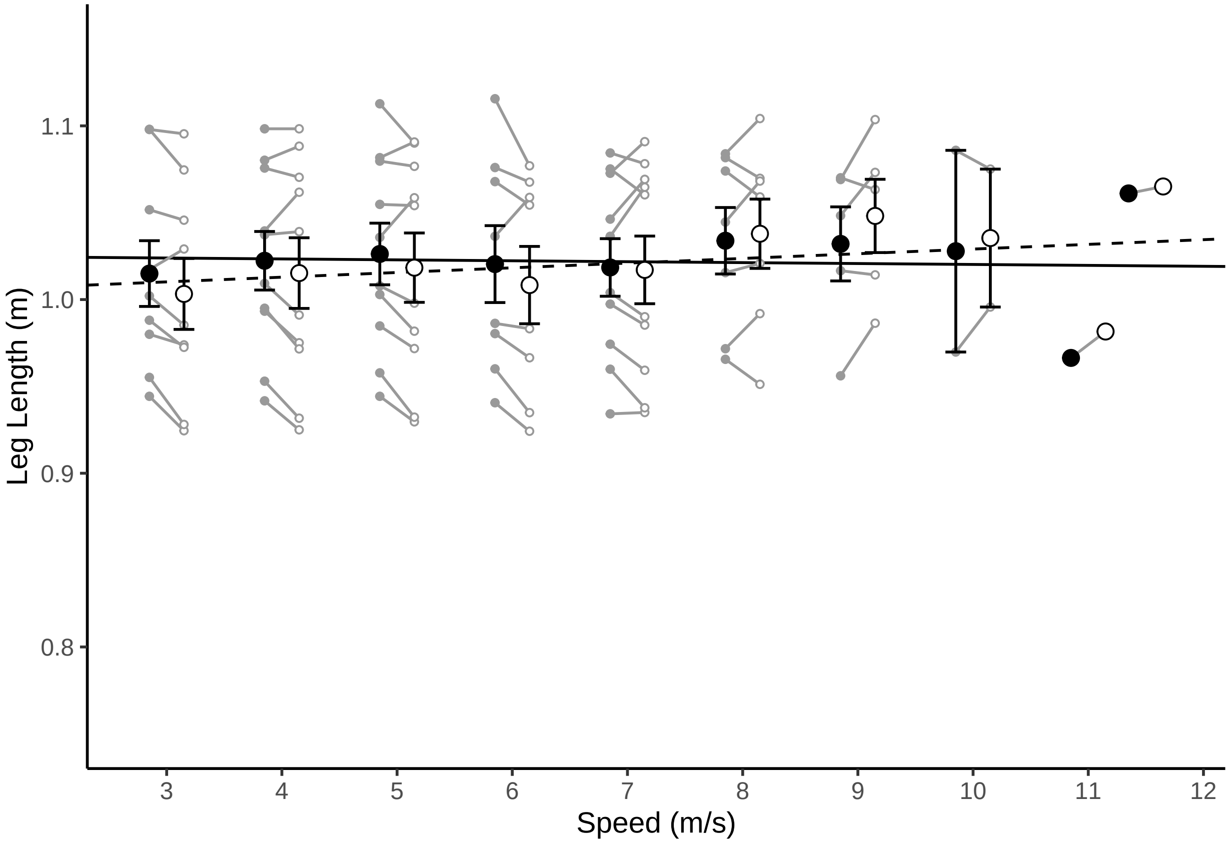


```
#Combine standing and take-off graphs
ex_st_to4 <- plot_grid(ex_st3+theme(axis.title.x=element_blank()), ex_to4+theme(axis.line.y = element_bl
ex_st_to4
```

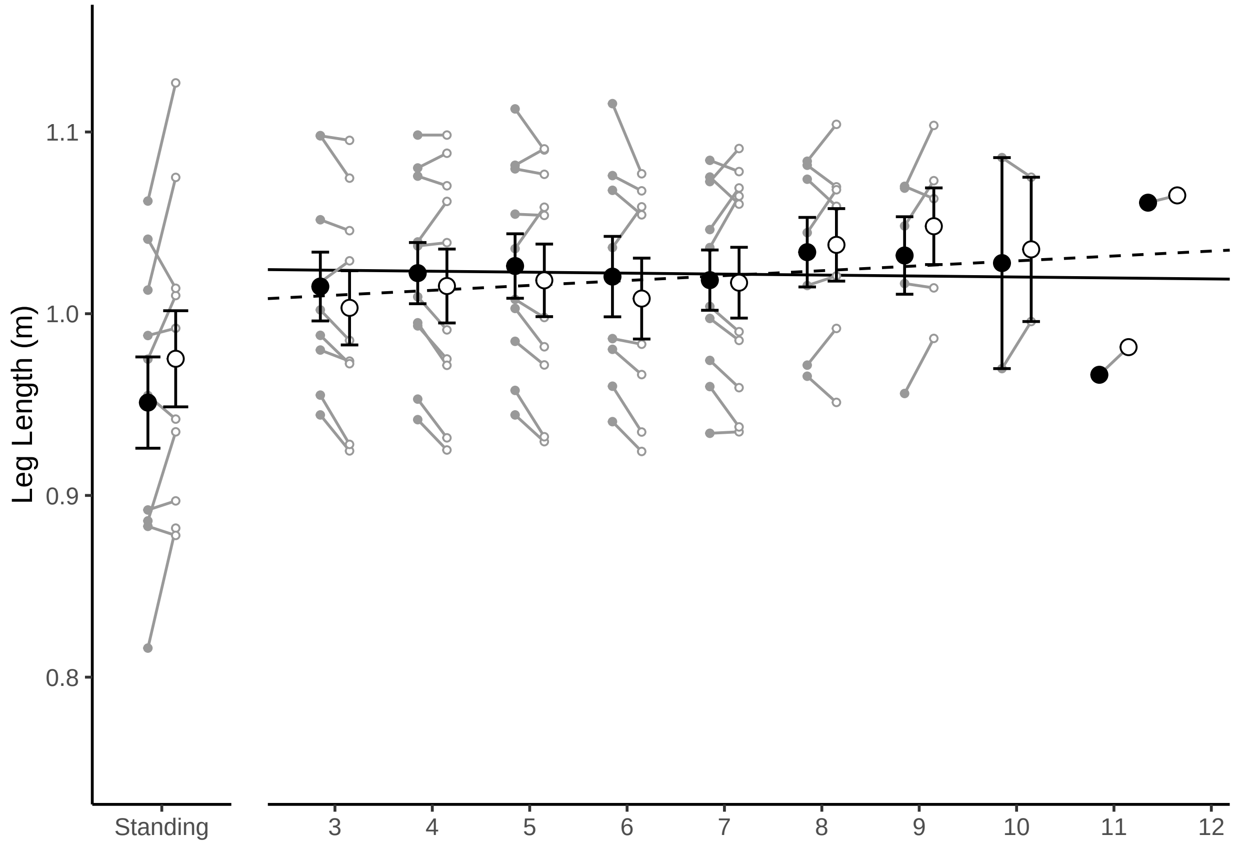

Plot 1

```
#combine touchdown, midstance, and  take-off
ex4 <- plot_grid(ex_st_td4, ex_st_ms4, ex_st_to4, ncol = 1, nrow = 3)
ex4
```

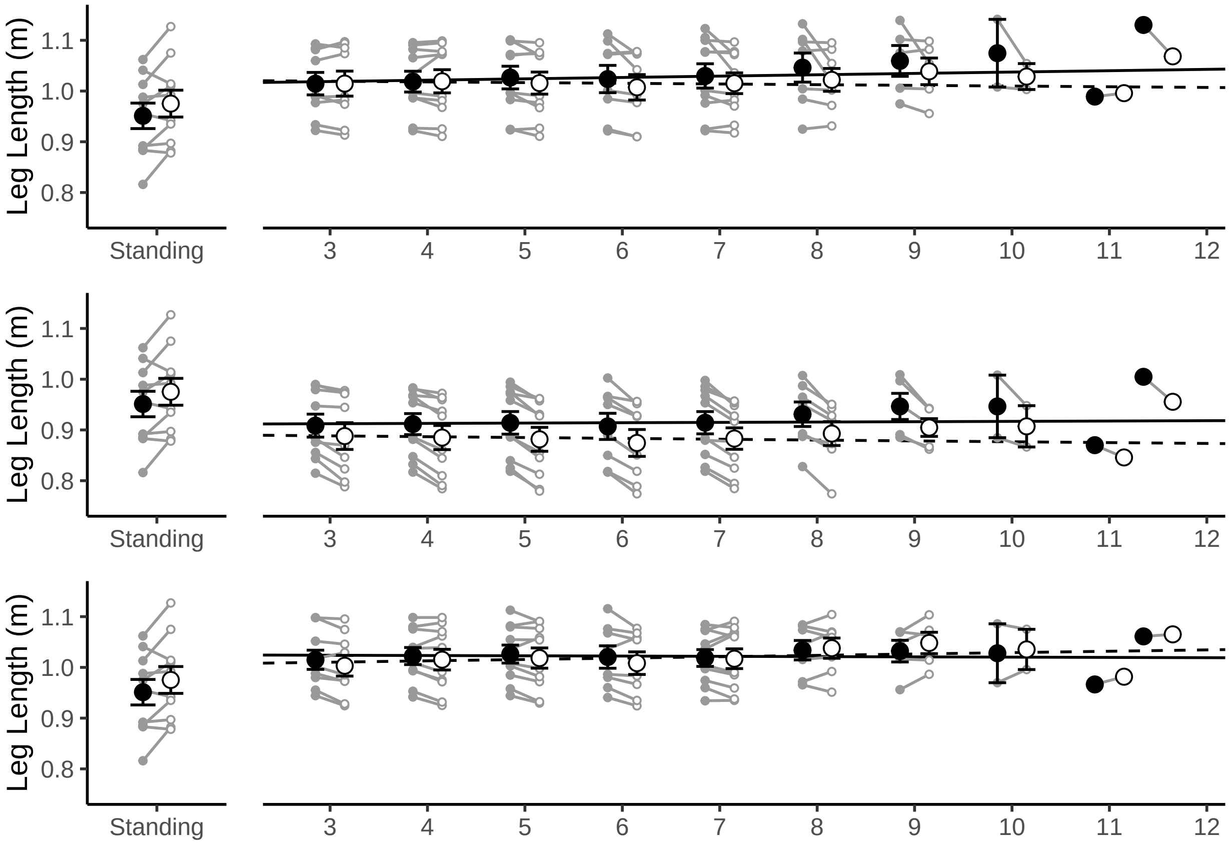

Touchdown Non-amputee and Bilateral Plot

```
#Standing Leg Length Graph
all_st2 <- ggplot(data = standing_data.blna, aes(x=Stand_shift, y=standing_leg_length, fill = Leg.Type,
  scale_fill_manual(values = c("black", "white"))+
  scale_shape_manual(values = c(17, 23))+
  geom_point(size = 1, color = "gray60")+
  stat_summary(geom="errorbar", fun.data = mean_se, aes(group = Leg.Type)) +
  stat_summary(geom="point", fun = mean, size = 2.5, aes(group = Leg.Type))+
  scale_x_continuous(limits = c(1,3), breaks = 2, labels = "Standing", expand = c(0,0))+
  scale_y_continuous(limits = c(0.75,1.15))+
  labs(y = "Leg Length (m)")+
  scale_color_manual(values = c("black", "black","gray")) +
  theme_classic()+
  theme(legend.position = "none", plot.title = element_text(hjust = 0.5))

all_st2
```

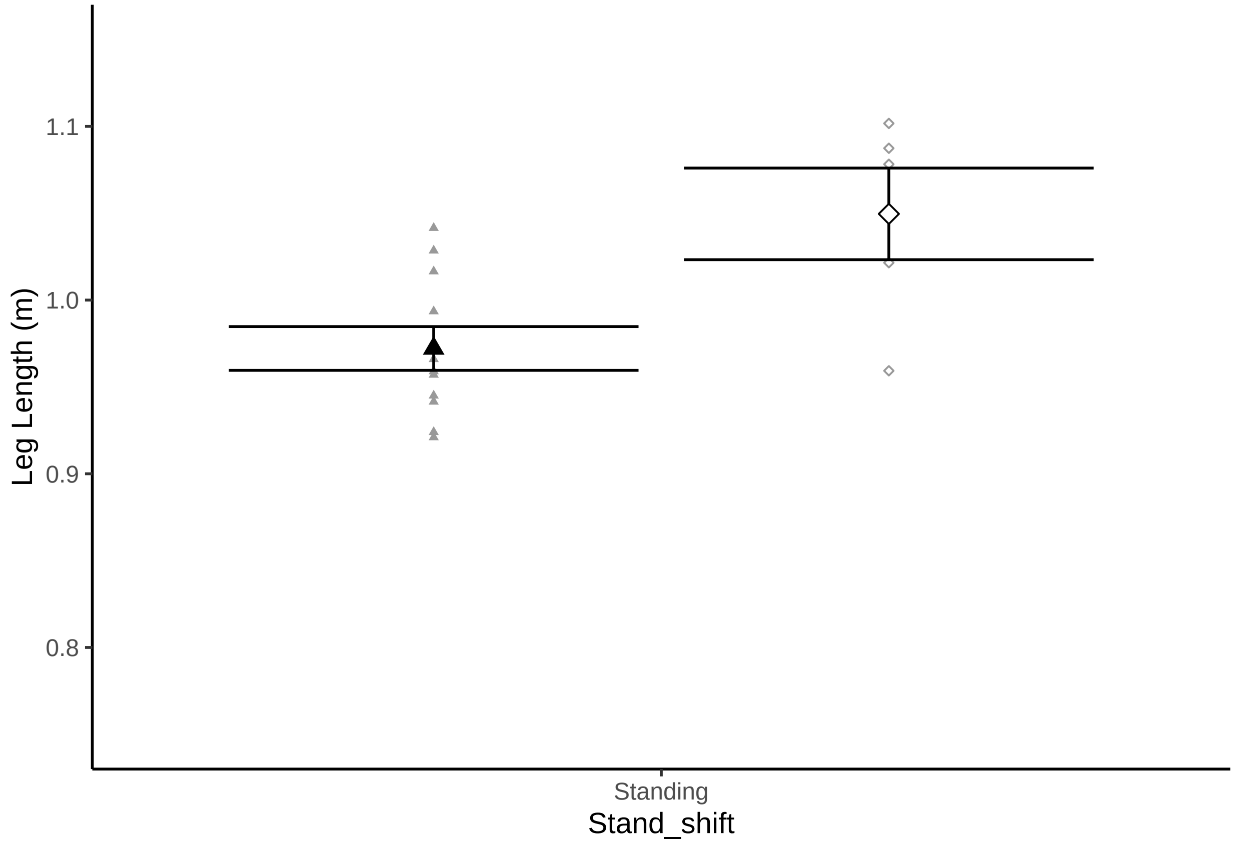


```
#Touchdown Graph
all_td2 <- ggplot(data = rsp_height_data.ht0_blna_whole, aes(x=Speed_shift, y=length_td, fill = Leg.Typ
  scale_fill_manual(values = c("black", "white"))+
  scale_shape_manual(values = c(17, 23))+
  geom_point(size = 1, color = "gray60")+
  stat_summary(geom="errorbar", fun.data = mean_se, aes(group = LegSpeed)) +
  stat_summary(geom="point", fun = mean, size = 2.5, aes(group = LegSpeed))+
  scale_x_continuous(breaks = c(3,4,5,6,7,8,9,10,11,12))+
  scale_y_continuous(limits = c(0.75,1.15))+
  labs(x = "Speed (m/s)", y = "Leg Length (m)")+
  scale_color_manual(values = c("black", "black", "gray")) +
  theme_classic()+
  theme(legend.position = "none", plot.title = element_text(hjust = 0.5))

all_td2
```

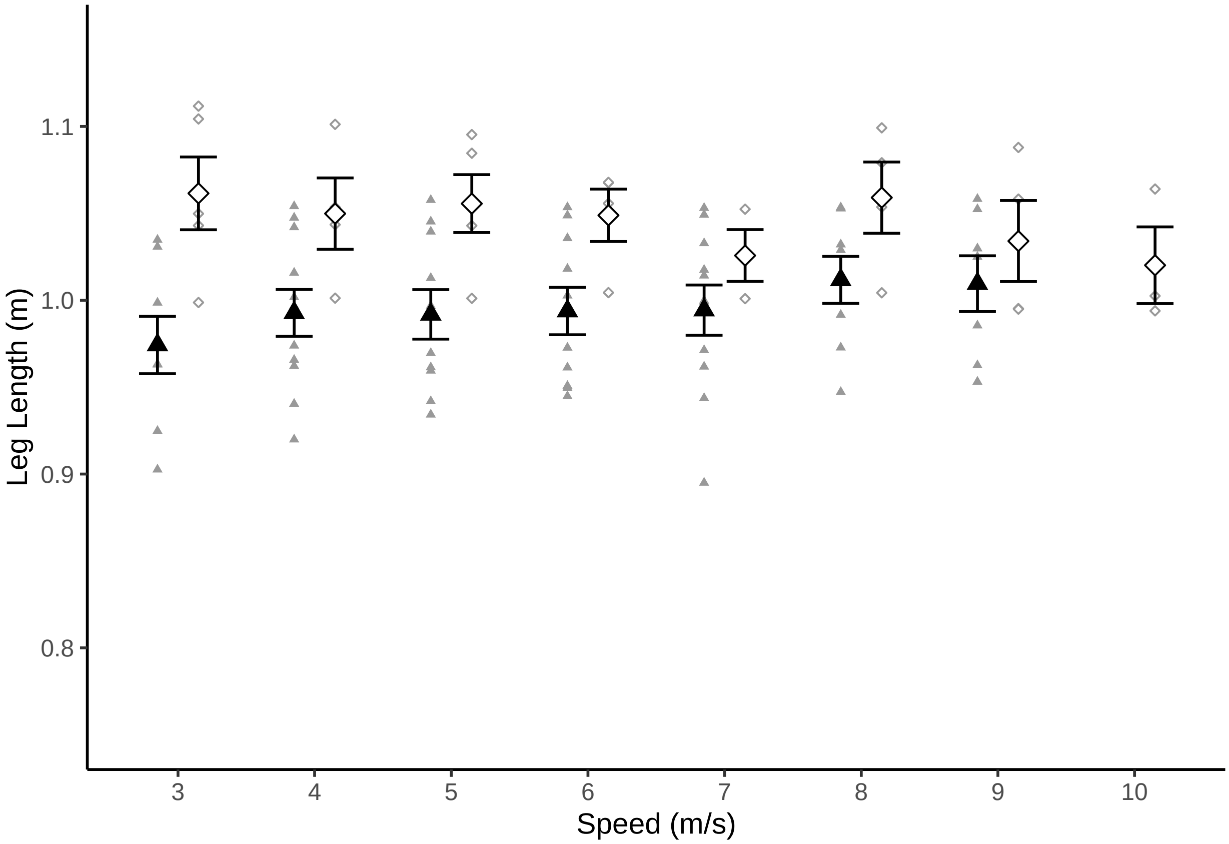


```
#Combine standing and touchdown graphs
all_st_td2 <- plot_grid(all_st2+theme(axis.title.x=element_blank()), all_td2+theme(axis.line.y = elemen
all_st_td2
```

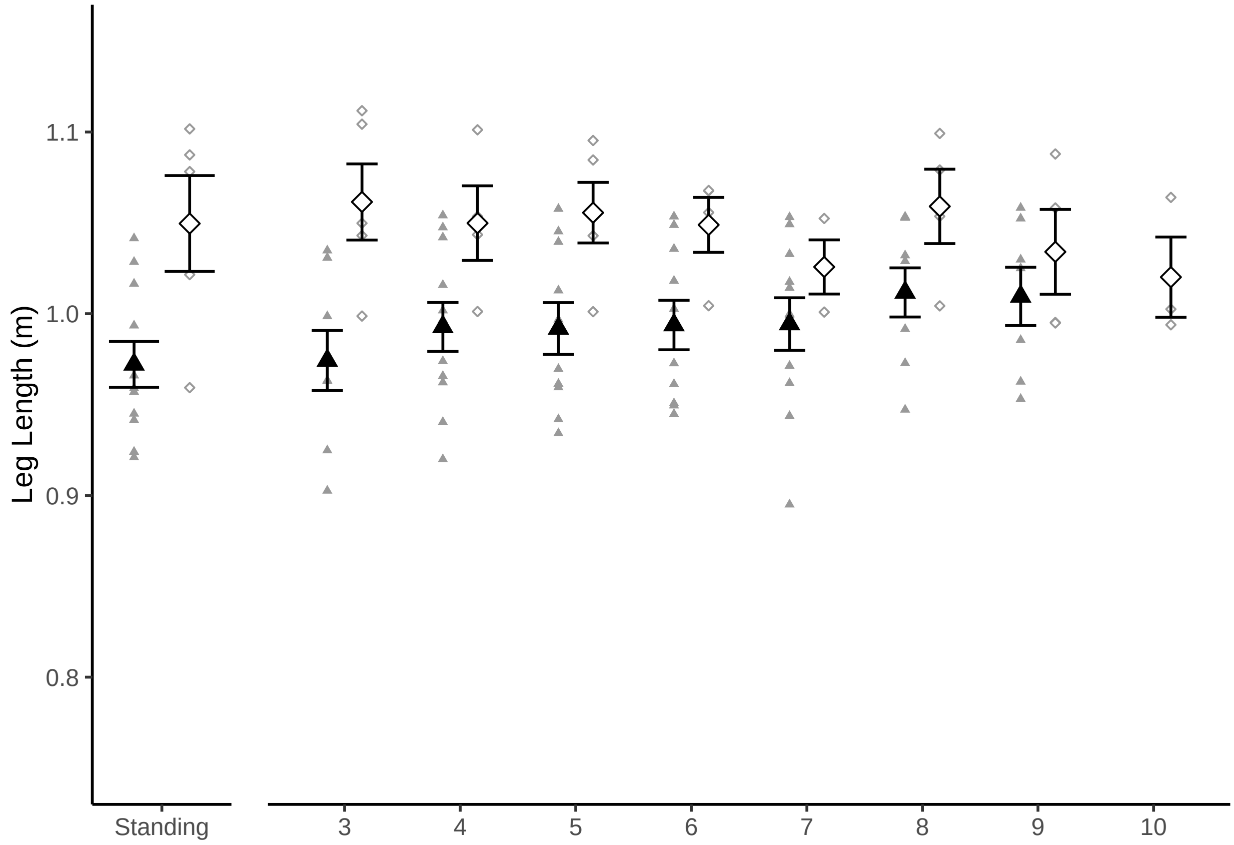

Midstance Non-amputee and Bilateral Plot

```
#Midstance Graph
all_ms2 <- ggplot(data = rsp_height_data.ht0_blna_whole, aes(x=Speed_shift, y=length_ms, fill = Leg.Typ
  scale_fill_manual(values = c("black", "white"))+
  scale_shape_manual(values = c(17, 23))+
  geom_point(size = 1, color = "gray60")+
  stat_summary(geom="errorbar", fun.data = mean_se, aes(group = LegSpeed)) +
  stat_summary(geom="point", fun = mean, size = 2.5, aes(group = LegSpeed))+
  scale_x_continuous(breaks = c(3,4,5,6,7,8,9,10,11,12))+
  scale_y_continuous(limits = c(0.75,1.15))+
  labs(x = "Speed (m/s)", y = "Leg Length (m)")+
  scale_color_manual(values = c("black", "black", "gray")) +
  theme_classic()+
  theme(legend.position = "none", plot.title = element_text(hjust = 0.5))

all_ms2
```

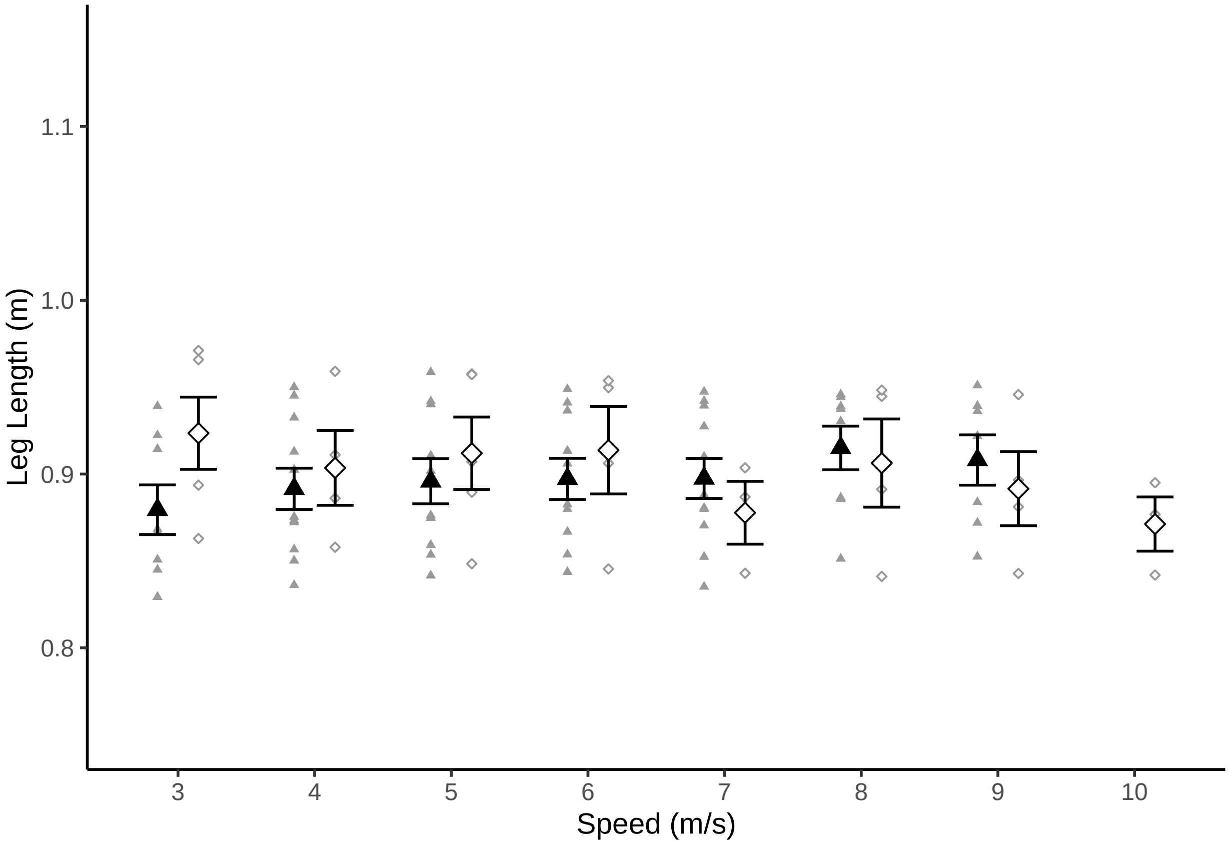


```
#Combine standing and midstance graphs
all_st_ms2 <- plot_grid(all_st2+theme(axis.title.x=element_blank()), all_ms2+theme(axis.line.y = elemen
all_st_ms2
```

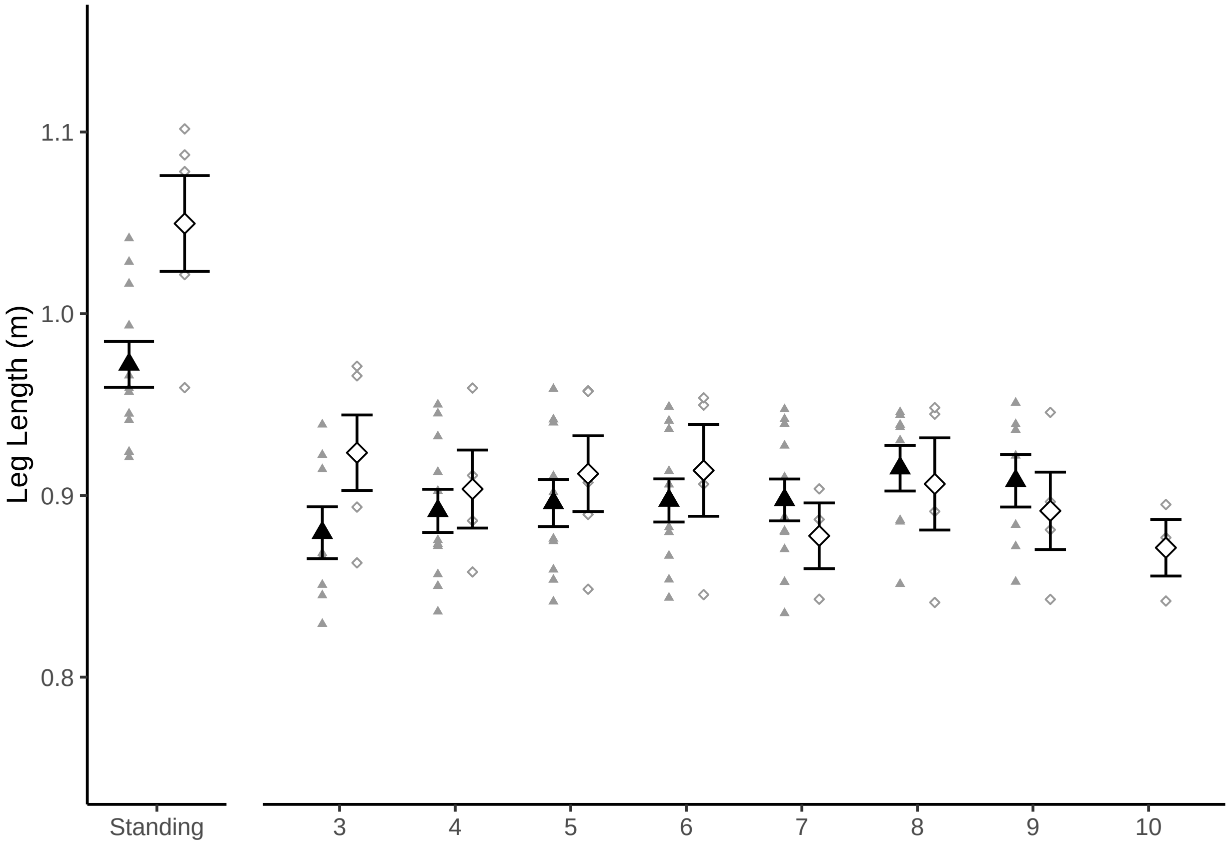

Take-off Non-amputee and Bilateral Plot

```
#Take-off Graph
all_to2 <- ggplot(data = rsp_height_data.ht0_blna_whole, aes(x=Speed_shift, y=length_to, fill = Leg.Type
  scale_fill_manual(values = c("black", "white"))+
  scale_shape_manual(values = c(17, 23))+
  geom_point(size = 1, color = "gray60")+
  stat_summary(geom="errorbar", fun.data = mean_se, aes(group = LegSpeed)) +
  stat_summary(geom="point", fun = mean, size = 2.5, aes(group = LegSpeed))+
  scale_x_continuous(breaks = c(3,4,5,6,7,8,9,10,11,12))+
  scale_y_continuous(limits = c(0.75,1.15))+
  labs(x = "Speed (m/s)", y = "Leg Length (m)")+
  scale_color_manual(values = c("black", "black", "gray")) +
  theme_classic()+
  theme(legend.position = "none", plot.title = element_text(hjust = 0.5))

all_to2
```

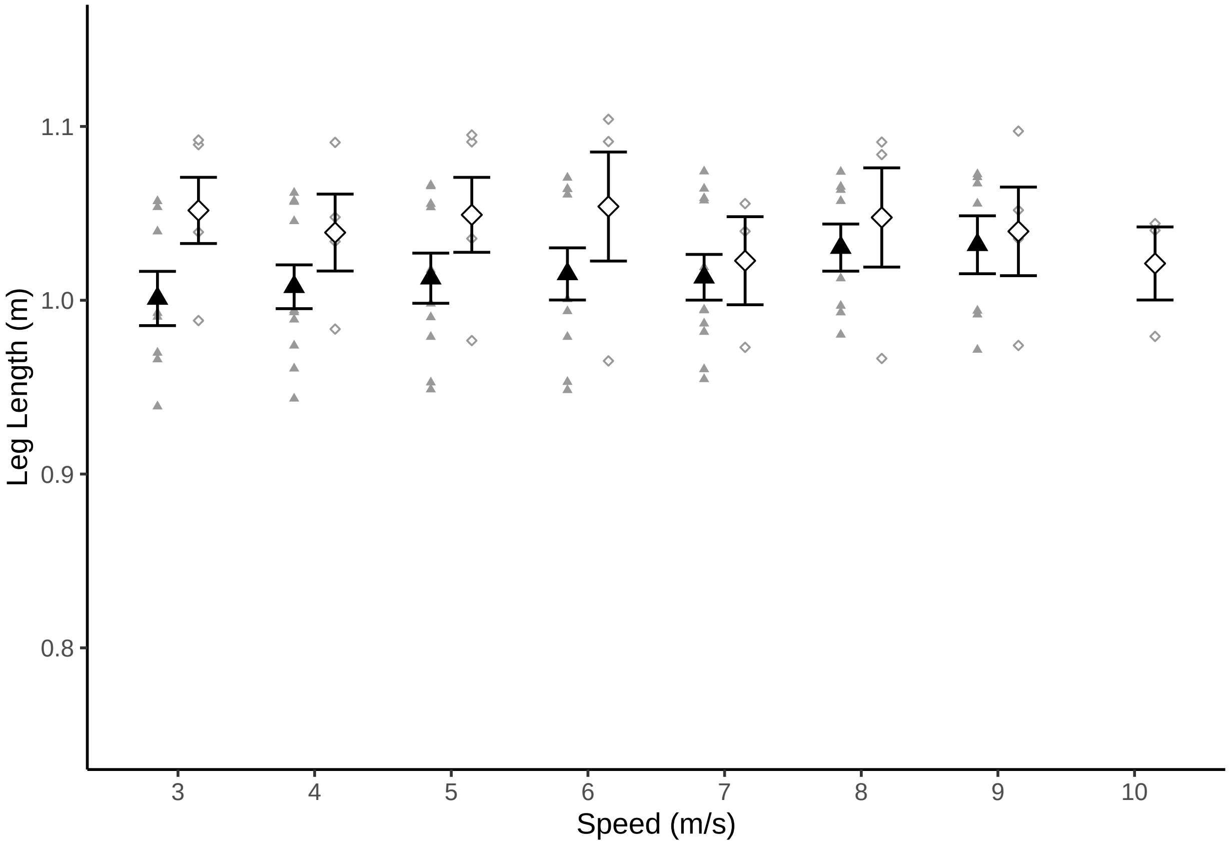


```
#Combine standing and take-off graphs
all_st_to2 <- plot_grid(all_st2+theme(axis.title.x=element_blank()), all_to2+theme(axis.line.y = elemen
all_st_to2
```

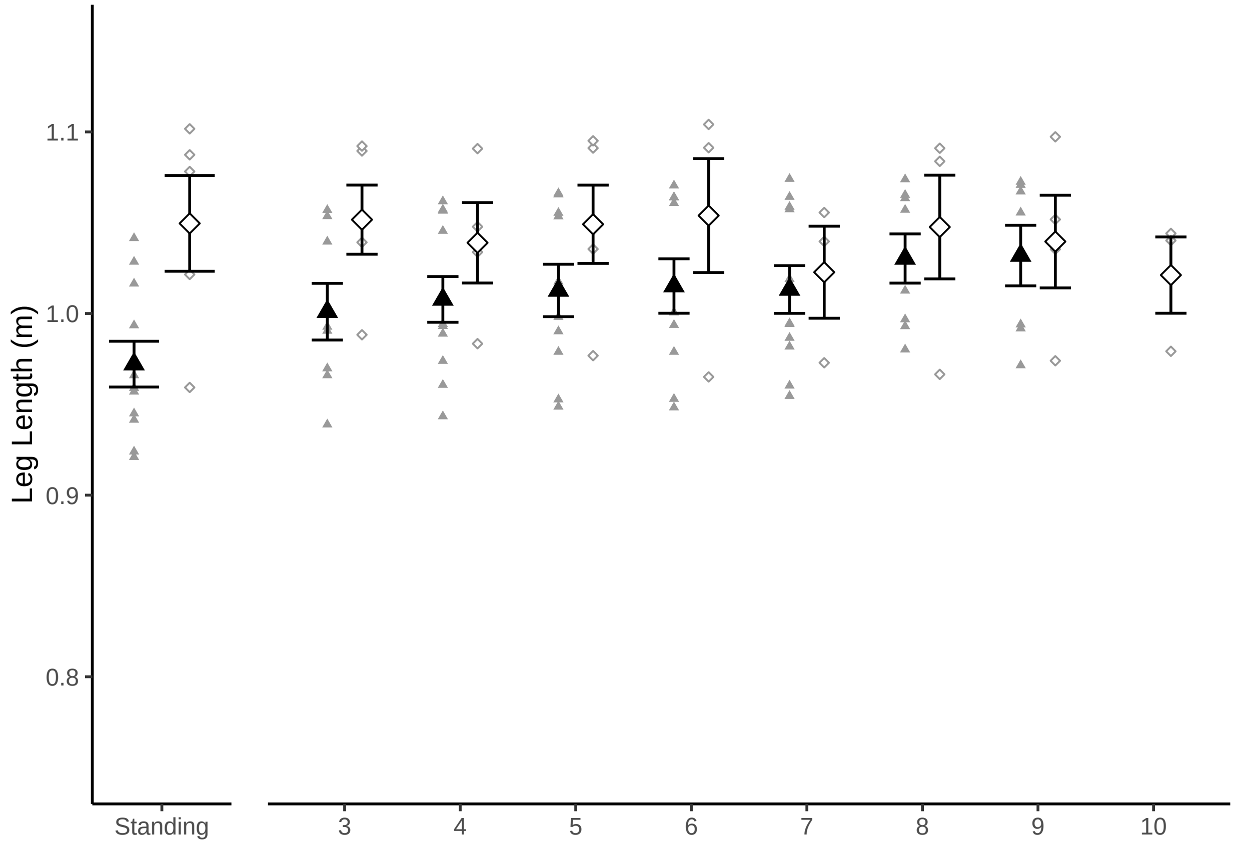

Plot 2

```r
#combine touchdown, midstance, and  take-off
ex6 <- plot_grid(all_st_td2, all_st_ms2, all_st_to2, ncol = 1, nrow = 3)
ex6
```

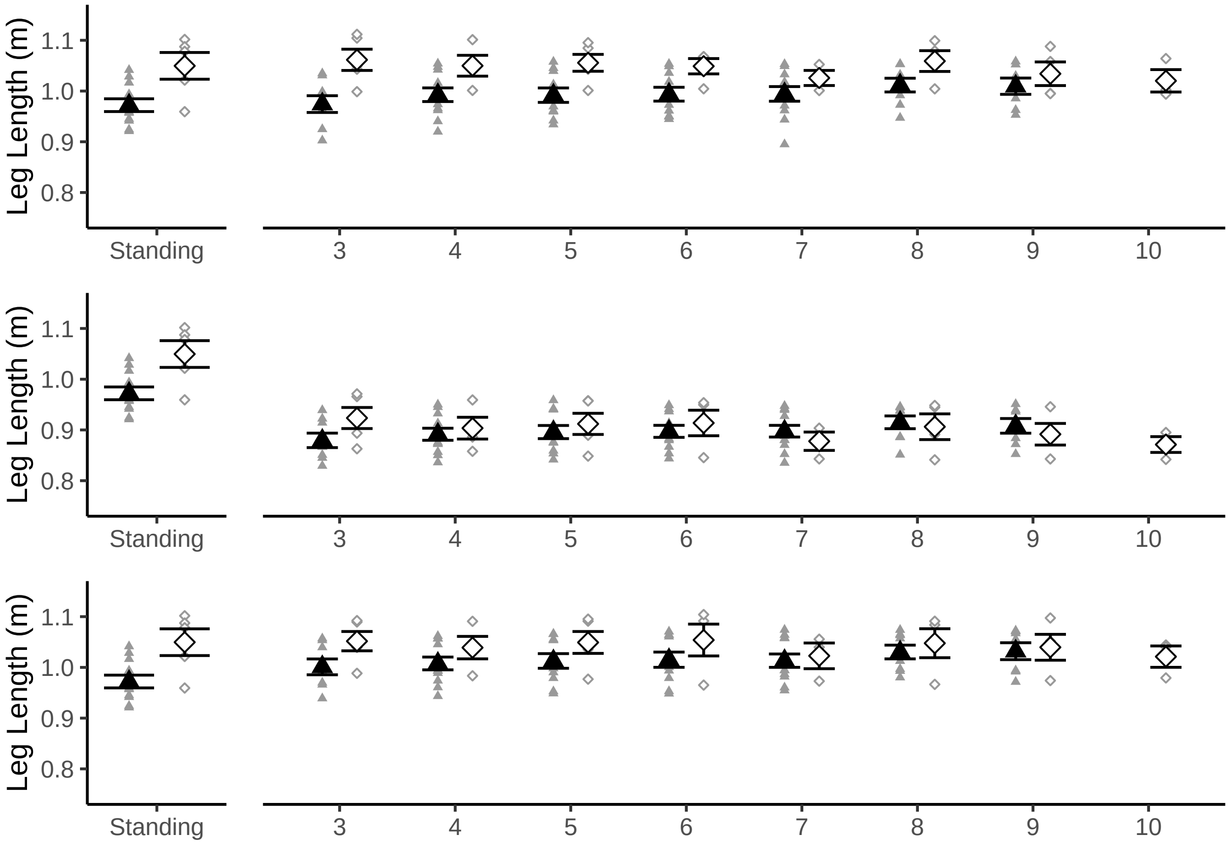

Figure 1 Leg Lengths Plot

```
#Leg length plots with lme (did not fit an lme for bilateral vs. nonamp for leg length plots)
fig1_v2 <- plot_grid(ex4, ex6, ncol = 2, nrow = 1)
fig1_v2
```

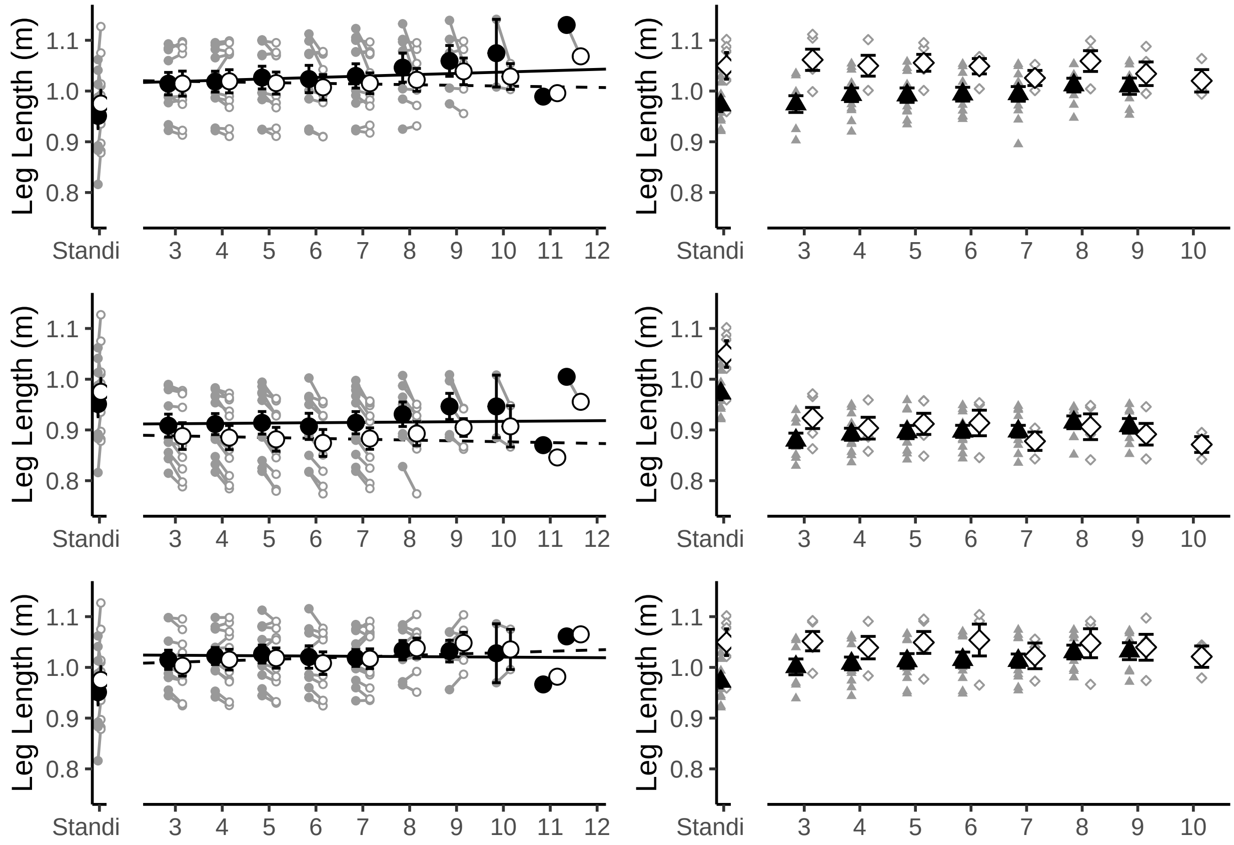


```
ggsave("fig1_leg_length_v2.pdf", plot = fig1_v2, device = "pdf", useDingbats = FALSE, width = 10, height
```

Leg Length Ratio Plots

Unilateral

Touchdown (ADD LME)

```
#LME from Stats.Rmd
LegRatioTD <- lmer(ratio_td ~ Speed  + Leg.Type + Leg.Type*Speed + (1|SubID), data=unilateral_data.ht0,

#Fixed effects from the model
LegRatioTD_effects <- as.data.frame(fixef(LegRatioTD))
LegRatioTD_int <- LegRatioTD_effects[1,1]
LegRatioTD_speed <- LegRatioTD_effects[2,1]
LegRatioTD_type_pl <- LegRatioTD_effects[3,1]
LegRatioTD_speed_type_pl <- LegRatioTD_effects[4,1]

#Touchdown Graph
rat_td_v2 <- ggplot(data = unilateral_data.ht0_whole, aes(x=Speed_shift, y=ratio_td, fill = Leg.Type, sl
  scale_fill_manual(values = c("black", "white"))+
  scale_shape_manual(values = c(19, 21))+
  geom_line(aes(group = SpeedSub), color = "gray60")+
  geom_point(size = 1, alpha = 1, color = "gray60")+
  stat_summary(geom="errorbar", fun.data = mean_se, aes(group = LegSpeed), color = "black") +
  stat_summary(geom="point", fun = mean, size = 2.5, aes(group = LegSpeed), color = "black")+
  geom_abline(slope = LegRatioTD_speed, intercept = LegRatioTD_int)+ #biological leg
  geom_abline(slope = LegRatioTD_speed + LegRatioTD_speed_type_pl, intercept = LegRatioTD_int + LegRatio
  scale_x_continuous(breaks = c(3,4,5,6,7,8,9,10,11,12))+
```

```
  scale_y_continuous(limits = c(0.75,1.25))+
  labs(x = "Speed (m/s)", y = "Leg Length Ratio")+
  theme_classic()+
  theme(legend.position = "none", plot.title = element_text(hjust = 0.5))

rat_td_v2
```

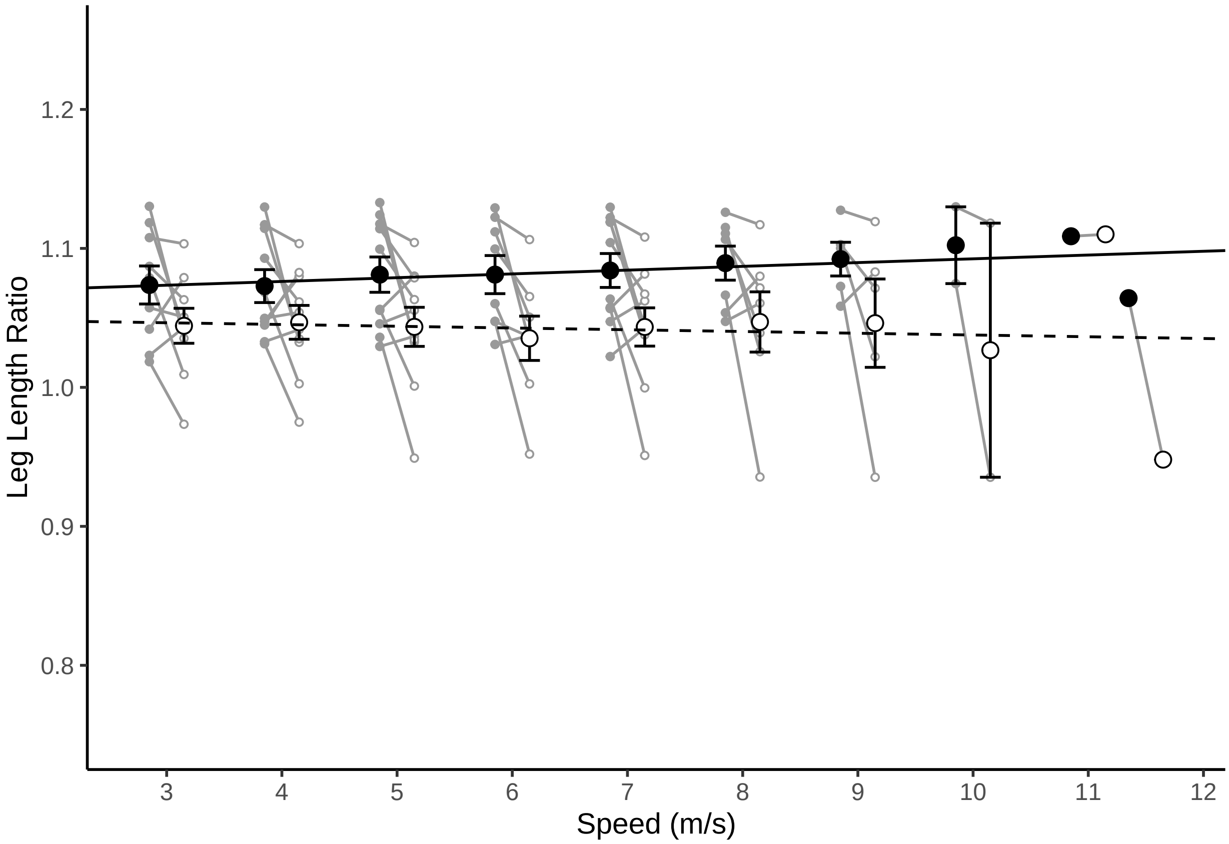

Midstance (ADD LME)

```
#LME from Stats.Rmd
LegRatioMS3 <- lmer(ratio_ms ~  Leg.Type + (1|SubID), data=unilateral_data.ht0, REML=FALSE, na.action=na

#Fixed effects from the model
LegRatioMS3_effects <- as.data.frame(fixef(LegRatioMS3))
LegRatioMS3_int <- LegRatioMS3_effects[1,1]
LegRatioMS3_type_pl <- LegRatioMS3_effects[2,1]

#Midstance Graph
rat_ms_v2 <- ggplot(data = unilateral_data.ht0_whole, aes(x=Speed_shift, y=ratio_ms, fill = Leg.Type, sh
  scale_fill_manual(values = c("black", "white"))+
  scale_shape_manual(values = c(19, 21))+
  geom_line(aes(group = SpeedSub), color = "gray60")+
  geom_point(size = 1, alpha = 1, color = "gray60")+
  stat_summary(geom="errorbar", fun.data = mean_se, aes(group = LegSpeed), color = "black") +
  stat_summary(geom="point", fun = mean, size = 2.5, aes(group = LegSpeed), color = "black")+
  geom_abline(slope = 0, intercept = LegRatioMS3_int)+ #biological leg
  geom_abline(slope = 0, intercept = LegRatioMS3_int + LegRatioMS3_type_pl, linetype = 2)+ #prosthetic
  scale_x_continuous(breaks = c(3,4,5,6,7,8,9,10,11,12))+
```

```
  scale_y_continuous(limits = c(0.75,1.25))+
  labs(x = "Speed (m/s)", y = "Leg Length Ratio")+
  theme_classic()+
  theme(legend.position = "none", plot.title = element_text(hjust = 0.5))

rat_ms_v2
```

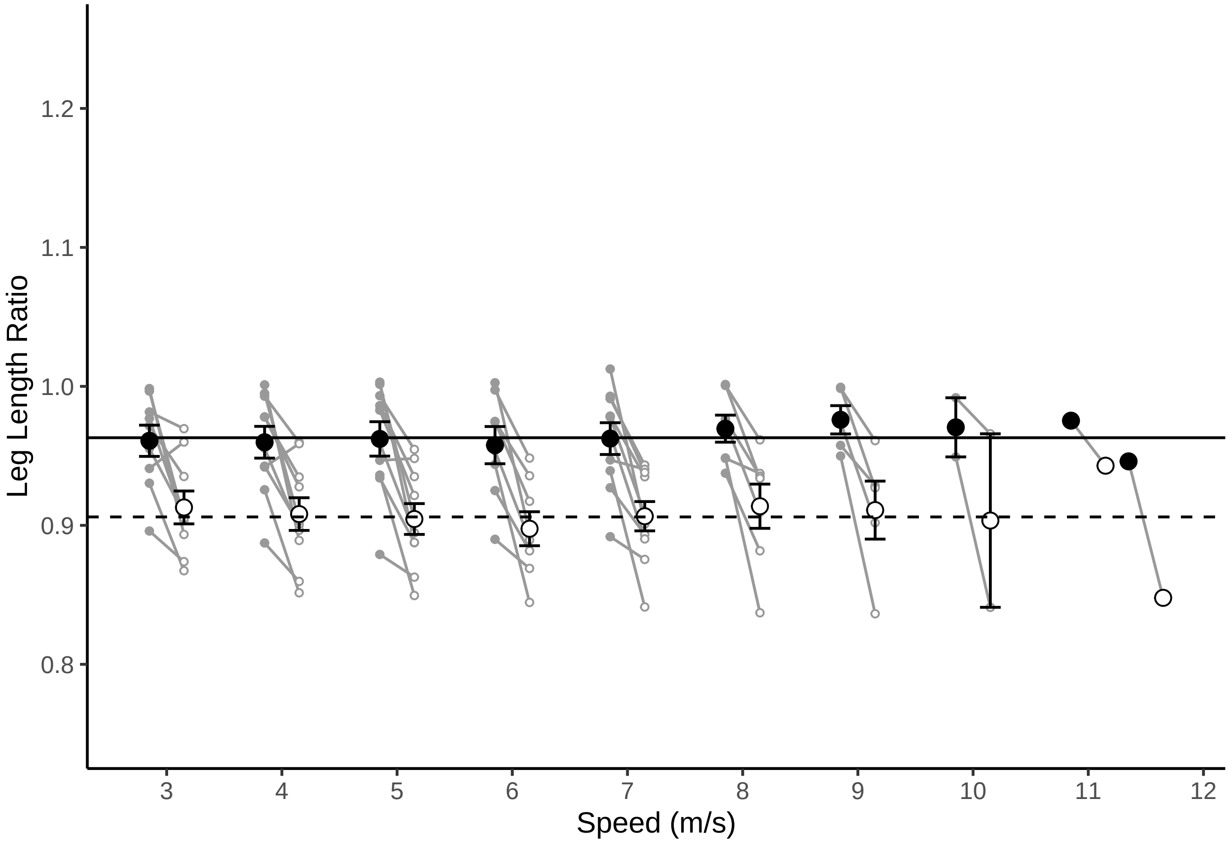

Take-off (ADD LME)

```
#LME from RSP Height Stats.Rmd
LegRatioTO3 <- lmer(ratio_to ~ Leg.Type + (1|SubID), data=unilateral_data.ht0, REML=FALSE, na.action=na

#Fixed effects from the model
LegRatioTO3_effects <- as.data.frame(fixef(LegRatioTO3))
LegRatioTO3_int <- LegRatioTO3_effects[1,1]
LegRatioTO3_type_pl <- LegRatioTO3_effects[2,1]

#Take-off Graph
rat_to_v2 <- ggplot(data = unilateral_data.ht0_whole, aes(x=Speed_shift, y=ratio_to, fill = Leg.Type, sh
  scale_fill_manual(values = c("black", "white"))+
  scale_shape_manual(values = c(19, 21))+
  geom_line(aes(group = SpeedSub), color = "gray60")+
  geom_point(size = 1, alpha = 1, color = "gray60")+
  stat_summary(geom="errorbar", fun.data = mean_se, aes(group = LegSpeed), color = "black") +
  stat_summary(geom="point", fun = mean, size = 2.5, aes(group = LegSpeed), color = "black")+
  geom_abline(slope = 0, intercept = LegRatioTO3_int)+ #biological leg
  geom_abline(slope = 0, intercept = LegRatioTO3_int + LegRatioTO3_type_pl, linetype = 2)+ #prosthetic
  scale_x_continuous(breaks = c(3,4,5,6,7,8,9,10,11,12))+
```

```
  scale_y_continuous(limits = c(0.75,1.25))+
  labs(x = "Speed (m/s)", y = "Leg Length Ratio")+
  theme_classic()+
  theme(legend.position = "none", plot.title = element_text(hjust = 0.5))

rat_to_v2
```

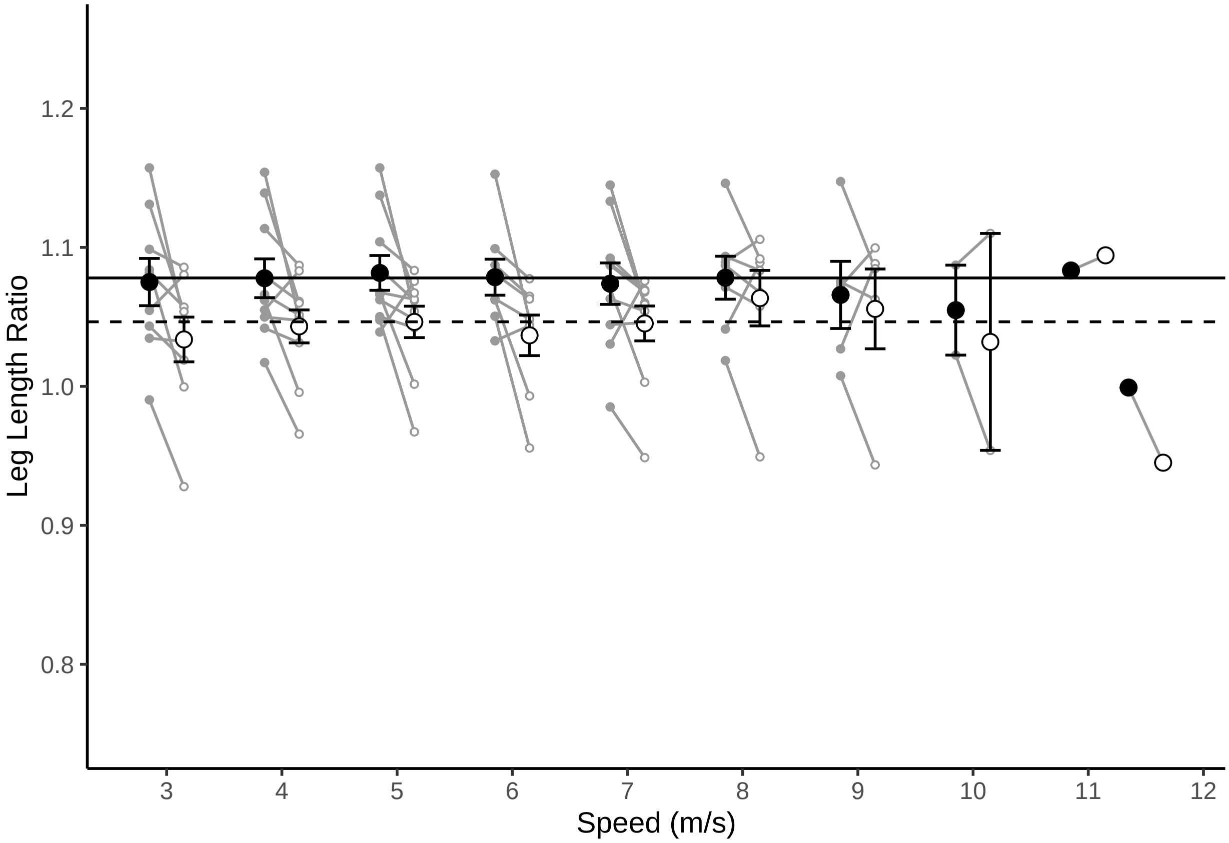

Leg Length Ratio Plot Unilaterals (with LME)

```
#combine touchdown, midstance, and  take-off
rat_v2 <- plot_grid(rat_td_v2, rat_ms_v2, rat_to_v2, ncol = 1, nrow = 3)
rat_v2
```

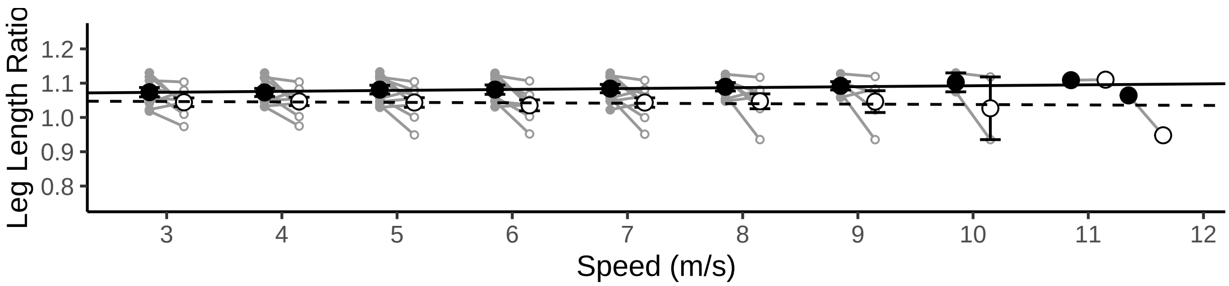

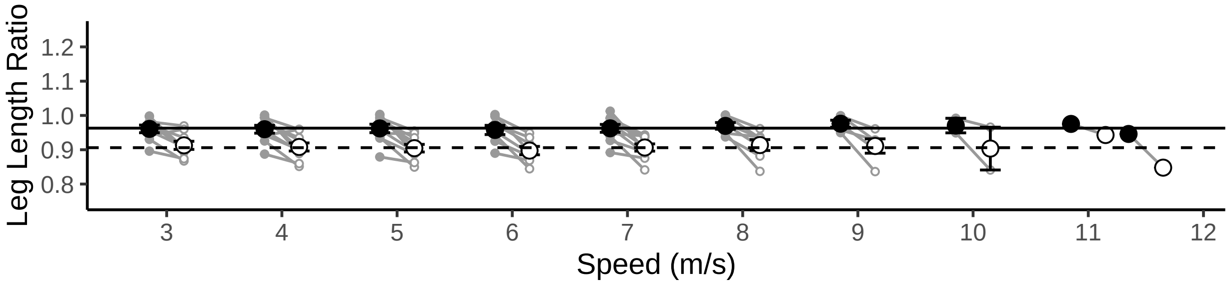

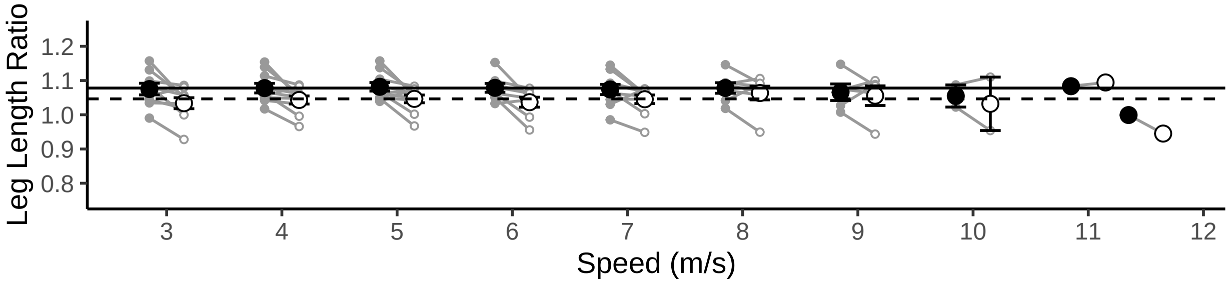

Non-amputee and Bilaterals

Touchdown

```
#LME from Stats.Rmd
LegRatioTDAll <- lmer(ratio_td ~ Speed + AmpType + AmpType*Speed + (1|SubAmp), data=bilatnonamp.ht0, REM

#Fixed effects from the model
LegRatioTDAll_effects <- as.data.frame(fixef(LegRatioTDAll))
LegRatioTDAll_int <- LegRatioTDAll_effects[1,1]
LegRatioTDAll_speed <- LegRatioTDAll_effects[2,1]
LegRatioTDAll_type_nonamp <- LegRatioTDAll_effects[3,1]
LegRatioTDAll_speed_type_nonamp <- LegRatioTDAll_effects[4,1]

#Touchdown Graph + LME
all_rat_td2_lme <- ggplot(data = rsp_height_data.ht0_blna_whole, aes(x=Speed_shift, y=ratio_td, fill = L
  scale_fill_manual(values = c("black", "white"))+
  scale_shape_manual(values = c(17, 23))+
  geom_point(size = 1, alpha = 1, color = "gray60")+
  stat_summary(geom="errorbar", fun.data = mean_se, aes(group = LegSpeed), color = "black") +
  stat_summary(geom="point", fun = mean, size = 2.5, aes(group = LegSpeed), color = "black")+
  geom_abline(slope = LegRatioTDAll_speed + LegRatioTDAll_speed_type_nonamp, intercept = LegRatioTDAll_i
  geom_abline(slope = LegRatioTDAll_speed, intercept = LegRatioTDAll_int, linetype = 2)+ #bilaterals
  scale_x_continuous(breaks = c(3,4,5,6,7,8,9,10,11,12))+
  scale_y_continuous(limits = c(0.75,1.25))+
  labs(x = "Speed (m/s)", y = "Leg Length Ratio")+
  scale_color_manual(values = c("black", "black", "gray")) +
```

```
  theme_classic()+
  theme(legend.position = "none", plot.title = element_text(hjust = 0.5))

all_rat_td2_lme
```

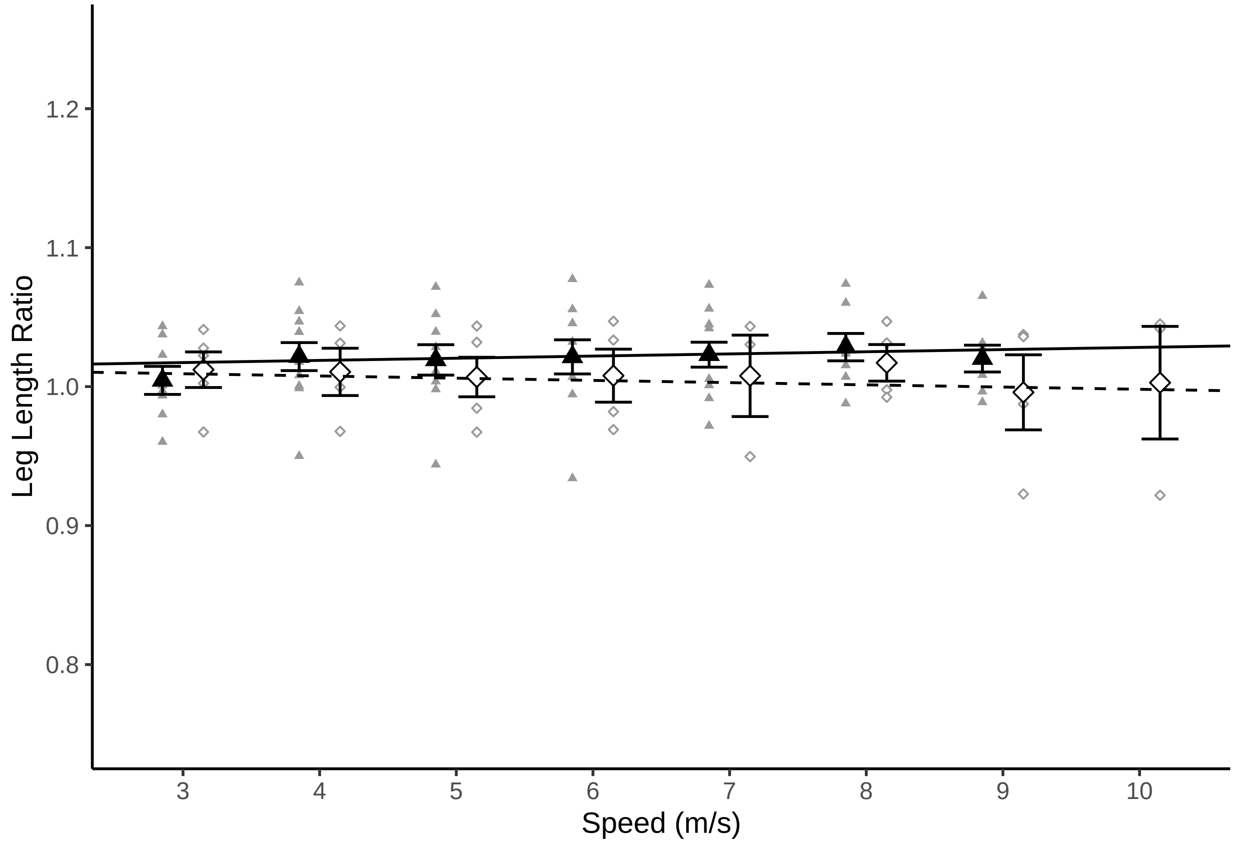

Midstance

```
#LME from Stats.Rmd
LegRatioMSAll <- lmer(ratio_ms ~ Speed + AmpType + AmpType*Speed + (1|SubAmp), data=bilatnonamp.ht0, REI

#Fixed effects from the model
LegRatioMSAll_effects <- as.data.frame(fixef(LegRatioMSAll))
LegRatioMSAll_int <- LegRatioMSAll_effects[1,1]
LegRatioMSAll_speed <- LegRatioMSAll_effects[2,1]
LegRatioMSAll_type_nonamp <- LegRatioMSAll_effects[3,1]
LegRatioMSAll_speed_type_nonamp <- LegRatioMSAll_effects[4,1]

#Midstance Graph + LME
all_rat_ms2_lme <- ggplot(data = rsp_height_data.ht0_blna_whole, aes(x=Speed_shift, y=ratio_ms, fill = l
  scale_fill_manual(values = c("black", "white"))+
  scale_shape_manual(values = c(17, 23))+
  geom_point(size = 1, alpha = 1, color = "gray60")+
  stat_summary(geom="errorbar", fun.data = mean_se, aes(group = LegSpeed), color = "black") +
  stat_summary(geom="point", fun = mean, size = 2.5, aes(group = LegSpeed), color = "black")+
  geom_abline(slope = LegRatioMSAll_speed + LegRatioMSAll_speed_type_nonamp, intercept = LegRatioMSAll_i
  geom_abline(slope = LegRatioMSAll_speed, intercept = LegRatioMSAll_int, linetype = 2)+ #bilaterals
  scale_x_continuous(breaks = c(3,4,5,6,7,8,9,10,11,12))+
  scale_y_continuous(limits = c(0.75,1.25))+
```

```
  labs(x = "Speed (m/s)", y = "Leg Length Ratio")+
  scale_color_manual(values = c("black", "black", "gray")) +
  theme_classic()+
  theme(legend.position = "none", plot.title = element_text(hjust = 0.5))

all_rat_ms2_lme
```

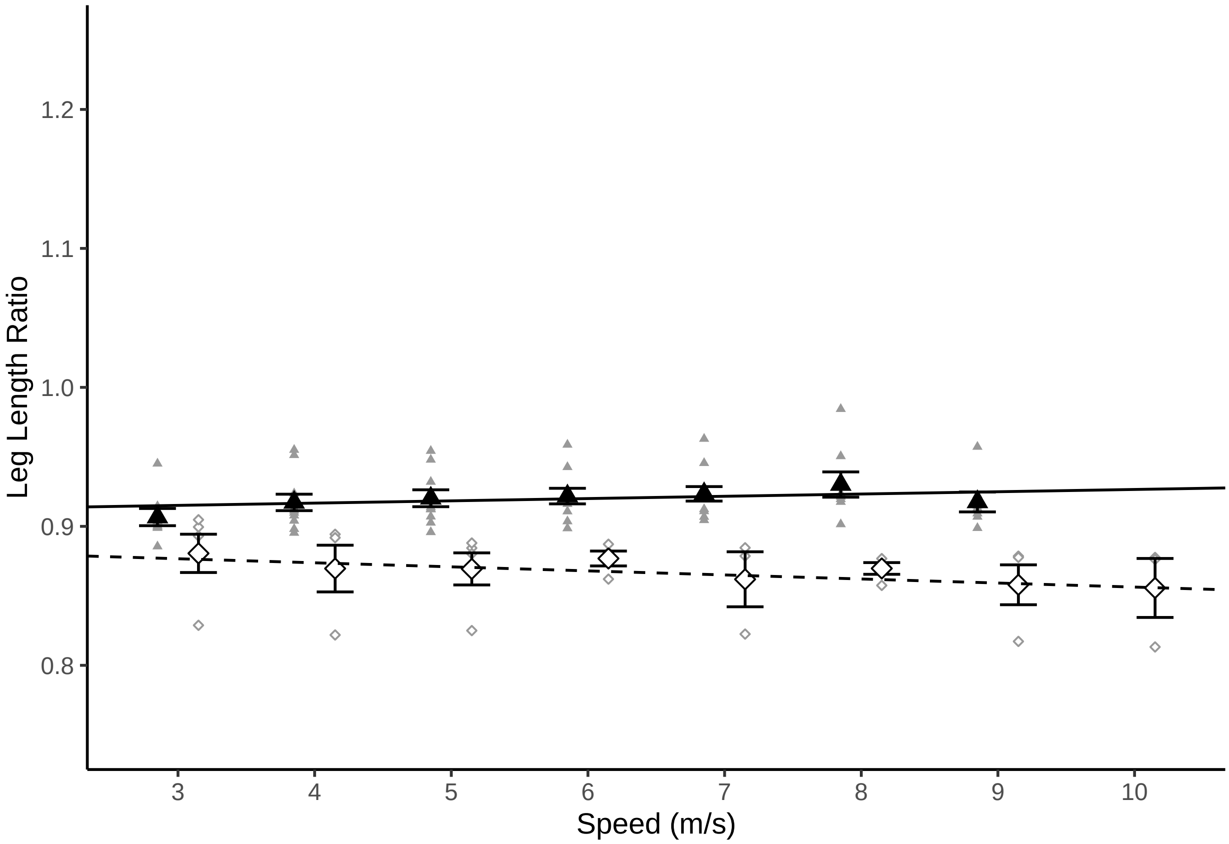

Take-off

```
#LME from Stats.Rmd
LegRatioTOAll <- lmer(ratio_to ~ Speed  + AmpType + AmpType*Speed + (1|SubAmp), data=bilatnonamp.ht0, RE

#Fixed effects from the model
LegRatioTOAll_effects <- as.data.frame(fixef(LegRatioTOAll))
LegRatioTOAll_int <- LegRatioTOAll_effects[1,1]
LegRatioTOAll_speed <- LegRatioTOAll_effects[2,1]
LegRatioTOAll_type_nonamp <- LegRatioTOAll_effects[3,1]
LegRatioTOAll_speed_type_nonamp <- LegRatioTOAll_effects[4,1]

#Take-off Graph + LME
all_rat_to2_lme <- ggplot(data = rsp_height_data.ht0_blna_whole, aes(x=Speed_shift, y=ratio_to, fill = L
  scale_fill_manual(values = c("black", "white"))+
  scale_shape_manual(values = c(17, 23))+
  geom_point(size = 1, alpha = 1, color = "gray60")+
  stat_summary(geom="errorbar", fun.data = mean_se, aes(group = LegSpeed), color = "black") +
  stat_summary(geom="point", fun = mean, size = 2.5, aes(group = LegSpeed), color = "black")+
  geom_abline(slope = LegRatioTOAll_speed + LegRatioTOAll_speed_type_nonamp, intercept = LegRatioTOAll_i
  geom_abline(slope = LegRatioTOAll_speed, intercept = LegRatioTOAll_int, linetype = 2)+ #bilaterals
```

```
  scale_x_continuous(breaks = c(3,4,5,6,7,8,9,10,11,12))+
  scale_y_continuous(limits = c(0.75,1.25))+
  labs(x = "Speed (m/s)", y = "Leg Length Ratio")+
  scale_color_manual(values = c("black", "black", "gray")) +
  theme_classic()+
  theme(legend.position = "none", plot.title = element_text(hjust = 0.5))

all_rat_to2_lme
```

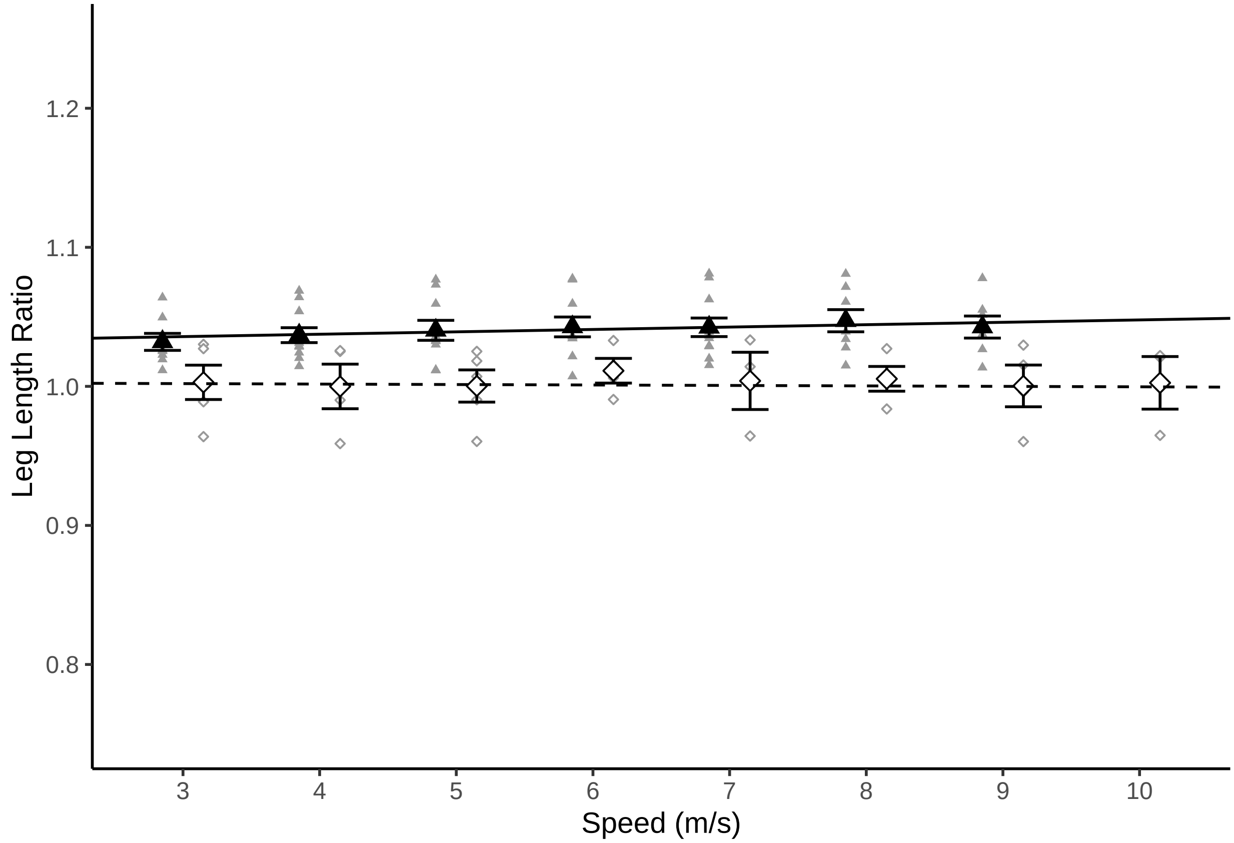

Leg Length Ratio Plot Non-amputee and Bilaterals (with LME)

```
#combine touchdown, midstance, and  take-off
ex8 <- plot_grid(all_rat_td2_lme, all_rat_ms2_lme, all_rat_to2_lme, ncol = 1, nrow = 3)
ex8
```

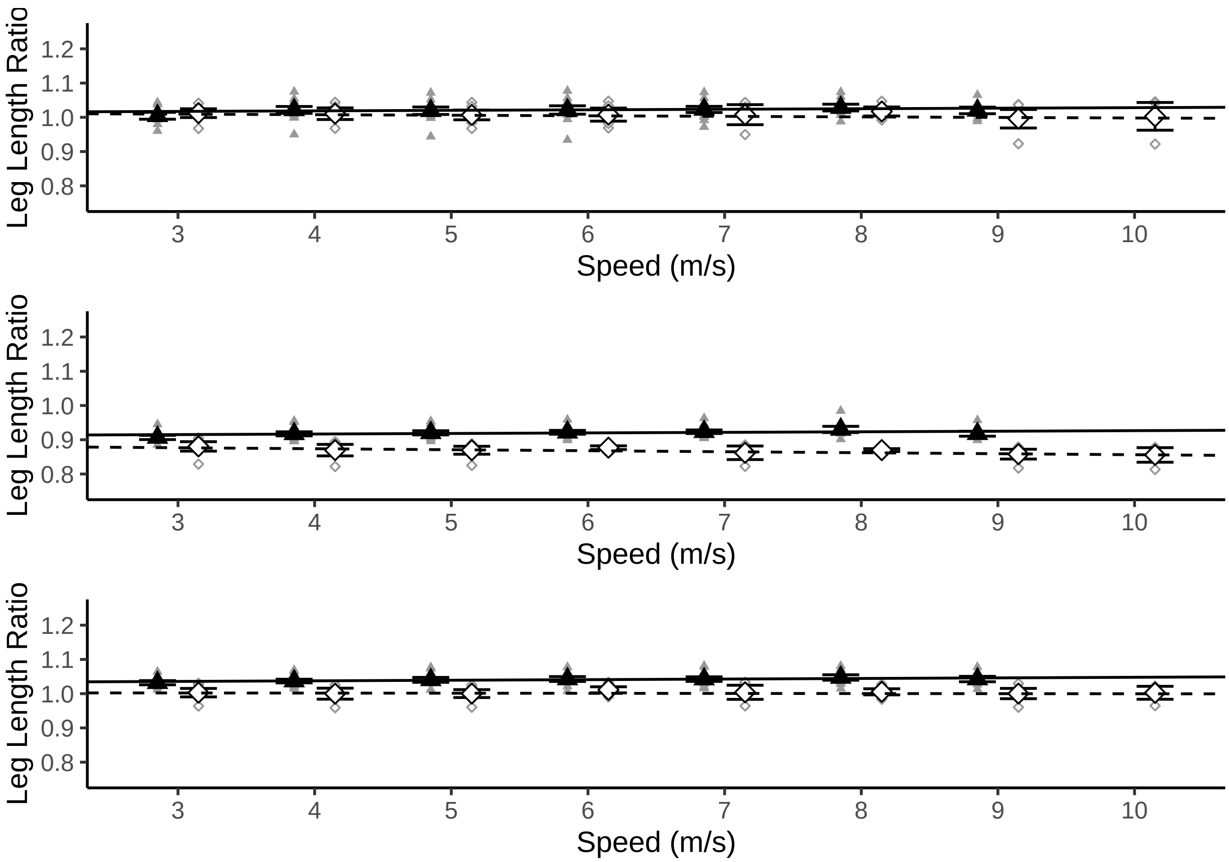

Figure 2 Leg Ratio Plot Version 2 (with LMEs)

```
#Figure 2 leg ratio plot
fig2_v2 <- plot_grid(rat_v2, ex8, ncol = 2, nrow = 1)
fig2_v2
```

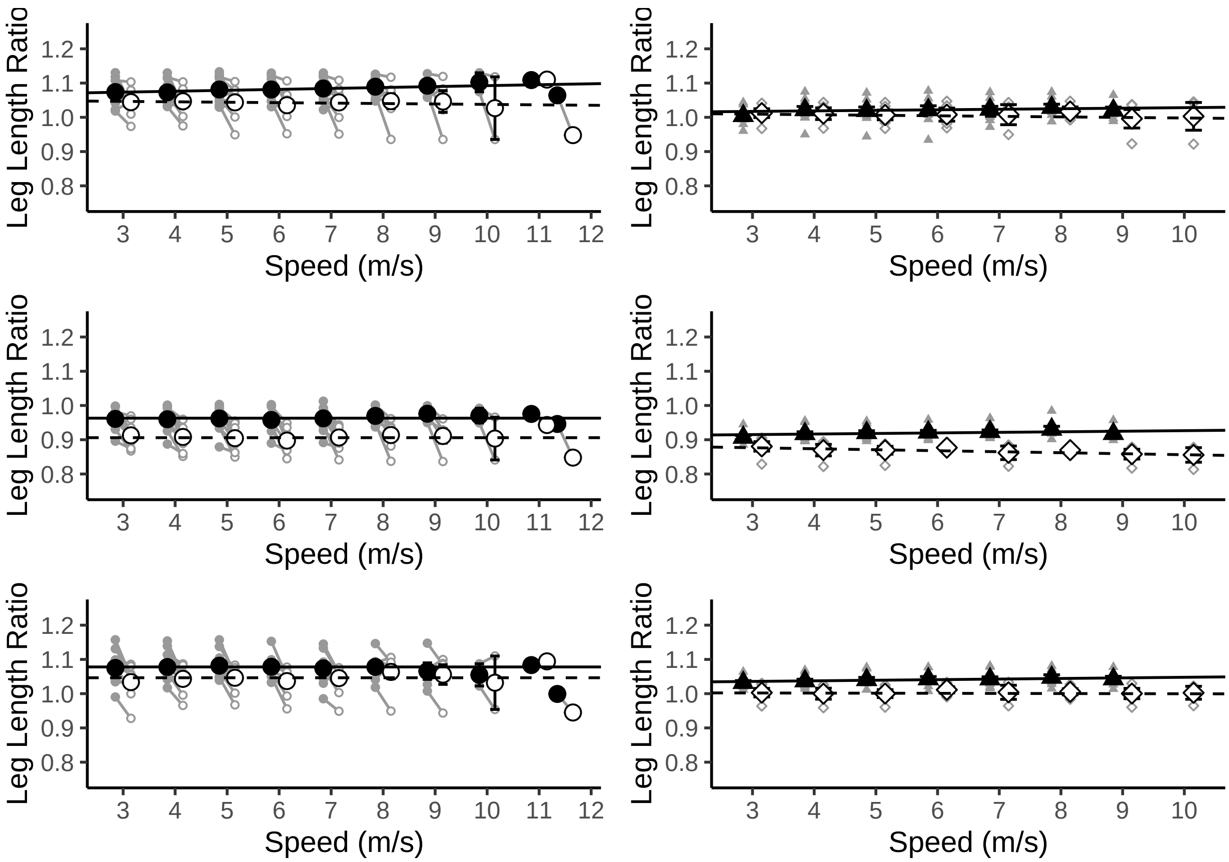


```
ggsave("fig2_leg_ratio_v2.pdf", plot = fig2_v2, device = "pdf", useDingbats = FALSE, width = 10, height
```

Unilateral Standing vs. Running Prosthetic Leg Length

```
#Touchdown
unilat_vs_height_td <- ggplot(data=unilateral_data[unilateral_data$Leg.Type == "PL",], aes(x = Standing_
  geom_point(size = 2, alpha = 1, shape = 21)+
  geom_abline(slope = 1)+
  stat_poly_line(formula = y~x)+
  stat_poly_eq(method = "lm", formula = y~x, output.type = "expression", aes(label = paste(after_stat(eq
  scale_y_continuous(limits = c(0.7,1.15))+
  scale_x_continuous(limits = c(0.7,1.15))+
  labs(x = "Standing Prosthetic Leg Length (m)", y = "Touchdown Prosthetic Leg Length (m)")+
  theme_classic()+
  theme(plot.title = element_text(hjust = 0.5))

unilat_vs_height_td
```

```
## Warning: Not enough data to perform fit for group 8; computing mean instead.
```

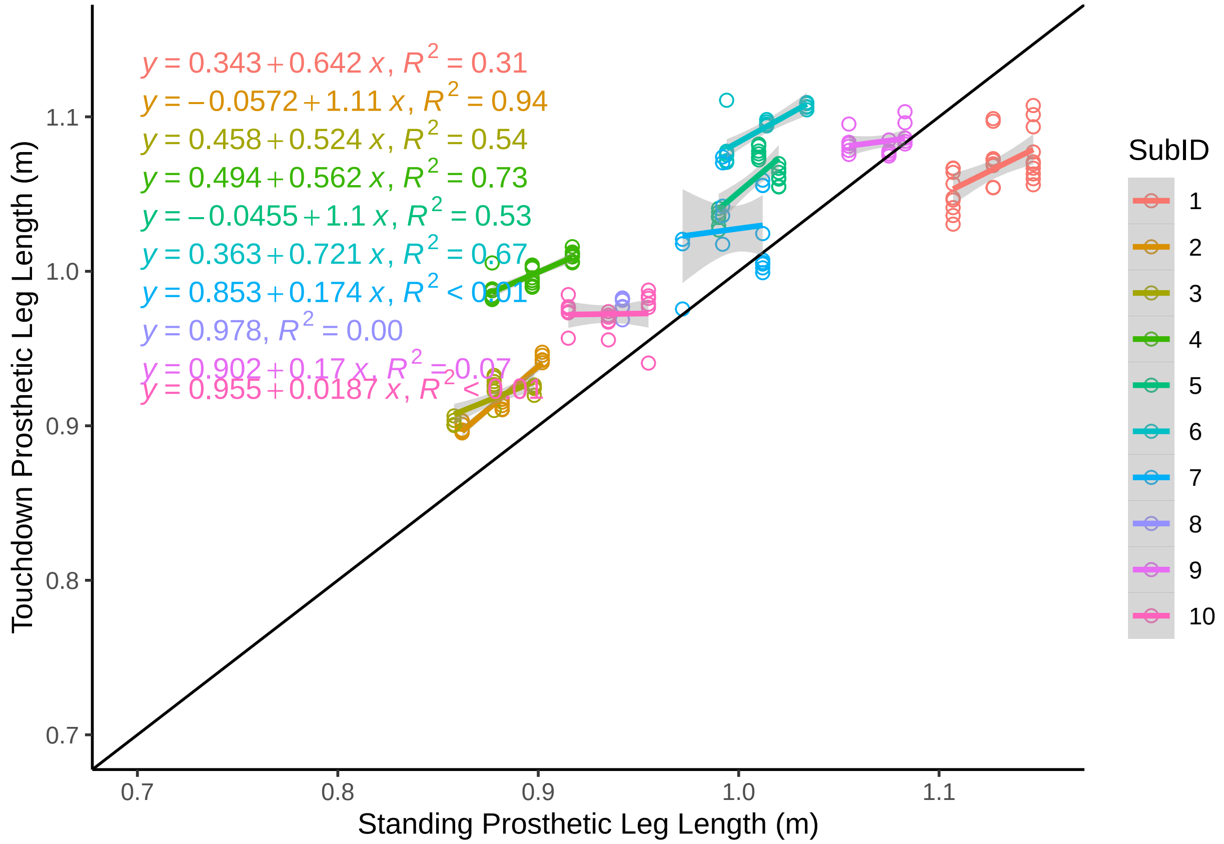


```
#Midstance
unilat_vs_height_ms <- ggplot(data=unilateral_data[unilateral_data$Leg.Type == "PL",], aes(x = Standing_
  geom_point(size = 2, alpha = 1, shape = 21)+
  geom_abline(slope = 1)+
  stat_poly_line(formula = y~x)+
  stat_poly_eq(method = "lm", formula = y~x, output.type = "expression", aes(label = paste(after_stat(eq
  scale_y_continuous(limits = c(0.7,1.15))+
  scale_x_continuous(limits = c(0.7,1.15))+
  labs(x = "Standing Prosthetic Leg Length (m)", y = "Midstance Prostheticc Leg Length (m)")+
  theme_classic()+
  theme(plot.title = element_text(hjust = 0.5))

unilat_vs_height_ms
```

```
## Warning: Not enough data to perform fit for group 8; computing mean instead.
```

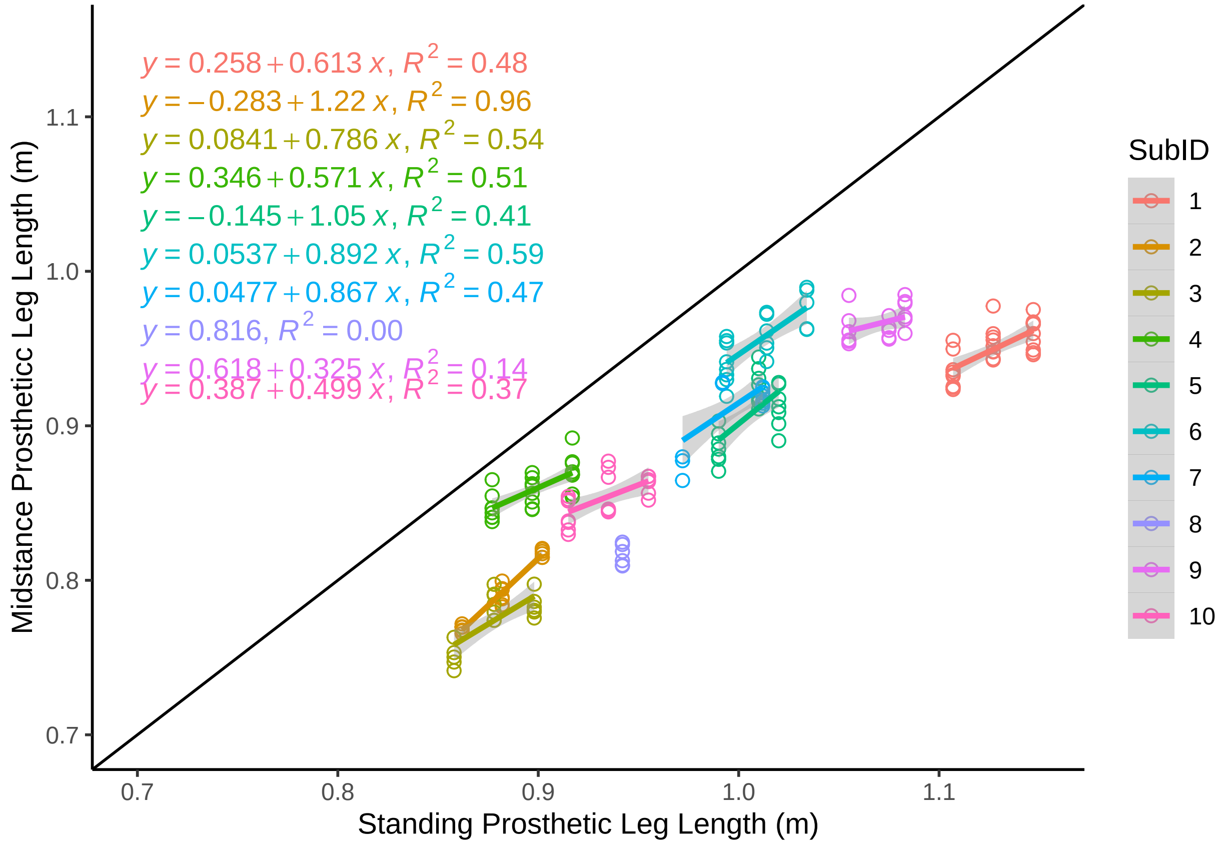


```
#Take-off
unilat_vs_height_to <- ggplot(data=unilateral_data[unilateral_data$Leg.Type == "PL",], aes(x = Standing
  geom_point(size = 2, alpha = 1, shape = 21)+
  geom_abline(slope = 1)+
  stat_poly_line(formula = y~x)+
  stat_poly_eq(method = "lm", formula = y~x, output.type = "expression", aes(label = paste(after_stat(e
  scale_y_continuous(limits = c(0.7,1.15))+
  scale_x_continuous(limits = c(0.7,1.15))+
  labs(x = "Standing Prosthetic Leg Length (m)", y = "Take-off Prosthetic Leg Length (m)")+
  theme_classic()+
  theme(plot.title = element_text(hjust = 0.5))

unilat_vs_height_to
```

```
## Warning: Not enough data to perform fit for group 8; computing mean instead.
```

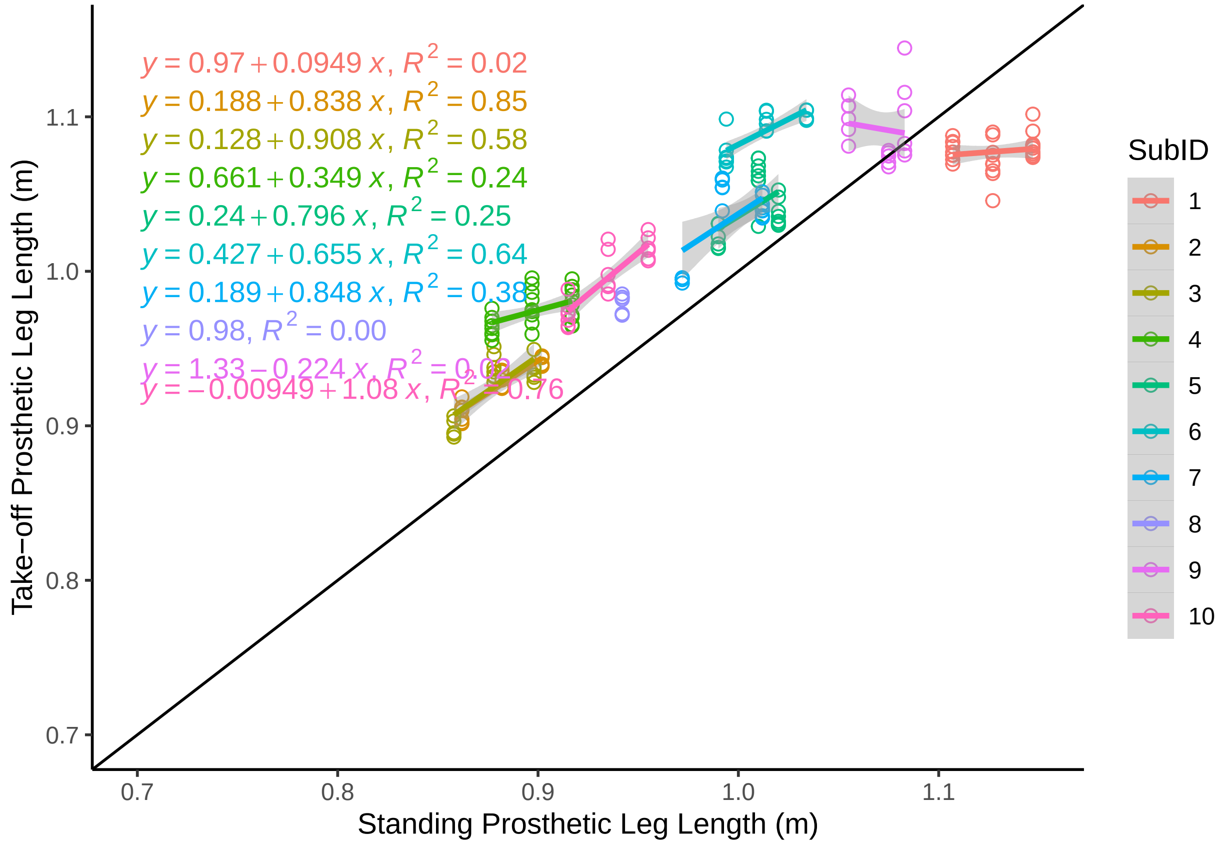


```
ggsave("uni_height_td.pdf", plot = unilat_vs_height_td+theme(legend.position = "none"), device = "pdf",
```

```
## Warning: Not enough data to perform fit for group 8; computing mean instead.
```

```
ggsave("uni_height_ms.pdf", plot = unilat_vs_height_ms+theme(legend.position = "none"), device = "pdf",
```

```
## Warning: Not enough data to perform fit for group 8; computing mean instead.
```

```
ggsave("uni_height_to.pdf", plot = unilat_vs_height_to+theme(legend.position = "none"), device = "pdf",
```

```
## Warning: Not enough data to perform fit for group 8; computing mean instead.
```

Bilateral Standing vs. Running Prosthetic Leg Length

```
#Touchdown
bilat_vs_height_td <- ggplot(data = bilateral_data, aes(x = Standing_adj, y = length_td, col = SubID))+
  geom_point(size = 2, alpha = 1, shape = 23)+
  geom_abline(slope = 1)+
  stat_poly_line(formula = y~x)+
  stat_poly_eq(method = "lm", formula = y~x, output.type = "expression", aes(label = paste(after_stat(e
  scale_y_continuous(limits = c(0.7,1.15))+
  scale_x_continuous(limits = c(0.7,1.15))+
  labs(x = "Standing Leg Length (m)", y = "Touchdown Leg Length (m)")+
  theme_classic()+
  theme(plot.title = element_text(hjust = 0.5))

bilat_vs_height_td
```

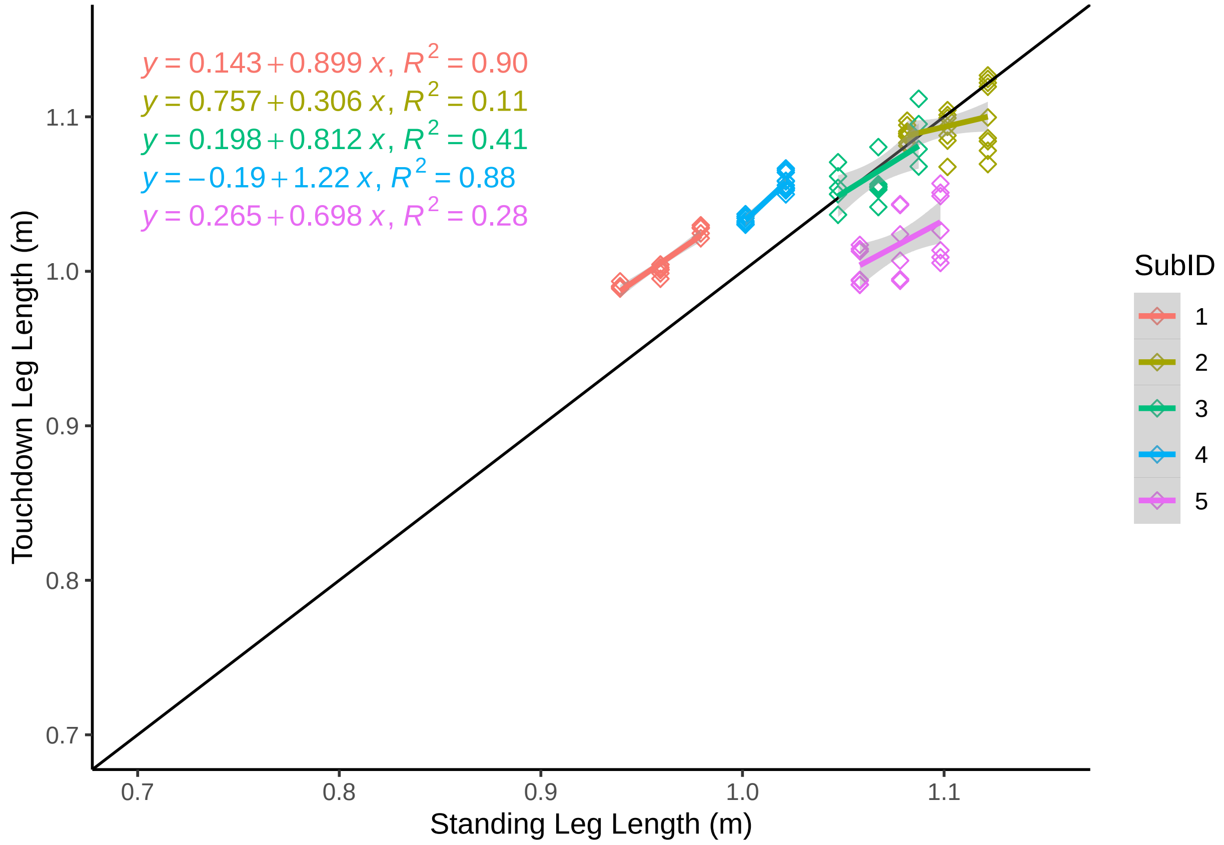


```
#Midstance
bilat_vs_height_ms <- ggplot(data = bilateral_data, aes(x = Standing_adj, y = length_ms, col = SubID))+
  geom_point(size = 2, alpha = 1, shape = 23)+
  geom_abline(slope = 1)+
  stat_poly_line(formula = y~x)+
  stat_poly_eq(method = "lm", formula = y~x, output.type = "expression", aes(label = paste(after_stat(e
  scale_y_continuous(limits = c(0.7,1.15))+
  scale_x_continuous(limits = c(0.7,1.15))+
  labs(x = "Standing Leg Length (m)", y = "Midstance Leg Length (m)")+
  theme_classic()+
  theme(plot.title = element_text(hjust = 0.5))

bilat_vs_height_ms
```

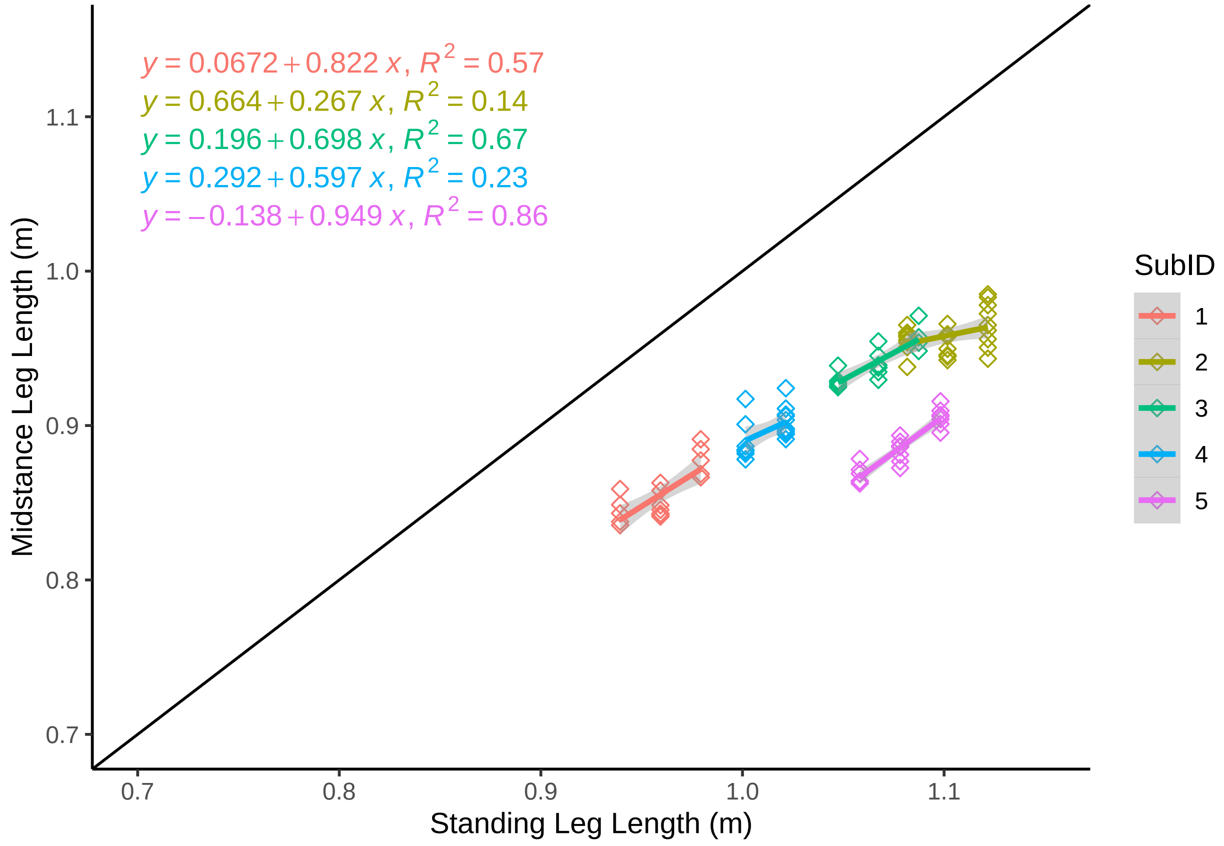


```
#Take-off
bilat_vs_height_to <- ggplot(data = bilateral_data, aes(x = Standing_adj, y = length_to, col = SubID))+
  geom_point(size = 2, alpha = 1, shape = 23)+
  geom_abline(slope = 1)+
  stat_poly_line(formula = y~x)+
  stat_poly_eq(method = "lm", formula = y~x, output.type = "expression", aes(label = paste(after_stat(e
  scale_y_continuous(limits = c(0.7,1.15))+
  scale_x_continuous(limits = c(0.7,1.15))+
  labs(x = "Standing Leg Length (m)", y = "Toe-off Leg Length (m)")+
  theme_classic()+
  theme(plot.title = element_text(hjust = 0.5))

bilat_vs_height_to
```

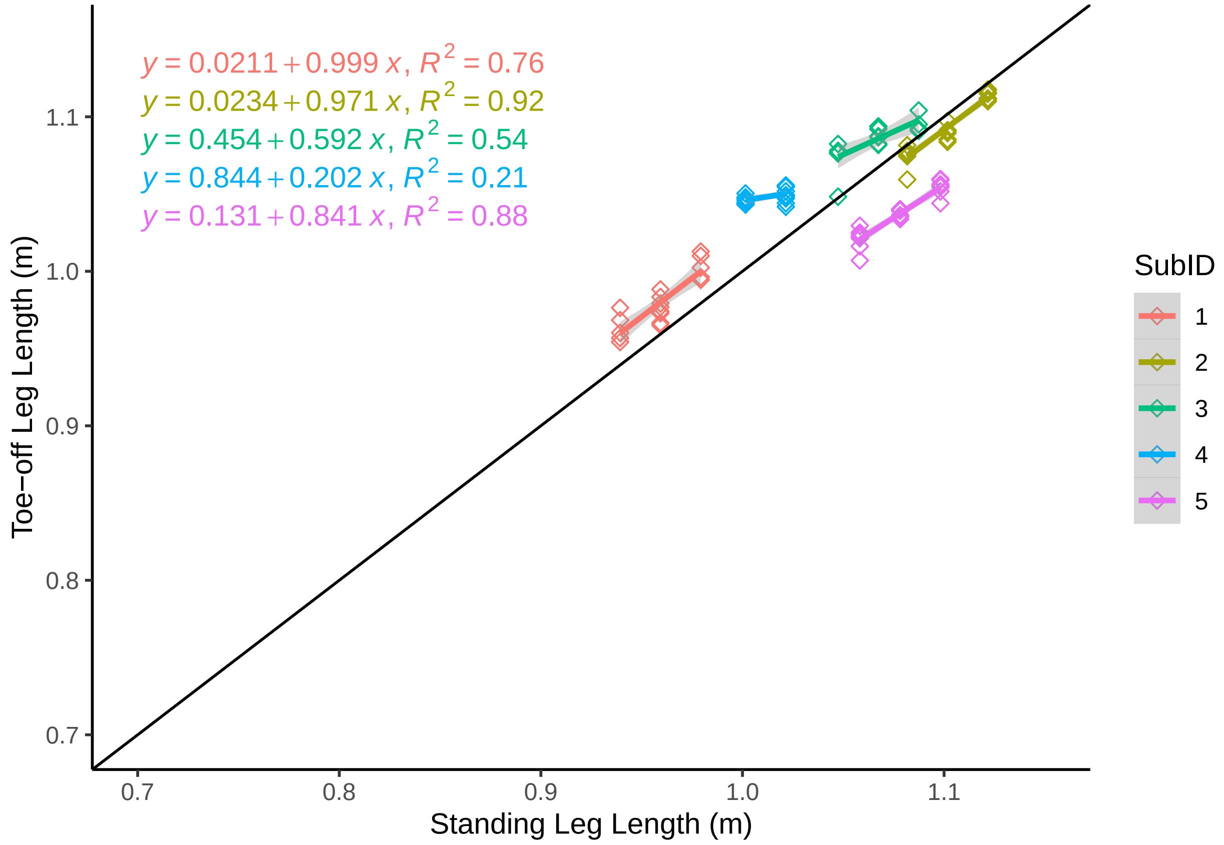


```
ggsave("height_td.pdf", plot = bilat_vs_height_td, device = "pdf", useDingbats = FALSE, width = 8, heig
ggsave("height_ms.pdf", plot = bilat_vs_height_ms, device = "pdf", useDingbats = FALSE, width = 8, heig
ggsave("height_to.pdf", plot = bilat_vs_height_to, device = "pdf", useDingbats = FALSE, width = 8, heig
```

Appendix Figure

```
#Appendix figure (RSP changing heights)
appen_uni <- plot_grid(unilat_vs_height_td+theme(legend.position = "none"), unilat_vs_height_ms+theme(le
```

```
## Warning: Not enough data to perform fit for group 8; computing mean instead.

## Warning: Not enough data to perform fit for group 8; computing mean instead.

## Warning: Not enough data to perform fit for group 8; computing mean instead.
```

```
appen_uni
```

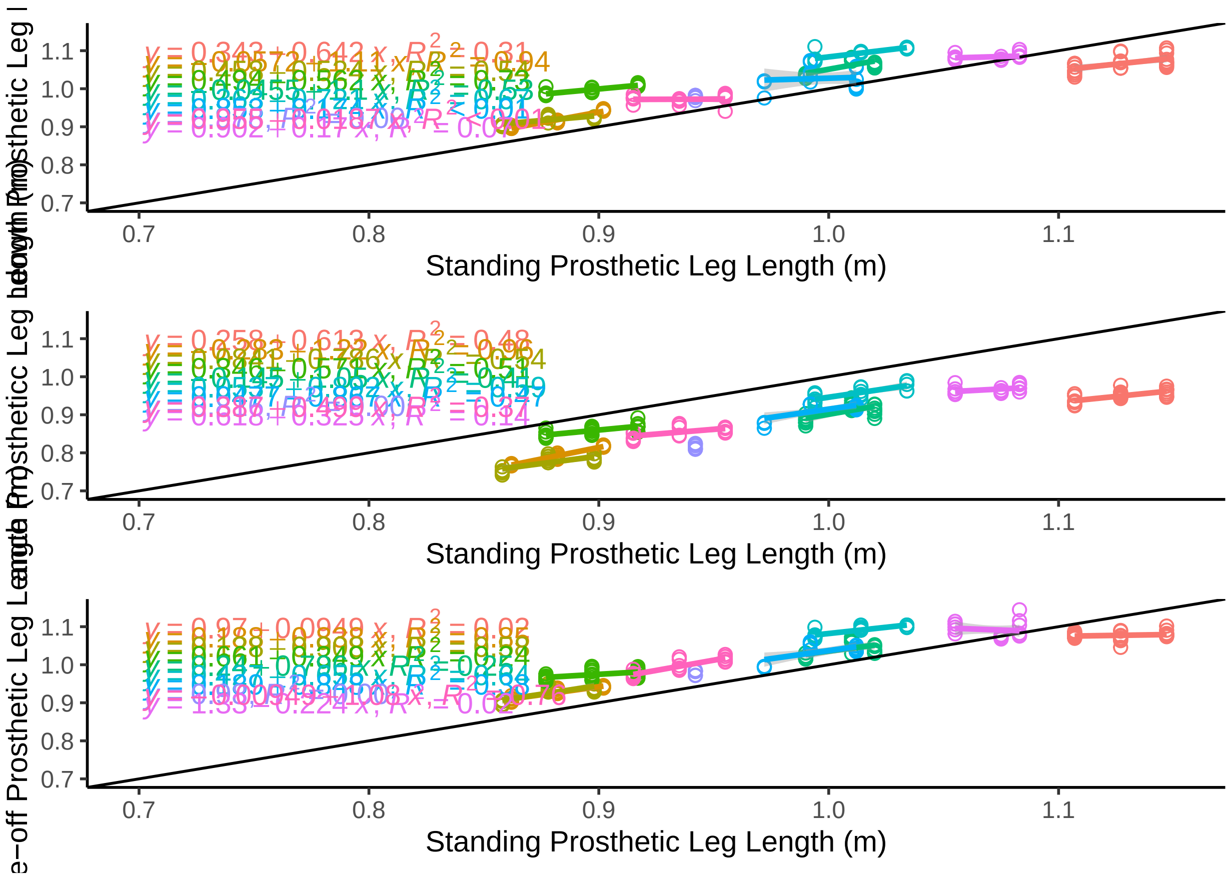


```
appen_bi <- plot_grid(bilat_vs_height_td+theme(legend.position = "none"), bilat_vs_height_ms+theme(legen
appen_bi
```

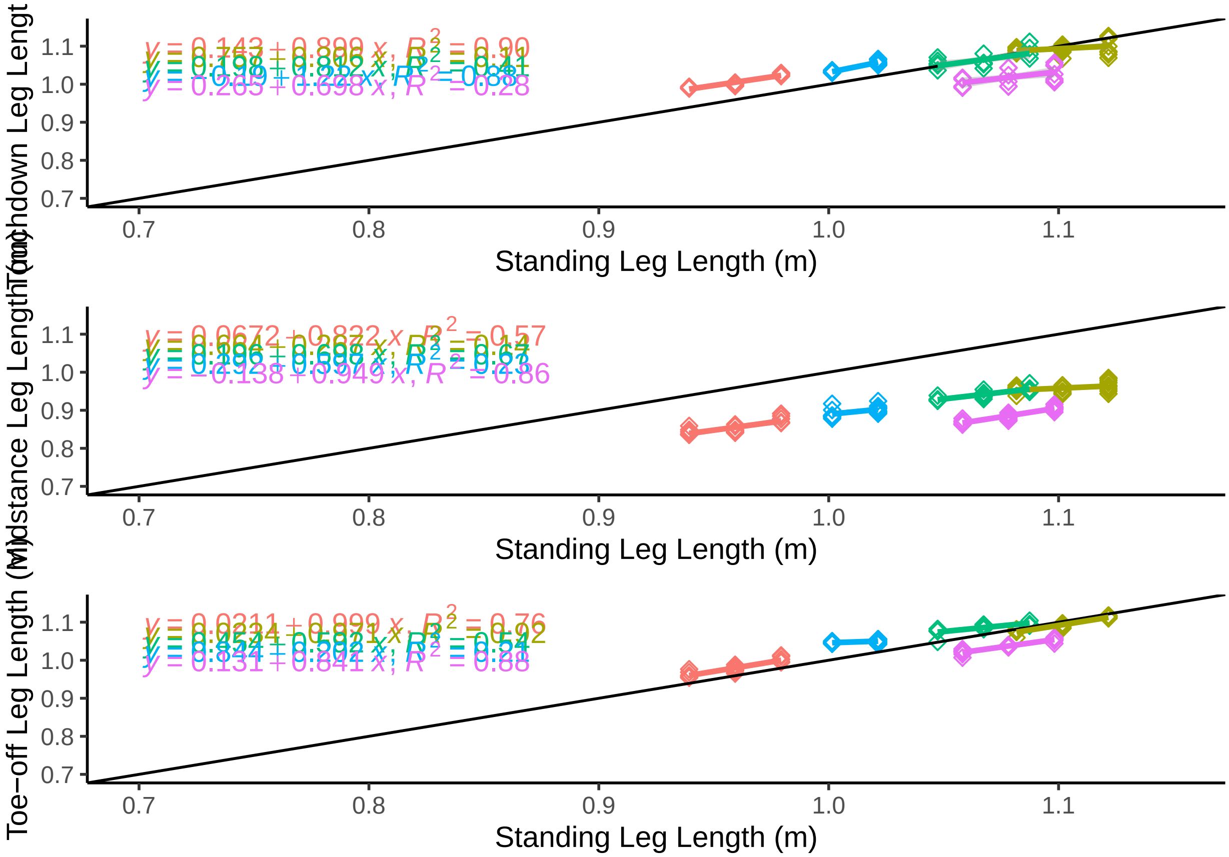


```
appen_fig <- plot_grid(appen_uni, appen_bi, ncol = 2, nrow = 1)
appen_fig
```

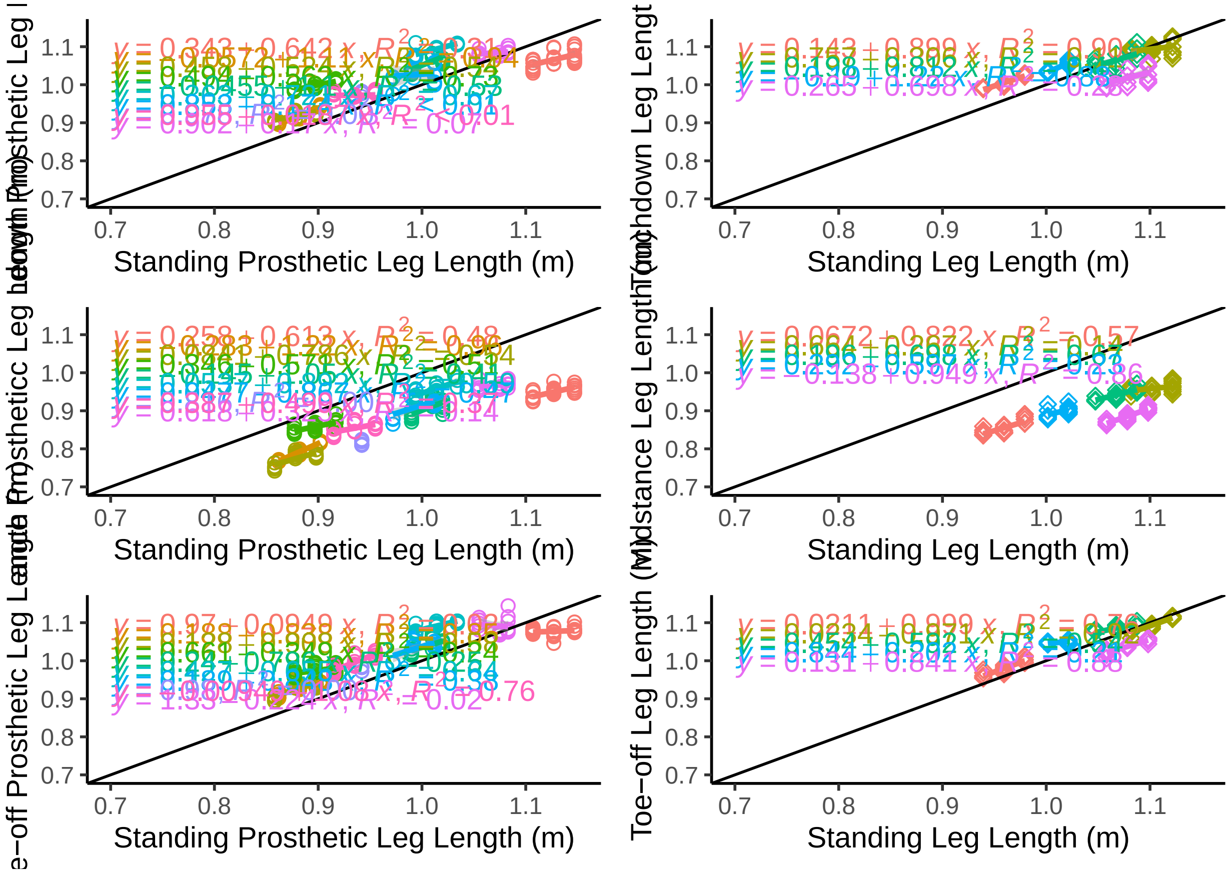


```
ggsave("appen_fig.pdf", plot = appen_fig, device = "pdf", useDingbats = FALSE, width = 10, height = 14.
```
