# Supplementary material for: Equivalent running leg lengths require prosthetic legs to be longer than biological legs during standing
Source: Sci Rep. 2023 May 11;13:7679. doi: 10.1038/s41598-023-34346-x (PMC10175537; doi:10.1038/s41598-023-34346-x)
Supplement: Supplementary file 2 — Supplementary Information 2. [file 41598_2023_34346_MOESM2_ESM.zip › Supplemental Materials/Stats.pdf]

Stats for 'Natural prosthetic leg lengths during standing yield unnaturally short legs during running'

Janet H. Zhang-Lea, Joshua R. Tacca, Owen N. Beck, Paolo Taboga, Alena M. Grabowski

05/26/2022

Load Packages

```
library("lme4") # Linear mixed effects model
```

```
## Loading required package: Matrix
```

```
library("performance")
library("effectsize")
```

```
## Registered S3 methods overwritten by 'parameters':
##   method                           from
##   as.double.parameters_kurtosis    datawizard
##   as.double.parameters_skewness    datawizard
##   as.double.parameters_smoothness  datawizard
##   as.numeric.parameters_kurtosis   datawizard
##   as.numeric.parameters_skewness   datawizard
##   as.numeric.parameters_smoothness datawizard
##   ci.blavaan                       bayestestR
##   print.parameters_distribution    datawizard
##   print.parameters_kurtosis        datawizard
##   print.parameters_skewness        datawizard
##   summary.parameters_kurtosis      datawizard
##   summary.parameters_skewness      datawizard
```

```
library("lmerTest")
```

```
##
## Attaching package: 'lmerTest'
```

```
## The following object is masked from 'package:lme4':
##
##     lmer
```

```
## The following object is masked from 'package:stats':
##
##     step
```

Load and Organize Data

```
# Load average data
rsp_height_data <- read.csv("Data.csv")

#Set subject ID as factor and create a column so that sub 1 from UL group is not same as sub 1 in NA gr
rsp_height_data$SubID <- as.factor(rsp_height_data$SubID)
rsp_height_data$SubAmp <- paste(rsp_height_data$SubID,rsp_height_data$AmpType)
```

```r
#Make sure height is imported as a numeric value
rsp_height_data$Height <- as.numeric(as.character(rsp_height_data$Height))
```

```
## Warning: NAs introduced by coercion
```

```r
#Bilateral subject 4 did not complete a trial at the recommended height, use the shortest height (-3cm)
rsp_height_data$Height_adj <- ifelse(rsp_height_data$AmpType == 'BL' & rsp_height_data$SubID == '4', rsp

#Add +- 2cm to standing for prosthetic legs for non-recommended height trials (standing leg length repo
rsp_height_data$Standing_adj <- ifelse(rsp_height_data$Leg.Type == 'PL', rsp_height_data$standing_leg_le

#calculate leg length ratio (*100 to set it in terms of percentage)
rsp_height_data$ratio_td <- round(rsp_height_data$length_td/rsp_height_data$Standing_adj,4)
rsp_height_data$ratio_ms <- round(rsp_height_data$length_ms/rsp_height_data$Standing_adj,4)
rsp_height_data$ratio_to <- round(rsp_height_data$length_to/rsp_height_data$Standing_adj,4)

#bilateral data
bilateral_data <- rsp_height_data[rsp_height_data$AmpType == "BL",]
bilateral_data.ht0 <- bilateral_data[bilateral_data$Height_adj == 0,]

#unilateral data
unilateral_data <- rsp_height_data[rsp_height_data$AmpType == "UL",]
unilateral_data.ht0 <- unilateral_data[unilateral_data$Height_adj == 0,]
unilateral_data.pl <- unilateral_data[unilateral_data$Leg.Type == "PL",]
unilateral_data.bioleg <- unilateral_data[unilateral_data$Leg.Type == "BioLeg",]

#non-amputee data
nonamputee_data <- rsp_height_data[rsp_height_data$AmpType == "NonAmp",]

#all data with just recommended height
rsp_height_data.ht0 <- rbind(bilateral_data.ht0, unilateral_data.ht0, nonamputee_data)

#combine non-amputee and bilateral data
bilatnonamp <- rbind(bilateral_data, nonamputee_data)
bilatnonamp.ht0 <- rbind(bilateral_data.ht0, nonamputee_data)

#data for standing leg length
standing_data = rsp_height_data[rsp_height_data$Standing == 1,]
standing_data_ul = standing_data[standing_data$AmpType == "UL",]
standing_data_bl = standing_data[standing_data$AmpType == "BL",]
standing_data_nonamp = standing_data[standing_data$AmpType == "NonAmp",]
standing_data_bilatnonamp = rbind(standing_data_bl, standing_data_nonamp)
```

Unilateral

Leg Length

```r
#Standing
#One-tailed paired t-test to see if standing prosthetic leg length is set longer than biological leg
LegLengthStanding <- t.test(standing_leg_length ~ Leg.Type, data = standing_data_ul, alternative = "less
LegLengthStanding
```

```
##
##  Paired t-test
##
```

```
## data:  standing_leg_length by Leg.Type
## t = -2.1597, df = 9, p-value = 0.02955
## alternative hypothesis: true difference in means is less than 0
## 95 percent confidence interval:
##          -Inf -0.003644471
## sample estimates:
## mean of the differences
##                 -0.0241
```

```
cohens_d(x = standing_data_ul[standing_data_ul$Leg.Type == 'PL',]$standing_leg_length, y = standing_data
```

```
## Cohen's d |        95% CI
## -------------------------
## 0.68      | [-0.03, 1.44]
```

```
#Touchdown
#Effect of leg type and running speed on leg length at touchdown
LegLengthTD <- lmer(length_td ~ Speed  + Leg.Type + Leg.Type*Speed + (1|SubID), data=unilateral_data.ht0
summary(LegLengthTD)
```

```
## Linear mixed model fit by maximum likelihood . t-tests use Satterthwaite's
##   method [lmerModLmerTest]
## Formula: length_td ~ Speed + Leg.Type + Leg.Type * Speed + (1 | SubID)
##    Data: unilateral_data.ht0
##
##      AIC      BIC   logLik deviance df.resid
##   -718.0   -700.5    365.0   -730.0      130
##
## Scaled residuals:
##      Min       1Q   Median       3Q      Max
## -3.11811 -0.51340  0.02732  0.52200  2.39590
##
## Random effects:
##  Groups   Name        Variance  Std.Dev.
##  SubID    (Intercept) 0.0042800 0.06542
##  Residual             0.0001787 0.01337
## Number of obs: 136, groups:  SubID, 10
##
## Fixed effects:
##                      Estimate Std. Error         df  t value Pr(>|t|)
## (Intercept)          1.010752   0.021266  11.092891   47.529 3.57e-14 ***
## Speed                0.002664   0.000761 126.034700    3.501 0.000642 ***
## Leg.TypePL           0.012696   0.006879 126.001229    1.846 0.067295 .
## Speed:Leg.TypePL    -0.004031   0.001053 126.001229   -3.827 0.000203 ***
## ---
## Signif. codes:  0 '***' 0.001 '**' 0.01 '*' 0.05 '.' 0.1 ' ' 1
##
## Correlation of Fixed Effects:
##             (Intr) Speed  Lg.TPL
## Speed       -0.218
## Leg.TypePL  -0.162  0.653
## Spd:Lg.TyPL  0.152 -0.692 -0.943
```

```
r2(LegLengthTD)
```

```
## # R2 for Mixed Models
```

```
##
##   Conditional R2: 0.960
##      Marginal R2: 0.013
```

```r
confint(LegLengthTD)
```

```
## Computing profile confidence intervals ...

##                          2.5 %       97.5 %
## .sig01             0.0445427030  0.109357469
## .sigma             0.0118741314  0.015206319
## (Intercept)        0.9651099093  1.056419645
## Speed              0.0011615389  0.004167581
## Leg.TypePL        -0.0008899923  0.026281519
## Speed:Leg.TypePL -0.0061115886 -0.001950573
```

```r
#Midstance
LegLengthMS<- lmer(length_ms ~ Speed  + Leg.Type + Leg.Type*Speed + (1|SubID), data=unilateral_data.ht0
summary(LegLengthMS)
```

```
## Linear mixed model fit by maximum likelihood . t-tests use Satterthwaite's
##   method [lmerModLmerTest]
## Formula: length_ms ~ Speed + Leg.Type + Leg.Type * Speed + (1 | SubID)
##    Data: unilateral_data.ht0
##
##      AIC      BIC   logLik deviance df.resid
##   -816.8   -799.3    414.4   -828.8      130
##
## Scaled residuals:
##     Min      1Q  Median      3Q     Max
## -2.2569 -0.6080 -0.1666  0.5566  2.7497
##
## Random effects:
##  Groups   Name        Variance  Std.Dev.
##  SubID    (Intercept) 4.376e-03 0.066151
##  Residual             8.148e-05 0.009027
## Number of obs: 136, groups:  SubID, 10
##
## Fixed effects:
##                    Estimate Std. Error         df t value Pr(>|t|)
## (Intercept)       9.103e-01  2.118e-02  1.048e+01  42.975 3.97e-13 ***
## Speed             6.620e-04  5.139e-04  1.260e+02   1.288 0.199979
## Leg.TypePL       -1.698e-02  4.645e-03  1.260e+02  -3.655 0.000376 ***
## Speed:Leg.TypePL -2.320e-03  7.113e-04  1.260e+02  -3.262 0.001425 **
## ---
## Signif. codes:  0 '***' 0.001 '**' 0.01 '*' 0.05 '.' 0.1 ' ' 1
##
## Correlation of Fixed Effects:
##             (Intr) Speed  Lg.TPL
## Speed       -0.148
## Leg.TypePL  -0.110  0.653
## Spd:Lg.TyPL  0.103 -0.692 -0.943
```

```r
r2(LegLengthMS)
```

```
## # R2 for Mixed Models
##
```

```
##   Conditional R2: 0.983
##      Marginal R2: 0.054
```

```r
confint(LegLengthMS)
```

```
## Computing profile confidence intervals ...

##                          2.5 %        97.5 %
## .sig01             0.0450839724  0.1105161523
## .sigma             0.0080173484  0.0102672348
## (Intercept)        0.8646092121  0.9559503382
## Speed             -0.0003527566  0.0016769779
## Leg.TypePL        -0.0261487318 -0.0078026666
## Speed:Leg.TypePL -0.0037247112 -0.0009152145
```

```r
#Take-off
LegLengthTO <- lmer(length_to ~ Speed  + Leg.Type + Leg.Type*Speed + (1|SubID), data=unilateral_data.ht
summary(LegLengthTO)
```

```
## Linear mixed model fit by maximum likelihood . t-tests use Satterthwaite's
##   method [lmerModLmerTest]
## Formula: length_to ~ Speed + Leg.Type + Leg.Type * Speed + (1 | SubID)
##    Data: unilateral_data.ht0
##
##      AIC      BIC   logLik deviance df.resid
##   -741.9   -724.4    376.9   -753.9      130
##
## Scaled residuals:
##     Min      1Q  Median      3Q     Max
## -2.8276 -0.5860 -0.0844  0.5784  3.2335
##
## Random effects:
##  Groups   Name        Variance  Std.Dev.
##  SubID    (Intercept) 0.0028832 0.05370
##  Residual             0.0001526 0.01235
## Number of obs: 136, groups:  SubID, 10
##
## Fixed effects:
##                    Estimate Std. Error        df t value Pr(>|t|)
## (Intercept)       1.025e+00  1.758e-02  1.139e+01  58.336 1.81e-15 ***
## Speed            -5.300e-04  7.031e-04  1.260e+02  -0.754 0.452417
## Leg.TypePL       -2.342e-02  6.356e-03  1.260e+02  -3.685 0.000339 ***
## Speed:Leg.TypePL  3.229e-03  9.733e-04  1.260e+02   3.318 0.001187 **
## ---
## Signif. codes:  0 '***' 0.001 '**' 0.01 '*' 0.05 '.' 0.1 ' ' 1
##
## Correlation of Fixed Effects:
##             (Intr) Speed  Lg.TPL
## Speed       -0.244
## Leg.TypePL  -0.181  0.653
## Spd:Lg.TyPL  0.170 -0.692 -0.943
```

```r
coef(summary(LegLengthTO))
```

```
##                       Estimate   Std. Error        df    t value     Pr(>|t|)
## (Intercept)       1.0254967189 0.0175790361  11.39149 58.3363454 1.810578e-15
## Speed            -0.0005299635 0.0007031215 126.04319 -0.7537296 4.524173e-01
```

```
## Leg.TypePL       -0.0234180680 0.0063556271 126.00082 -3.6846196 3.387434e-04
## Speed:Leg.TypePL  0.0032290186 0.0009732939 126.00082  3.3176192 1.186886e-03
```

```r
r2(LegLengthTO)
```

```
## # R2 for Mixed Models
##
##   Conditional R2: 0.950
##      Marginal R2: 0.007
```

```r
confint(LegLengthTO)
```

```
## Computing profile confidence intervals ...

##                        2.5 %        97.5 %
## .sig01             0.036540644  0.0897820308
## .sigma             0.010970916  0.0140496437
## (Intercept)        0.987838542  1.0631678706
## Speed             -0.001918586  0.0008587335
## Leg.TypePL        -0.035970414 -0.0108657214
## Speed:Leg.TypePL   0.001306766  0.0051512714
```

Leg Length Ratio (%)

```r
#Touchdown
#Effect of leg type and running speed on leg length at touchdown
LegRatioTD <- lmer(ratio_td ~ Speed  + Leg.Type + Leg.Type*Speed + (1|SubID), data=unilateral_data.ht0,
summary(LegRatioTD) #LegType*Speed significant
```

```
## Linear mixed model fit by maximum likelihood . t-tests use Satterthwaite's
##   method [lmerModLmerTest]
## Formula: ratio_td ~ Speed + Leg.Type + Leg.Type * Speed + (1 | SubID)
##    Data: unilateral_data.ht0
##
##      AIC      BIC   logLik deviance df.resid
##   -573.0   -555.5    292.5   -585.0      130
##
## Scaled residuals:
##      Min       1Q   Median       3Q      Max
## -1.85933 -0.86455 -0.02939  0.89456  1.67764
##
## Random effects:
##  Groups   Name        Variance  Std.Dev.
##  SubID    (Intercept) 0.0009286 0.03047
##  Residual             0.0006354 0.02521
## Number of obs: 136, groups:  SubID, 10
##
## Fixed effects:
##                   Estimate Std. Error         df t value Pr(>|t|)
## (Intercept)        1.065382   0.013378  32.152249  79.636   <2e-16 ***
## Speed              0.002712   0.001434 126.665795   1.892   0.0608 .
## Leg.TypePL        -0.015076   0.012970 126.155569  -1.162   0.2473
## Speed:Leg.TypePL  -0.003974   0.001986 126.155569  -2.001   0.0476 *
## ---
## Signif. codes:  0 '***' 0.001 '**' 0.01 '*' 0.05 '.' 0.1 ' ' 1
##
## Correlation of Fixed Effects:
```

```
##              (Intr) Speed  Lg.TPL
## Speed       -0.654
## Leg.TypePL  -0.485  0.653
## Spd:Lg.TyPL  0.457 -0.693 -0.943
```

```r
r2(LegRatioTD)
```

```
## # R2 for Mixed Models
##
##   Conditional R2: 0.679
##      Marginal R2: 0.210
```

```r
confint(LegRatioTD)
```

```
## Computing profile confidence intervals ...
```

```
##                            2.5 %        97.5 %
## .sig01             0.0202501397  5.160357e-02
## .sigma             0.0223904992  2.866942e-02
## (Intercept)        1.0382988510  1.092547e+00
## Speed             -0.0001183127  5.544371e-03
## Leg.TypePL        -0.0406924163  1.053985e-02
## Speed:Leg.TypePL -0.0078968209 -5.116722e-05
```

```r
#Midstance
#LegRatioMS <- lmer(ratio_ms ~ Speed  + Leg.Type + Leg.Type*Speed + (1|SubID), data=unilateral_data.ht0
#summary(LegRatioMS) #LegType*Speed interaction not significant
#LegRatioMS2 <- lmer(ratio_ms ~ Speed  + Leg.Type + (1|SubID), data=unilateral_data.ht0, REML=FALSE, na
#summary(LegRatioMS2)
LegRatioMS3 <- lmer(ratio_ms ~  Leg.Type + (1|SubID), data=unilateral_data.ht0, REML=FALSE, na.action=na
summary(LegRatioMS3)
```

```
## Linear mixed model fit by maximum likelihood . t-tests use Satterthwaite's
##   method [lmerModLmerTest]
## Formula: ratio_ms ~ Leg.Type + (1 | SubID)
##    Data: unilateral_data.ht0
##
##      AIC      BIC   logLik deviance df.resid
##   -651.9   -640.2    329.9   -659.9      132
##
## Scaled residuals:
##      Min       1Q   Median       3Q      Max
## -2.02031 -0.77595  0.00239  0.70511  2.26401
##
## Random effects:
##  Groups   Name        Variance  Std.Dev.
##  SubID    (Intercept) 0.0008460 0.02909
##  Residual             0.0003538 0.01881
## Number of obs: 136, groups:  SubID, 10
##
## Fixed effects:
##                Estimate Std. Error         df t value Pr(>|t|)
## (Intercept)   0.963044   0.009482  10.637007  101.57   <2e-16 ***
## Leg.TypePL   -0.056994   0.003226 126.031892  -17.67   <2e-16 ***
## ---
## Signif. codes:  0 '***' 0.001 '**' 0.01 '*' 0.05 '.' 0.1 ' ' 1
##
```

```
## Correlation of Fixed Effects:
##            (Intr)
## Leg.TypePL -0.170
```

```r
r2(LegRatioMS3)
```

```
## # R2 for Mixed Models
##
##   Conditional R2: 0.825
##      Marginal R2: 0.405
```

```r
confint(LegRatioMS3)
```

```
## Computing profile confidence intervals ...
```

```
##                   2.5 %      97.5 %
## .sig01       0.01948845  0.04904142
## .sigma       0.01670633  0.02139393
## (Intercept)  0.94262432  0.98346679
## Leg.TypePL  -0.06336506 -0.05062317
```

```r
#Take-off
#LegRatioTO <- lmer(ratio_to ~ Speed  + Leg.Type + Leg.Type*Speed + (1|SubID), data=unilateral_data.ht0
#summary(LegRatioTO) #LegType*Speed interaction not significant
#LegRatioTO2 <- lmer(ratio_to ~ Speed  + Leg.Type + (1|SubID), data=unilateral_data.ht0, REML=FALSE, na
#summary(LegRatioTO2)
LegRatioTO3 <- lmer(ratio_to ~ Leg.Type + (1|SubID), data=unilateral_data.ht0, REML=FALSE, na.action=na
summary(LegRatioTO3)
```

```
## Linear mixed model fit by maximum likelihood . t-tests use Satterthwaite's
##   method [lmerModLmerTest]
## Formula: ratio_to ~ Leg.Type + (1 | SubID)
##    Data: unilateral_data.ht0
##
##      AIC      BIC   logLik deviance df.resid
##   -593.4   -581.8    300.7   -601.4      132
##
## Scaled residuals:
##      Min       1Q   Median       3Q      Max
## -2.15607 -0.75068 -0.03273  0.70661  2.12291
##
## Random effects:
##  Groups   Name        Variance  Std.Dev.
##  SubID    (Intercept) 0.0011916 0.03452
##  Residual             0.0005474 0.02340
## Number of obs: 136, groups:  SubID, 10
##
## Fixed effects:
##               Estimate Std. Error         df t value Pr(>|t|)
## (Intercept)   1.078043   0.011286  10.760410  95.521  < 2e-16 ***
## Leg.TypePL   -0.031557   0.004012 126.092608  -7.865 1.43e-12 ***
## ---
## Signif. codes:  0 '***' 0.001 '**' 0.01 '*' 0.05 '.' 0.1 ' ' 1
##
## Correlation of Fixed Effects:
##            (Intr)
## Leg.TypePL -0.178
```

```r
r2(LegRatioTO3)
```

```
## # R2 for Mixed Models
##
##   Conditional R2: 0.725
##      Marginal R2: 0.126
```

```r
confint(LegRatioTO3)
```

```
## Computing profile confidence intervals ...

##                   2.5 %      97.5 %
## .sig01       0.02312962  0.05820293
## .sigma       0.02078053  0.02660967
## (Intercept)  1.05376477  1.10233473
## Leg.TypePL  -0.03948176 -0.02363295
```

Unilateral Affected Running Leg Length when RSP Height Adjusted by ±2cm

```r
#Only use affected leg lengths

#Touchdown
LegLengthTD_height_al <- lmer(length_td ~ Standing_adj*Speed + (1|SubID), data=unilateral_data[unilatera
summary(LegLengthTD_height_al)
```

```
## Linear mixed model fit by maximum likelihood . t-tests use Satterthwaite's
##   method [lmerModLmerTest]
## Formula: length_td ~ Standing_adj * Speed + (1 | SubID)
##    Data: unilateral_data[unilateral_data$Leg.Type == "PL", ]
##
##      AIC      BIC   logLik deviance df.resid
##  -1068.7  -1049.1    540.3  -1080.7      187
##
## Scaled residuals:
##     Min      1Q  Median      3Q     Max
## -3.3600 -0.5002 -0.1295  0.5994  3.7012
##
## Random effects:
##  Groups   Name        Variance  Std.Dev.
##  SubID    (Intercept) 0.0009366 0.03060
##  Residual             0.0001706 0.01306
## Number of obs: 193, groups:  SubID, 10
##
## Fixed effects:
##                     Estimate Std. Error         df t value Pr(>|t|)
## (Intercept)         0.312858   0.062153 145.650926   5.034 1.40e-06 ***
## Standing_adj        0.721073   0.062765 156.588572  11.489  < 2e-16 ***
## Speed               0.018811   0.004706 183.201049   3.997 9.27e-05 ***
## Standing_adj:Speed -0.020014   0.004716 183.203244  -4.244 3.49e-05 ***
## ---
## Signif. codes:  0 '***' 0.001 '**' 0.01 '*' 0.05 '.' 0.1 ' ' 1
##
## Correlation of Fixed Effects:
##             (Intr) Stndn_ Speed
## Standing_dj -0.987
## Speed       -0.465  0.461
```

```
## Stndng_dj:S  0.466 -0.466 -0.996
```

```
r2(LegLengthTD_height_al)
```

```
## # R2 for Mixed Models
##
##   Conditional R2: 0.954
##      Marginal R2: 0.704
```

```
#Mid-stance
#LegLengthMS_height_al <- lmer(length_ms ~ Standing_adj*Speed + (1|SubID), data=unilateral_data[unilate
#summary(LegLengthMS_height_al) #speed and interaction not significant
LegLengthMS_height_al2 <- lmer(length_ms ~ Standing_adj + (1|SubID), data=unilateral_data[unilateral_da
summary(LegLengthMS_height_al2)
```

```
## Linear mixed model fit by maximum likelihood . t-tests use Satterthwaite's
##   method [lmerModLmerTest]
## Formula: length_ms ~ Standing_adj + (1 | SubID)
##    Data: unilateral_data[unilateral_data$Leg.Type == "PL", ]
##
##      AIC      BIC   logLik deviance df.resid
##  -1108.4  -1095.4    558.2  -1116.4      189
##
## Scaled residuals:
##     Min      1Q  Median      3Q     Max
## -2.4364 -0.6777 -0.1230  0.6613  2.8420
##
## Random effects:
##  Groups   Name        Variance  Std.Dev.
##  SubID    (Intercept) 0.0009083 0.03014
##  Residual             0.0001406 0.01186
## Number of obs: 193, groups:  SubID, 10
##
## Fixed effects:
##               Estimate Std. Error        df t value Pr(>|t|)
## (Intercept)    0.14652    0.05079 129.53306   2.885  0.00459 **
## Standing_adj   0.75131    0.05116 145.83364  14.685  < 2e-16 ***
## ---
## Signif. codes:  0 '***' 0.001 '**' 0.01 '*' 0.05 '.' 0.1 ' ' 1
##
## Correlation of Fixed Effects:
##             (Intr)
## Standing_dj -0.982
```

```
r2(LegLengthMS_height_al2)
```

```
## # R2 for Mixed Models
##
##   Conditional R2: 0.973
##      Marginal R2: 0.802
```

```
#Take-off
#LegLengthTO_height_al <- lmer(length_to ~ Standing_adj*Speed + (1|SubID), data=unilateral_data[unilate
#summary(LegLengthTO_height_al) #interaction not significant
LegLengthTO_height_al2 <- lmer(length_to ~ Standing_adj + Speed + (1|SubID), data=unilateral_data[unila
summary(LegLengthTO_height_al2)
```

```
## Linear mixed model fit by maximum likelihood . t-tests use Satterthwaite's
##   method [lmerModLmerTest]
## Formula: length_to ~ Standing_adj + Speed + (1 | SubID)
##    Data: unilateral_data[unilateral_data$Leg.Type == "PL", ]
##
##      AIC      BIC   logLik deviance df.resid
##  -1063.5  -1047.2    536.7  -1073.5      188
##
## Scaled residuals:
##      Min       1Q   Median       3Q      Max
## -2.07779 -0.70696 -0.06493  0.59439  3.14059
##
## Random effects:
##  Groups   Name        Variance  Std.Dev.
##  SubID    (Intercept) 0.0006279 0.02506
##  Residual             0.0001813 0.01347
## Number of obs: 193, groups:  SubID, 10
##
## Fixed effects:
##               Estimate Std. Error        df t value Pr(>|t|)
## (Intercept)  4.112e-01  5.341e-02 8.323e+01   7.699 2.54e-11 ***
## Standing_adj 6.071e-01  5.418e-02 8.936e+01  11.206  < 2e-16 ***
## Speed        1.773e-03  4.511e-04 1.831e+02   3.931  0.00012 ***
## ---
## Signif. codes:  0 '***' 0.001 '**' 0.01 '*' 0.05 '.' 0.1 ' ' 1
##
## Correlation of Fixed Effects:
##             (Intr) Stndn_
## Standing_dj -0.987
## Speed       -0.018 -0.034
```

```
r2(LegLengthTO_height_al2)
```

```
## # R2 for Mixed Models
##
##   Conditional R2: 0.950
##      Marginal R2: 0.778
```

People with bilateral amputations and non-amputees

Leg Length Ratio

```
#Touchdown
#Effect of leg type and running speed on leg ratio at touchdown
LegRatioTDAll <- lmer(ratio_td ~ Speed + AmpType + AmpType*Speed + (1|SubAmp), data=bilatnonamp.ht0, REI
summary(LegRatioTDAll)
```

```
## Linear mixed model fit by maximum likelihood . t-tests use Satterthwaite's
##   method [lmerModLmerTest]
## Formula: ratio_td ~ Speed + AmpType + AmpType * Speed + (1 | SubAmp)
##    Data: bilatnonamp.ht0
##
##      AIC      BIC   logLik deviance df.resid
##     -558     -542      285     -570      101
##
## Scaled residuals:
```

```
##      Min       1Q   Median       3Q      Max
## -3.13029 -0.40807  0.07332  0.49468  2.90372
##
## Random effects:
##  Groups   Name        Variance  Std.Dev.
##  SubAmp   (Intercept) 0.0008586 0.02930
##  Residual             0.0001674 0.01294
## Number of obs: 107, groups:  SubAmp, 16
##
## Fixed effects:
##                       Estimate Std. Error         df t value Pr(>|t|)
## (Intercept)          1.0140533  0.0144836 22.4292697  70.014   <2e-16 ***
## Speed               -0.0015985  0.0008766 91.4042761  -1.823   0.0715 .
## AmpTypeNonAmp       -0.0014476  0.0177453 23.8162378  -0.082   0.9357
## Speed:AmpTypeNonAmp  0.0031592  0.0012027 91.4168359   2.627   0.0101 *
## ---
## Signif. codes:  0 '***' 0.001 '**' 0.01 '*' 0.05 '.' 0.1 ' ' 1
##
## Correlation of Fixed Effects:
##             (Intr) Speed  AmpTNA
## Speed       -0.397
## AmpTypNnAmp -0.816  0.324
## Spd:AmpTyNA  0.289 -0.729 -0.428
```

```
r2(LegRatioTDAll)
```

```
## # R2 for Mixed Models
##
##   Conditional R2: 0.850
##      Marginal R2: 0.082
```

```
confint(LegRatioTDAll)
```

```
## Computing profile confidence intervals ...
```

```
##                             2.5 %       97.5 %
## .sig01               0.0212112521 0.0436792753
## .sigma               0.0112658561 0.0150731057
## (Intercept)          0.9843530310 1.0437502549
## Speed               -0.0033342883 0.0001386175
## AmpTypeNonAmp       -0.0376932495 0.0349018844
## Speed:AmpTypeNonAmp  0.0007778865 0.0055426801
```

```
#Midstance
LegRatioMSAll <- lmer(ratio_ms ~ Speed + AmpType + AmpType*Speed + (1|SubAmp), data=bilatnonamp.ht0, RE[illegible]
summary(LegRatioMSAll)
```

```
## Linear mixed model fit by maximum likelihood . t-tests use Satterthwaite's
##   method [lmerModLmerTest]
## Formula: ratio_ms ~ Speed + AmpType + AmpType * Speed + (1 | SubAmp)
##    Data: bilatnonamp.ht0
##
##      AIC      BIC   logLik deviance df.resid
##   -726.3   -710.3    369.2   -738.3      101
##
## Scaled residuals:
##     Min      1Q  Median      3Q     Max
```

```
## -2.3480 -0.5931 -0.0866  0.5323  3.4523
##
## Random effects:
##  Groups   Name        Variance  Std.Dev.
##  SubAmp   (Intercept) 4.045e-04 0.020111
##  Residual             3.017e-05 0.005493
## Number of obs: 107, groups:  SubAmp, 16
##
## Fixed effects:
##                        Estimate Std. Error         df t value Pr(>|t|)
## (Intercept)           0.8853919  0.0093675 18.3967573  94.517  < 2e-16 ***
## Speed                -0.0029090  0.0003723 91.1497873  -7.813 9.23e-12 ***
## AmpTypeNonAmp         0.0247633  0.0113755 18.9000249   2.177   0.0424 *
## Speed:AmpTypeNonAmp   0.0045469  0.0005108 91.1547192   8.901 5.02e-14 ***
## ---
## Signif. codes:  0 '***' 0.001 '**' 0.01 '*' 0.05 '.' 0.1 ' ' 1
##
## Correlation of Fixed Effects:
##             (Intr) Speed  AmpTNA
## Speed       -0.260
## AmpTypNnAmp -0.823  0.214
## Spd:AmpTyNA  0.190 -0.729 -0.283
```

```
r2(LegRatioMSAll)
```

```
## # R2 for Mixed Models
##
##   Conditional R2: 0.973
##      Marginal R2: 0.616
```

```
confint(LegRatioMSAll)
```

```
## Computing profile confidence intervals ...
```

```
##                            2.5 %       97.5 %
## .sig01                0.014669307  0.029840236
## .sigma                0.004781829  0.006398352
## (Intercept)           0.866006501  0.904768534
## Speed                -0.003646660 -0.002171668
## AmpTypeNonAmp         0.001273674  0.048283433
## Speed:AmpTypeNonAmp   0.003535519  0.005559288
```

```
#Take-off
LegRatioTOAll <- lmer(ratio_to ~ Speed  + AmpType + AmpType*Speed + (1|SubAmp), data=bilatnonamp.ht0, R
summary(LegRatioTOAll)
```

```
## Linear mixed model fit by maximum likelihood . t-tests use Satterthwaite's
##   method [lmerModLmerTest]
## Formula: ratio_to ~ Speed + AmpType + AmpType * Speed + (1 | SubAmp)
##    Data: bilatnonamp.ht0
##
##      AIC      BIC   logLik deviance df.resid
##   -732.5   -716.4    372.2   -744.5      101
##
## Scaled residuals:
##      Min       1Q   Median       3Q      Max
## -2.39429 -0.62018  0.06242  0.70421  2.27107
```

```
##
## Random effects:
##  Groups   Name        Variance  Std.Dev.
##  SubAmp   (Intercept) 4.131e-04 0.020325
##  Residual             2.811e-05 0.005301
## Number of obs: 107, groups:  SubAmp, 16
##
## Fixed effects:
##                       Estimate Std. Error         df t value Pr(>|t|)
## (Intercept)          1.0029547  0.0094343 18.1959323 106.309  < 2e-16 ***
## Speed               -0.0003279  0.0003594 91.1481277  -0.912   0.3639
## AmpTypeNonAmp        0.0276770  0.0114502 18.6536049   2.417   0.0261 *
## Speed:AmpTypeNonAmp  0.0020423  0.0004931 91.1526297   4.142 7.69e-05 ***
## ---
## Signif. codes:  0 '***' 0.001 '**' 0.01 '*' 0.05 '.' 0.1 ' ' 1
##
## Correlation of Fixed Effects:
##             (Intr) Speed  AmpTNA
## Speed       -0.250
## AmpTypNnAmp -0.824  0.206
## Spd:AmpTyNA  0.182 -0.729 -0.272
```

```
r2(LegRatioTOAll)
```

```
## # R2 for Mixed Models
##
##   Conditional R2: 0.966
##      Marginal R2: 0.461
```

```
confint(LegRatioTOAll)
```

```
## Computing profile confidence intervals ...
```

```
##                            2.5 %       97.5 %
## .sig01               0.014833531 0.0301457517
## .sigma               0.004615512 0.0061757102
## (Intercept)          0.983425420 1.0224847128
## Speed               -0.001039677 0.0003840482
## AmpTypeNonAmp        0.004011941 0.0513602836
## Speed:AmpTypeNonAmp  0.001065837 0.0030192078
```

Bilateral Running Leg Length when RSP Height Adjusted by ±2cm

```
#Touchdown
bilat_height.td <- lmer(length_td ~ Speed*Standing_adj  + (1|SubID), data=bilateral_data, REML=FALSE, na
summary(bilat_height.td)
```

```
## Linear mixed model fit by maximum likelihood . t-tests use Satterthwaite's
##   method [lmerModLmerTest]
## Formula: length_td ~ Speed * Standing_adj + (1 | SubID)
##    Data: bilateral_data
##
##      AIC      BIC   logLik deviance df.resid
##   -554.4   -538.9    283.2   -566.4       92
##
## Scaled residuals:
##      Min       1Q   Median       3Q      Max
```

```
## -2.49335 -0.62517 -0.02549  0.65551  2.31677
##
## Random effects:
##  Groups   Name        Variance  Std.Dev.
##  SubID    (Intercept) 0.0006622 0.02573
##  Residual             0.0001437 0.01199
## Number of obs: 98, groups:  SubID, 5
##
## Fixed effects:
##                     Estimate Std. Error       df t value Pr(>|t|)
## (Intercept)          0.13925    0.10496 96.46576   1.327 0.187732
## Speed                0.03652    0.01104 92.90147   3.308 0.001336 **
## Standing_adj         0.88214    0.09974 97.44795   8.844 4.04e-14 ***
## Speed:Standing_adj -0.03708    0.01049 92.89341  -3.536 0.000636 ***
## ---
## Signif. codes:  0 '***' 0.001 '**' 0.01 '*' 0.05 '.' 0.1 ' ' 1
##
## Correlation of Fixed Effects:
##             (Intr) Speed  Stndn_
## Speed       -0.645
## Standing_dj -0.993  0.644
## Spd:Stndng_  0.646 -0.999 -0.647
```

```
r2(bilat_height.td)
```

```
## # R2 for Mixed Models
##
##   Conditional R2: 0.931
##      Marginal R2: 0.611
```

```
#Mid-stance
#bilat_height.ms <- lmer(length_ms ~ Speed*Standing_adj + (1|SubID), data=bilateral_data, REML=FALSE, n
#summary(bilat_height.ms) #speed and interaction  not significant
bilat_height.ms2 <- lmer(length_ms ~ Standing_adj + (1|SubID), data=bilateral_data, REML=FALSE, na.acti
summary(bilat_height.ms2)
```

```
## Linear mixed model fit by maximum likelihood . t-tests use Satterthwaite's
##   method [lmerModLmerTest]
## Formula: length_ms ~ Standing_adj + (1 | SubID)
##    Data: bilateral_data
##
##      AIC      BIC   logLik deviance df.resid
##   -582.5   -572.2    295.3   -590.5       94
##
## Scaled residuals:
##      Min       1Q   Median       3Q      Max
## -2.59463 -0.71409 -0.08923  0.71260  2.58186
##
## Random effects:
##  Groups   Name        Variance  Std.Dev.
##  SubID    (Intercept) 0.0005071 0.02252
##  Residual             0.0001126 0.01061
## Number of obs: 98, groups:  SubID, 5
##
## Fixed effects:
```

```
##               Estimate Std. Error       df t value Pr(>|t|)
## (Intercept)   0.25010    0.07083 86.02540   3.531 0.000668 ***
## Standing_adj  0.62986    0.06716 90.34058   9.379 5.42e-15 ***
## ---
## Signif. codes:  0 '***' 0.001 '**' 0.01 '*' 0.05 '.' 0.1 ' ' 1
##
## Correlation of Fixed Effects:
##             (Intr)
## Standing_dj -0.990
```

```
r2(bilat_height.ms2)
```

```
## # R2 for Mixed Models
##
##   Conditional R2: 0.936
##      Marginal R2: 0.650
```

```
#Take-off
bilat_height.to <- lmer(length_to ~ Speed*Standing_adj  + (1|SubID), data=bilateral_data, REML=FALSE, na
summary(bilat_height.to)
```

```
## Linear mixed model fit by maximum likelihood . t-tests use Satterthwaite's
##   method [lmerModLmerTest]
## Formula: length_to ~ Speed * Standing_adj + (1 | SubID)
##    Data: bilateral_data
##
##      AIC      BIC   logLik deviance df.resid
##   -657.7   -642.2    334.9   -669.7       92
##
## Scaled residuals:
##      Min       1Q   Median       3Q      Max
## -3.16089 -0.47184  0.08768  0.72871  1.92679
##
## Random effects:
##  Groups   Name        Variance  Std.Dev.
##  SubID    (Intercept) 5.060e-04 0.022495
##  Residual             4.805e-05 0.006932
## Number of obs: 98, groups:  SubID, 5
##
## Fixed effects:
##                     Estimate Std. Error        df t value Pr(>|t|)
## (Intercept)         0.305840   0.062329 97.912989   4.907 3.69e-06 ***
## Speed              -0.017742   0.006384 92.997583  -2.779  0.00659 **
## Standing_adj        0.714416   0.058814 97.295037  12.147  < 2e-16 ***
## Speed:Standing_adj  0.016568   0.006064 92.994153   2.732  0.00753 **
## ---
## Signif. codes:  0 '***' 0.001 '**' 0.01 '*' 0.05 '.' 0.1 ' ' 1
##
## Correlation of Fixed Effects:
##             (Intr) Speed  Stndn_
## Speed       -0.628
## Standing_dj -0.986  0.633
## Spd:Stndng_  0.629 -0.999 -0.635
```

```
r2(bilat_height.to)
```

```
## # R2 for Mixed Models
##
##   Conditional R2: 0.981
##      Marginal R2: 0.777
```
