# Supplementary figures and images for: Equivalent running leg lengths require prosthetic legs to be longer than biological legs during standing
Source: Sci Rep. 2023 May 11;13:7679. doi: 10.1038/s41598-023-34346-x (PMC10175537; doi:10.1038/s41598-023-34346-x)

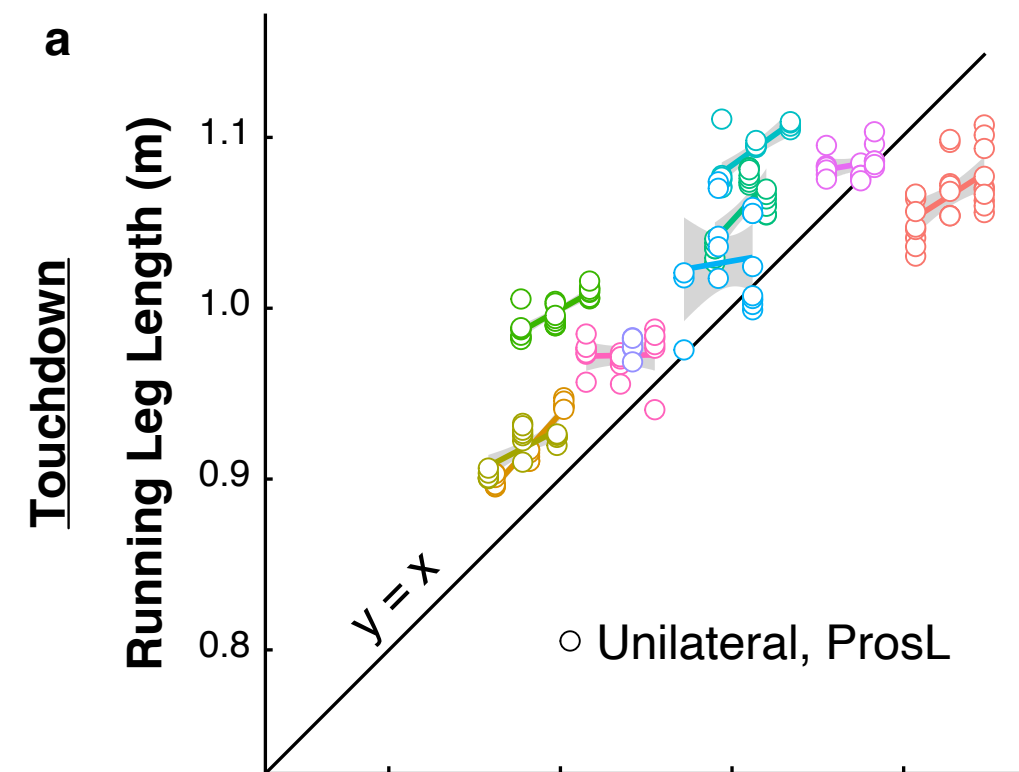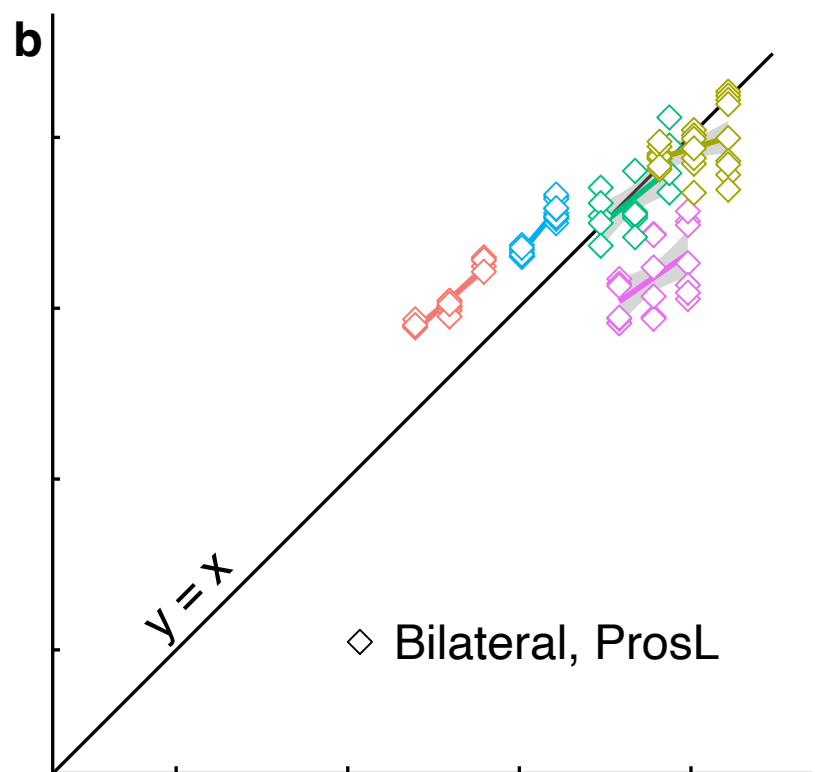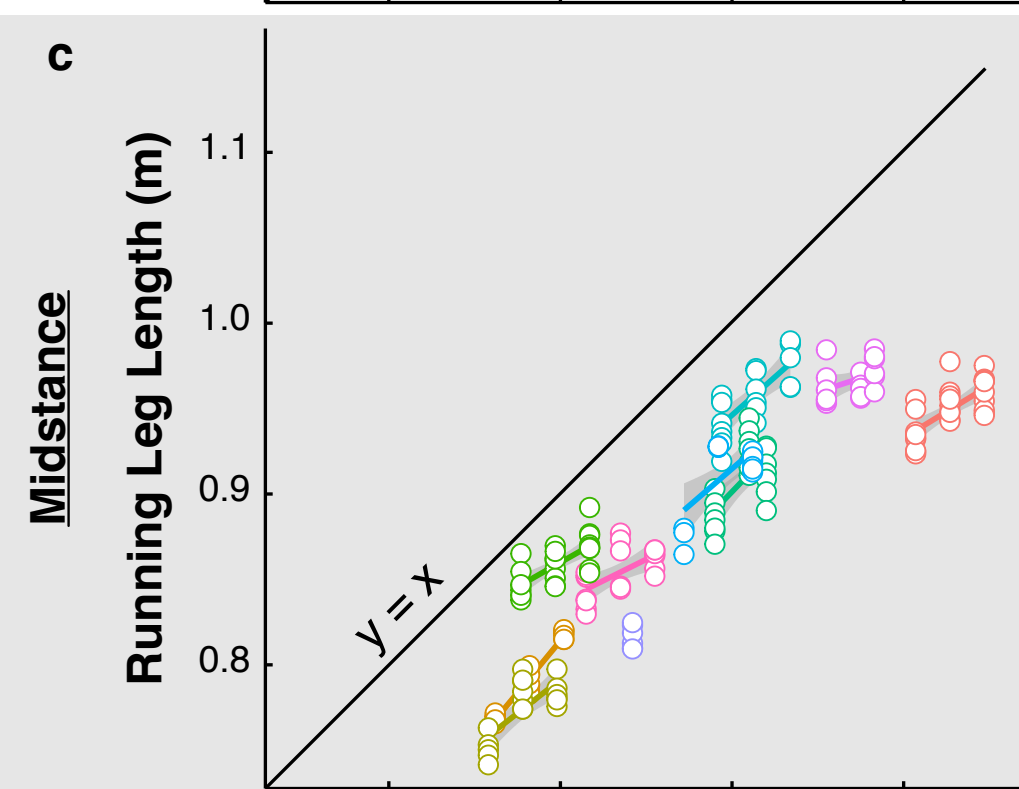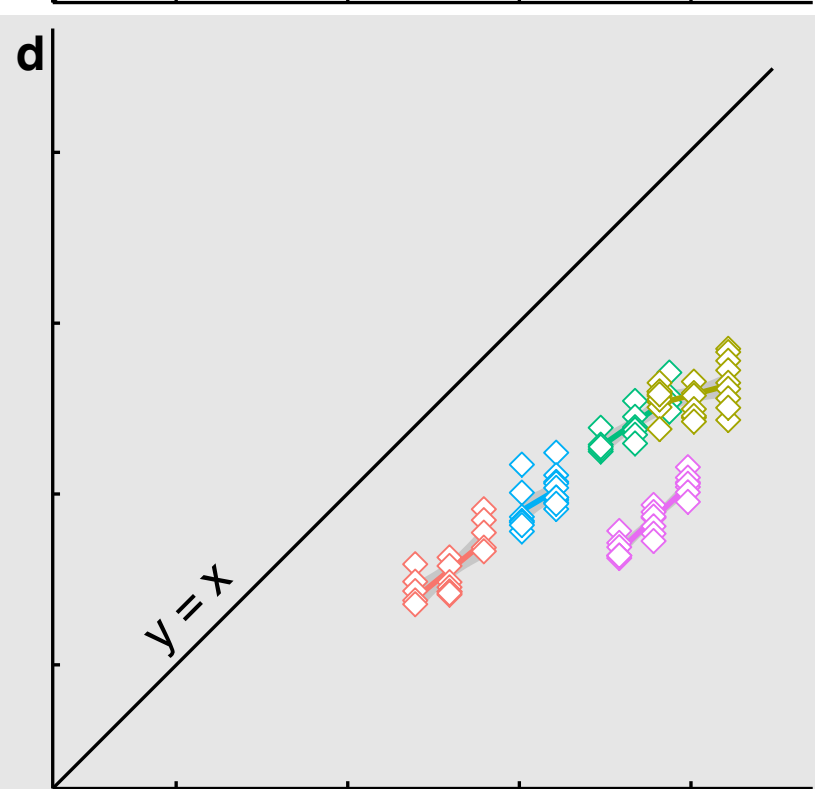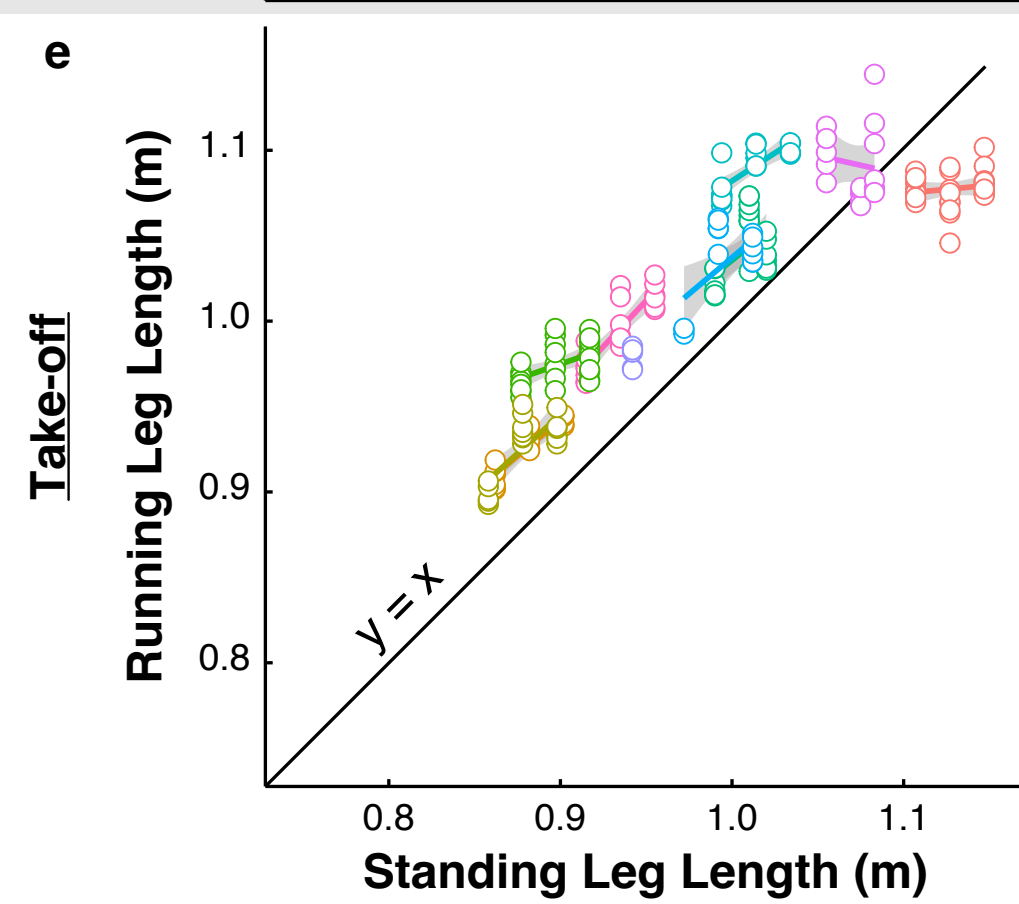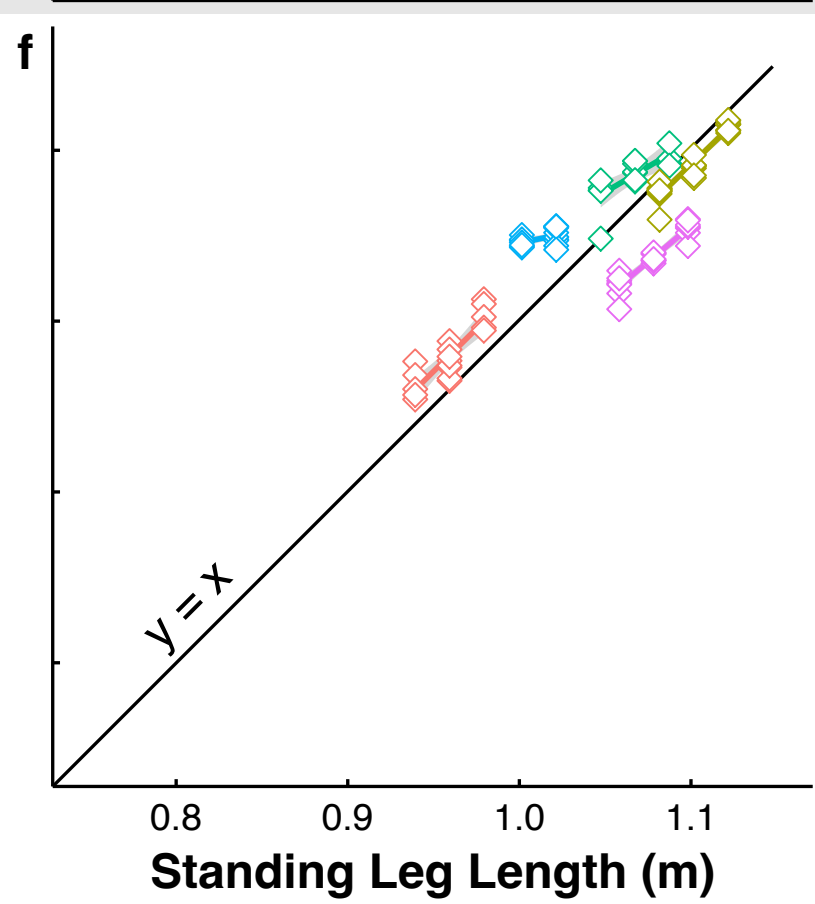

Supplement: Supplementary file 3 — Supplementary Information 3. [file 41598_2023_34346_MOESM3_ESM.pdf]
